# Psychosocial correlates of children’s football participation: a psychological network analysis of parental support, stereotypes, and dualistic passions

**DOI:** 10.3389/fpsyg.2026.1897362

**Published:** 2026-07-01

**Authors:** Xiancheng Zeng, Min Hu, Wei He, Zechen Xie

**Affiliations:** 1 Guangzhou Sport University, Guangzhou, China; 2 Macao Polytechnic University, Macao, China

**Keywords:** football participation, gender stereotypes, passion, psychological network analysis, the dualistic model of passion

## Abstract

**Introduction:**

Football serves as a premier platform for youth development, yet gender-based participation gaps remain a persistent challenge within the global sporting landscape. Drawing on Ecological Systems Theory as a conceptual framework, this study utilized psychological network analysis to examine selected psychosocial factors related to football participation among primary school students.

**Methods:**

The sample comprised primary school students (*M* = 9.322, *SD* = 0.677, age range = 8 to 13 years). We employed psychological network analysis with the Network Comparison Test for network estimation and gender comparisons.

**Results:**

Harmonious passion emerged as the most central node across both construct and item level networks, showing strong connections to parental support and behavioral engagement. While the Network Comparison Test confirmed a largely invariant psychosocial architecture across genders, exploratory analysis revealed localized descriptive variations: obsessive passion items occupied more central positions in girls’ networks, whereas gender stereotypes were more prominent in boys’ networks.

**Discussion:**

These findings indicate that harmonious passion occupies a central position in the network structure of children’s football participation. This study underscores the need for interventions that prioritize autonomous motivation while addressing the subtle, gender-specific nuances within children’s sporting environments.

## Introduction

Children’s moderate engagement in sports represents a critical developmental context that shapes physical health, psychological well-being, and social competencies (Brown et al., 2020; Greule et al., 2024). Football offers a unique platform for children to develop these attributes through structured play and social interaction as the world’s most popular team sport (Mao et al., 2024). Despite global efforts to promote youth football, participation gains have been uneven, with girls consistently underrepresented (Zeng and He, 2024). Existing research has typically examined isolated predictors such as parental support or stereotypes, leaving it unclear how these factors interact as a system to sustain or reduce gender disparities.

We employed psychological network analysis to examine the interconnections among key psychosocial factors in children’s football participation. This systemic approach identifies centrally positioned nodes that may be important targets for interventions. By visualizing the interconnections among these factors and identifying gender specific patterns, this study highlights centrally positioned nodes for consideration in intervention design.

### The benefits and challenges of children’s football participation

Regular engagement in sports like football can substantially benefit children for their cardiovascular health, motor skill development, and weight management (Alesi et al., 2015; Oja et al., 2015). Additionally, football participation promotes psychological resilience and emotional well-being by fostering positive peer interactions and a sense of accomplishment (Brett et al., 2019; Buecker et al., 2021). Socially, team sports foster cooperation, communication skills, and leadership abilities that transfer to academic and later professional settings (Friedrich and Mason, 2017). Some countries have adopted football as an important intervention for populations (Magee et al., 2015; Pringle et al., 2014).

Despite these established benefits, children and adolescent football engagement faces challenge in participation in many countries. For example, a systematized review highlights that dropout often results from overlapping factors including low self-perceived competence, lack of parental or coach support (Schlesinger et al., 2018). Similar situations have been observed in China (Qian et al., 2017), despite efforts by the Chinese government to promote football like campus football initiatives and the establishment of specialized football academies in school. These campaigns aim to increase student football participation (Jia et al., 2022). However, as considerable room for improvement remains in children and adolescent engagement, it is imperative to explore influencing factors and implement targeted interventions to enhance both participation and physical health.

### An ecological perspective on children’s sports engagement

Ecological systems theory emphasizes that human behavior develops from interactions between the individual and multi-level environmental systems (Bronfenbrenner and Ceci, 1994). In sports, this framework examines children’s behavior within nested levels: the microsystem (family, peers), mesosystem (home school interaction), exosystem (community resources), macrosystem (sociocultural context), and chronosystem (life course) (Bronfenbrenner, 1979). Within the microsystem, parental support is associated with positive developmental outcomes and sustained physical activity participation (Holt et al., 2008). However, excessive or controlling support may reinforce gender biases toward male dominated sports like football. Concurrently, gender stereotypes from the macrosystem can shape children’s motivation and behavior. Guided by this theory, the present study uses network analysis to examine how microsystem factors (parental support) and macrosystem factors (gender stereotypes) jointly influence football participation through distinct motivational pathways.

### Parental support and gender stereotypes as key ecological factors

Parental influence on children’s sport behavior is well documented. Between ages 5 and 12, parents are particularly influential (Beets et al., 2010), and a large body of evidence supports a significant connection between parental and child physical activity (Anderson et al., 2003; Rhodes et al., 2020). In football, supportive parental behaviors such as encouragement positively affect participation (Holt et al., 2008). Yet children’s perceptions of parental support may vary by gender, with girls potentially receiving less support for male typed sports (Hong et al., 2020).

Gender stereotypes begin early and can adversely affect children’s cognition and behavior. In sports, girls exposed to negative stereotypes may experience diminished performance (Hong et al., 2020), creating a self-perpetuating cycle (Eagly and Koenig, 2021). In football specifically, stereotypes impair female performance (Cardozo et al., 2021; Chalabaev et al., 2008; Mousavi and Soltanifar, 2025), but research on how stereotypes affect participation remains limited. Stereotypes may influence activity choice and withdrawal through mechanisms like self-perception (Chalabaev et al., 2013). Examining how football related stereotypes affect both boys and girls can inform interventions to improve children’s engagement.

### The dualistic model of passion

Passion is a strong inclination toward an activity that individuals love and value, driving them to invest time and energy (Vallerand et al., 2003). The dualistic model distinguishes harmonious passion, where individuals freely choose to integrate the activity into their lives, from obsessive passion, where individuals feel controlled by their urge to engage (Vallerand et al., 2008). Harmonious passion predicts positive outcomes such as life satisfaction, while obsessive passion is linked to burnout and anxiety (Curran et al., 2015). Prior research has focused primarily on athletes (Schellenberg and Lötscher, 2024), with limited attention to school aged children. Because passion for physical activity is cultivated through early positive experiences, examining passion in children’s football is important for promoting sustainable participation (Lafrenière et al., 2013).

### From isolated factors to an interconnected system

From an ecological systems perspective, children’s behaviors are influenced by parental support, often mediated through children’s motivation, beliefs, and intentions that these factors do not operate in isolation (Bronfenbrenner, 1979). Instead, they interact across different levels of a child’s environment, from the immediate family context to broader cultural beliefs, to jointly shape behavior. Parental support within the microsystem may influence participation by nurturing a child’s passion. Concurrently, gender stereotypes from the macrosystem could potentially diminish motivation or foster a more controlled, obsessive form of passion, thereby hindering engagement (Chalabaev et al., 2013). While these individual pathways are supported theoretically, few studies have empirically examined how parental support, gender stereotypes, and dualistic passion interrelate simultaneously to form a coherent psychosocial system that drives football participation.

To address this gap and directly model the interdependent relationships suggested by ecological theory, the present study employs psychological network analysis. This approach is uniquely suited to visualizing the architecture of the selected psychosocial factors and to testing gender differences in their interconnections. Specifically, it allows us to identify which factors are most central within the network, how they interconnect, and whether these patterns differ between boys and girls, thereby moving from examining isolated factors to understanding their synergistic effects.

### Psychological network analysis as a systemic approach

This study employs psychological network analysis to model the complex interdependencies among parental support, gender stereotypes, dualistic passion, and football participation. Psychological network analysis represents a shift from traditional approaches that often examine variables in isolation or assume predefined causal pathways (Epskamp and Fried, 2018). It also conceptualizes psychological phenomena as systems of interacting components.

In a network model, each variable is represented as a node. The estimated conditional dependencies between them, after accounting for all other variables in the system, are represented as connecting lines called edges. This methodology offers distinct advantages for studying complex systems like sports participation for children. Firstly, network visualization translates intricate relationship patterns into an interpretable map, revealing structures that summary statistics might hide (Epskamp et al., 2018). Secondly, centrality indices identify the nodes that exert the strongest influence on the overall network, highlighting potential targets for intervention (Hevey, 2018). Thirdly, community detection algorithms can uncover natural groupings of variables, which may either validate theoretical constructs or reveal unexpected associations (Tabassum et al., 2018). Finally, network comparison tests (NCT) allow for rigorous examination of differences in the overall structure of networks between groups, going beyond simple comparisons of group means on individual variables (Burger et al., 2023).

Thus, psychological network analysis provides a methodological framework that is conceptually aligned with ecological systems theory. It allows us to operationalize the theory’s core premise of interconnectedness by examining the specific pathways through which the selected psychosocial factors collectively relate to children’s football participation.

### The present study

This study employed psychological network analysis to examine the interconnections among parental support, gender stereotypes, passion, and football participation in Chinese children, with a specific focus on investigating potential gender differences. We estimate both construct-level and item-level networks to identify central drivers and gender-specific pathways.

## Methods

### Sample

This study recruited a total of 570 questionnaires and after excluding blank and invalid questionnaires and data (Lelkes et al., 2012), the final sample included in the study was 416 participants (*M* age = 9.322, *SD* = 0.677, age range = 8–13 years. Male = 213, female = 203). Regarding sample size adequacy, simulation studies for sparse networks with up to 30 nodes recommend sample sizes between 250 and 500 for stable strength centrality estimates (Constantin et al., 2023). Our sample of 416 falls within this range, which is supported by the correlation stability coefficients for strength (CS = 0.75) and closeness (CS = 0.67) at the item level. In contrast, betweenness centrality was insufficiently stable (CS = 0.05) and was therefore not interpreted. Future studies with larger samples are needed to confirm the descriptive bridging patterns observed in the gender comparison.

### Procedures

This study was approved by the Ethics Committee (2024LCLL-46). Participants were recruited using a convenience sampling approach from a single primary school in Guangzhou between March and April 2024. Following the acquisition of parental consent, students completed paper-based questionnaires in their classrooms. All participants received a small gift as a token of appreciation after questionnaire submission. All information provided by students was kept confidential.

### Measures

#### Parental support (PS)

Parental support for sport was measured using the subscale of the Perceived Social Support Scale, specifically the family support dimension (Zimet et al., 1988). This scale comprises 4 items and uses a 5-point Likert scale (1 = Not agree at all, 5 = Very strongly agree). In the current study, the scale demonstrated excellent reliability (*α* = 0.907, *ω* = 0.910) and construct validity (AVE = 0.732, CR = 0.916).

#### Gender stereotypes (GS)

The Gender Stereotypes Scale was adapted from the Gender Stereotypes Scale in Mathematics (Tomasetto et al., 2015) by modifying the context to football. Given that football is often perceived as a male-dominated sport (Koivula, 1999), references such as “mathematics” were replaced with “football” and “language” with “dance.” Prior to data collection, the content validity of the adapted scale was evaluated by seven experts in sports science, statistics, and psychology. Each expert rated the content design, structural design, and overall design of the scale on a 5-point scale. In this study, the scale exhibited good reliability (*α* = 0.896, *ω* = 0.897) and construct validity (AVE = 0.683, CR = 0.896).

#### Passion

The Passion Scale used was the Chinese version (Zhao et al., 2015), adapted to focus on football by replacing “academic activities” with “football.” The scale consists of 17 items, with two 6-item subscales measuring harmonious passion (HP) and obsessive passion (OP). The scale uses a 7-point Likert scale (1 = “Not agree at all” to 7 = “Very strongly agree”). In the current study, both subscales demonstrated excellent reliability and construct validity: harmonious passion (α = 0.961, ω = 0.961, AVE = 0.806, CR = 0.961) and obsessive passion (α = 0.946, ω = 0.946, AVE = 0.746, CR = 0.946).

#### Football participation (FP)

Football participation was assessed using two self-report items relating to the past 7 days. The first item asked about the number of days the child participated in football activities (excluding school-arranged football). The second item asked about the average number of hours per day spent on football participation. Because the two items were measured on different units (days vs. hours), their raw scores were converted to z-scores before averaging to create a composite participation score. Extreme values (z < −3.29 or z > +3.29) were identified and excluded prior to analysis. This composite score served as the FP node in the network.

#### Demographic variables

We collected basic demographic information from participants, including their self-reported gender (male = 1, female = 2), age, height, and weight.

#### Attention check

To ensure data quality and identify inattentive responding, we included one instructed response check item at the end of the questionnaire (Oppenheimer et al., 2009). The item asked, “Are there 13 months in a year?” Participants who answered incorrectly were flagged for exclusion. Of the 570 initial participants, 30 failed this attention check and were excluded from further analysis.

All measures used in this study, including the full wording of every item in both Chinese and English, are provided in Supplementary Table S1.

### Data analysis

We used R 4.3.1 to conduct all analyses including data quality, network estimation, and gender comparisons. Preliminary analyses included descriptive statistics and Spearman correlations. We assessed scale reliability using Cronbach’s alpha and McDonald’s omega coefficients via the psych package. Confirmatory factor analysis using the *lavaan* package (Rosseel, 2012) verified measurement models and the robust maximum likelihood estimator accommodated potential non-normality.

Our main analyses employed network analysis to examine relationships among study variables. All network analyses were based on Pearson product moment correlations. Although the Likert scale items (5 point and 7 point) are ordinal, simulation studies that Pearson correlations are robust to violations of continuity when the number of response categories is five or more, and they produce negligible bias compared to polychoric correlations under most conditions (Rhemtulla et al., 2012). The two Football Participation items were originally continuous measures of frequency and duration. Therefore, we retained Pearson correlations for all variables to maintain consistency across the entire correlation matrix.

We estimated regularized partial correlation networks using the EBICglasso method (Foygel and Drton, 2010) with gamma set to 0.5, implemented in the *qgraph* package (Epskamp et al., 2012). We computed four centrality indices to identify important nodes: strength, closeness, betweenness, and expected influence. Clustering coefficients were calculated using four algorithms (Barrat et al., 2004). Network stability was evaluated using nonparametric bootstrapping via the *bootnet* package (Epskamp et al., 2018). We computed correlation stability (CS) coefficients, with values above 0.50 indicating adequate stability for interpretation. Gender differences were tested using the Network Comparison Test (NCT) with 1,000 permutations (Van Borkulo et al., 2023). This approach tests global strength invariance, edge weight invariance, and centrality differences between groups.

All analysis scripts are publicly available at Open Science Framework (doi: 10.17605/OSF.IO/RCXGQ).

## Results

### Construct validity

A five-factor confirmatory factor model including all constructs (Gender Stereotypes, Harmonious Passion, Obsessive Passion, Parental Support, and the two-item Football Participation factor) was tested. The two-item factor was identified by fixing its variance to 1 and freely estimating the factor loadings, with the model automatically imposing a fixed loading of 1 for the first indicator (the default in lavaan). The model fit was good: χ^2^(199) = 559.3; CFI = 0.955; TLI = 0.948; RMSEA = 0.067; SRMR = 0.041. All factor loadings were significant, supporting the construct validity of the scales.

### Descriptive statistics

All key constructs used Likert scales except football participation which was assessed via standardized self-reported items. Parental sport support had a mean of 3.96 (*SD* = 1.02). Gender stereotypes showed a mean of 3.28 (*SD* = 1.18). Harmonious passion had a mean of 4.50 (*SD* = 1.81) while obsessive passion had a mean of 3.55 (*SD* = 1.83). Football participation had a standardized mean of −0.05 (*SD* = 0.83).

Correlation analyses revealed that age was not significantly associated with any key construct (all *p* > 0.05). Gender was significantly negatively correlated with gender stereotypes (*r* = −0.170, *p* < 0.001), harmonious passion (*r* = −0.145, *p* = 0.003), obsessive passion (*r* = −0.115, *p* = 0.020) and football participation (*r* = −0.271, *p* < 0.001).

Harmonious passion was strongly positively correlated with obsessive passion (*r* = 0.738, *p* < 0.001). Both passion types showed significant positive correlations with football participation (harmonious passion: *r* = 0.548, *p* < 0.001; obsessive passion: *r* = 0.444, *p* < 0.001). Parental sport support was significantly positively associated with harmonious passion (*r* = 0.449, *p* < 0.001), obsessive passion (*r* = 0.273, *p* < 0.001) and football participation (*r* = 0.249, *p* < 0.001). Gender stereotypes had significant positive correlations with harmonious passion (*r* = 0.277, *p* < 0.001) and obsessive passion (*r* = 0.256, *p* < 0.001) as well as football participation (*r* = 0.133, *p* = 0.007).

**Figure 1:**
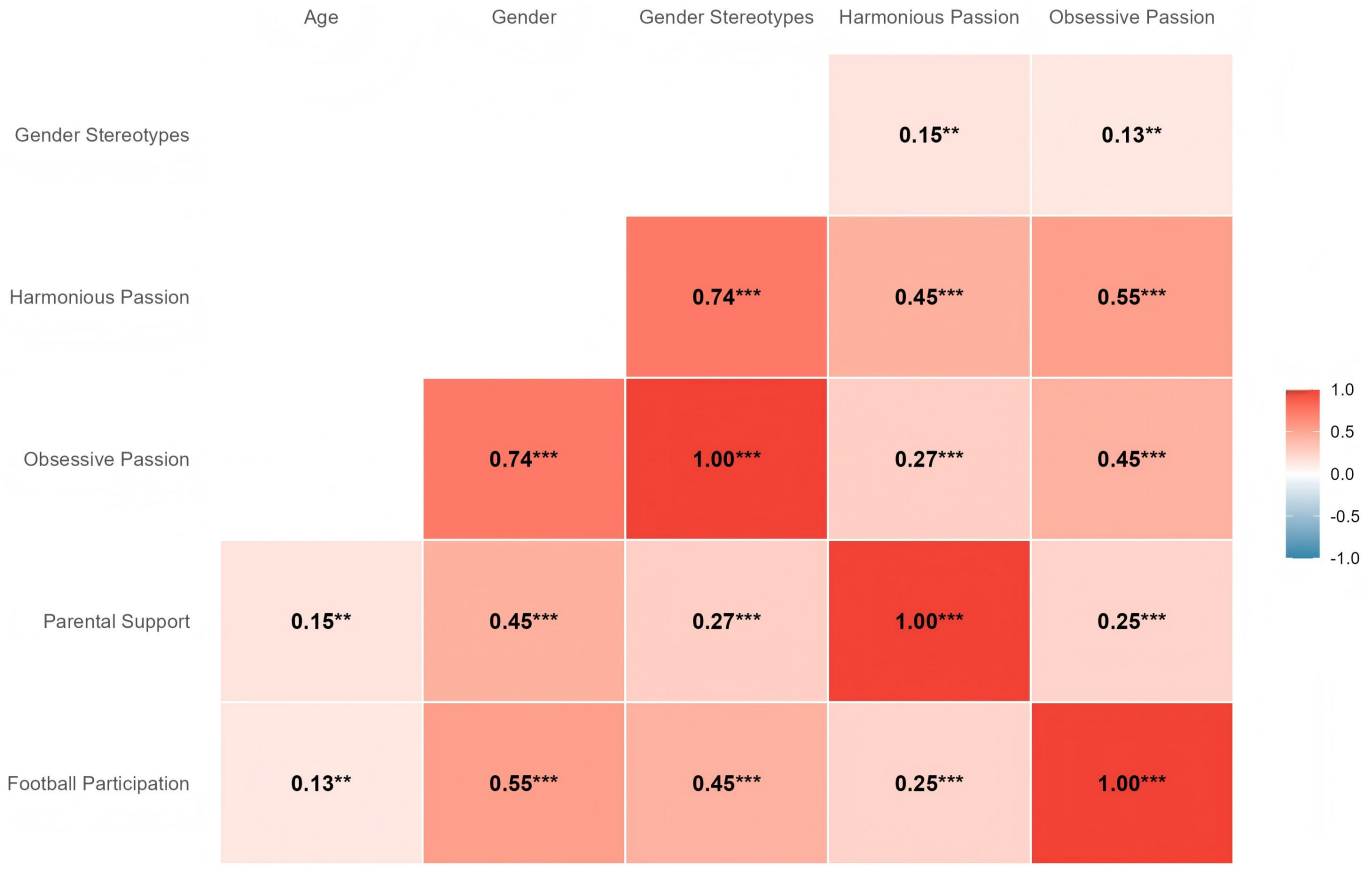
Correlation heatmap. ^*^p < 0.05, ^**^p < 0.01, ^***^p < 0.001.

Results of correlations are presented in Figure 1. These findings confirm the expected interrelationships among key constructs and provide a valid foundation for subsequent psychological network analysis.

### Common method bias tests

Common method bias was assessed using Harman’s single-factor test (Podsakoff et al., 2003). The first unrotated factor explained 45.4% of the total variance, slightly above the 40% threshold, but confirmatory factor analysis showed that a five-factor model (CFI = 0.947, RMSEA = 0.052) fit substantially better than a single-factor model (CFI = 0.611), suggesting that common method bias did not severely distort the results.

### Goldbricker redundancy analysis

Prior to network estimation, we assessed potential item redundancy using the goldbricker function from the *networktools* package with *p* = 0.05, method = “hittner2003,” threshold = 0.25, and corMin = 0.5 (Hittner et al., 2003). The analysis flagged several variable pairs as potentially redundant, all of which were within the same theoretical construct (e.g., GS1–GS2, HP1–HP10, OP2–OP7, PS1–PS2). Critically, no cross-construct pair between Harmonious Passion (HP) and Obsessive Passion (OP) was flagged as redundant. The proportion of significantly different correlations between HP and OP items with other variables exceeded the threshold of 0.25, indicating that despite their moderate to high zero-order correlation (*r* = 0.738), HP and OP exhibited sufficiently distinct correlation patterns with other constructs in the dataset.

### Construct-level network analysis

**Figure 2:**
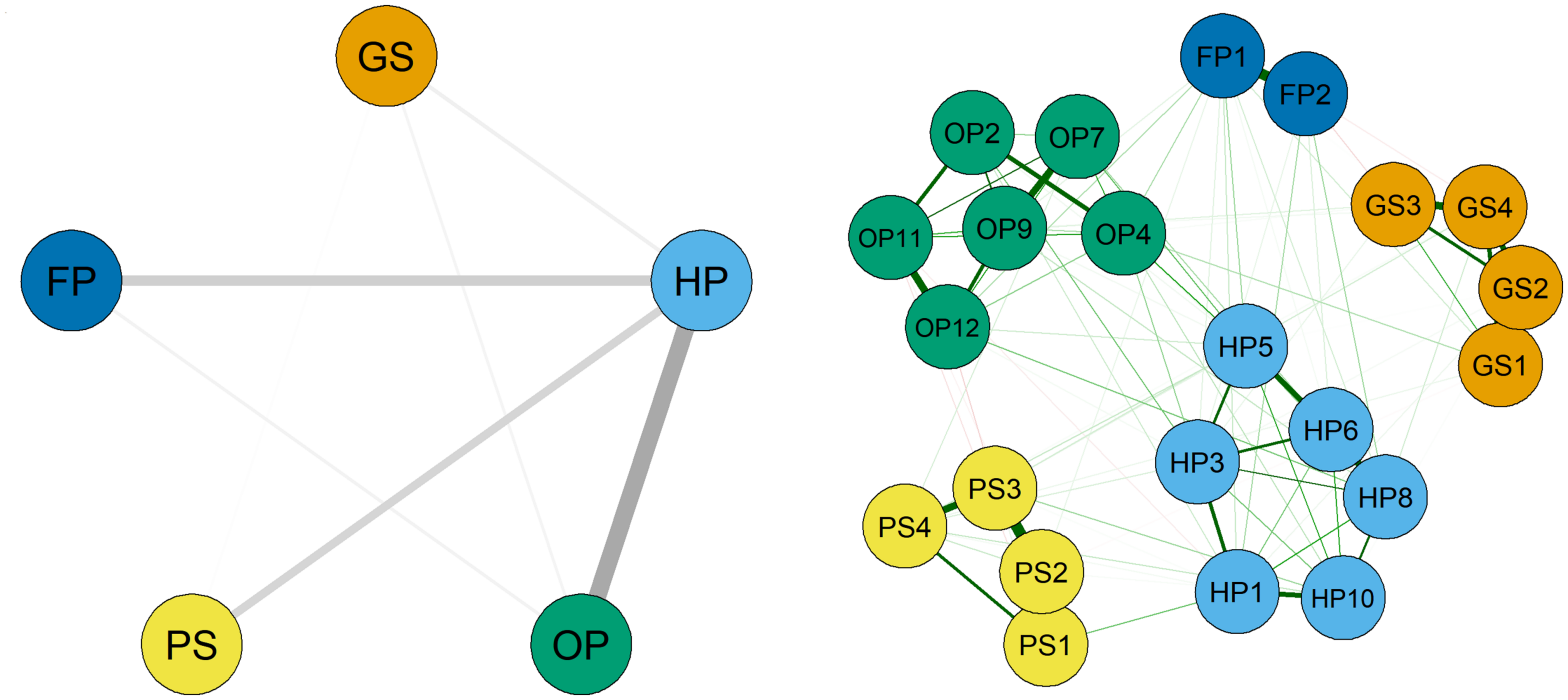
Construct- and item-level network of psychological variables. Node size represents strength centrality, edge thickness represents absolute edge weight. PS = Parental Support, HP = Harmonious Passion, OP = Obsessive Passion, GS = Gender Stereotypes, FP = Football Participation.

**Figure 3:**
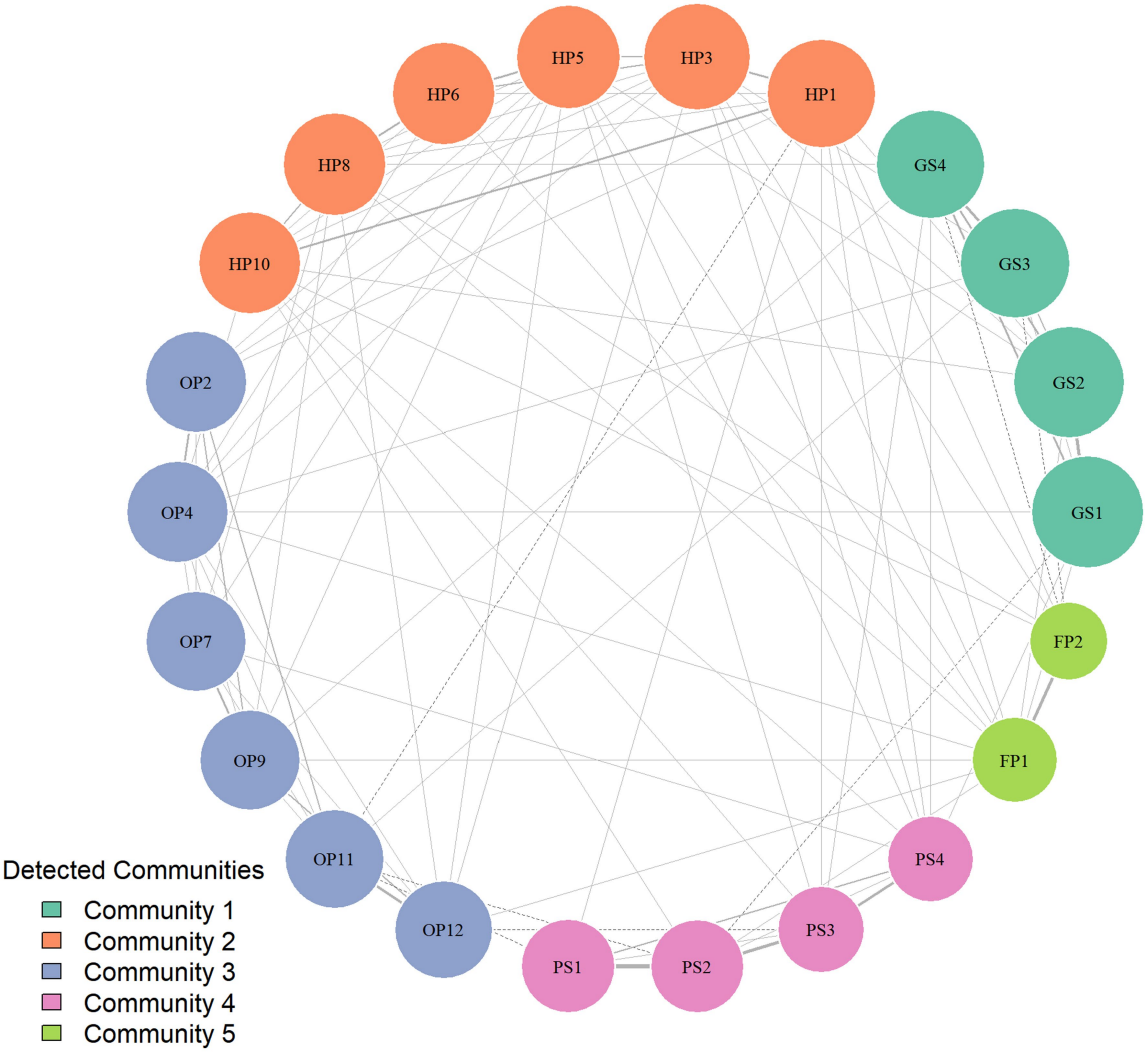
Community structure of item-level network. Nodes are colored by theoretical construct. Louvain algorithm recovered five communities perfectly aligned with the five constructs (ARI = 1.00, modularity = 0.171). PS = Parental Support, HP = Harmonious Passion, OP = Obsessive Passion, GS = Gender Stereotypes, FP = Football Participation.

Figure 2 presents the construct level network with five psychological nodes. EBICglasso regularization retained seven positive edges out of ten possible, resulting in a network density of 70 percent. Edge weights ranged from 0.016 to 0.581, with an average of 0.208 (*SD* = 0.200). The global clustering coefficient was 0.643, indicating moderate local connectivity among constructs. Community detection using the Louvain algorithm recovered five communities that perfectly matched the five theoretical constructs (adjusted Rand index = 1.00). However, the modularity was only 0.171 (Figure 3), which is below the commonly used threshold of 0.30 for clear community structure. This low modularity indicates that while the algorithm assigned items to groups consistent with theory, the connections between different constructs were relatively dense. This pattern is consistent with the high observed correlation between Harmonious Passion and Obsessive Passion (*r* = 0.738) and suggests substantial cross-construct connectivity rather than strong empirical separation.

Construct centrality analysis revealed Harmonious Passion (HP) as the most influential construct with strength centrality of 1.291, followed by Obsessive Passion (OP, 0.733), Football Participation (FP, 0.408), Parental Support (PS, 0.297), and Gender Stereotypes (0.189). HP also showed the highest betweenness centrality (12), indicating a bridging position in the network structure (Table 1).

**Table 1:** Results construct centrality.

Construct	Strength	Closeness	Betweenness	Expected Influence
Harmonious passion	1.291	0.056	12	1.291
Obsessive passion	0.733	0.043	0	0.733
Football participation.	0.408	0.037	0	0.408
Parental support	0.297	0.035	0	0.297
Gender stereotypes	0.189	0.021	0	0.189

Values represent standardized *z*-scores. Higher values indicate greater centrality.

Network stability assessment yielded CS coefficients of 0.75 for strength centrality, 0.75 for betweenness centrality, and 0.673 for closeness centrality. These values surpass established thresholds for acceptable stability, indicating that network parameters remained consistent across bootstrap resamples. The network structure therefore demonstrated both theoretical coherence and statistical robustness.

**Figure 4:**
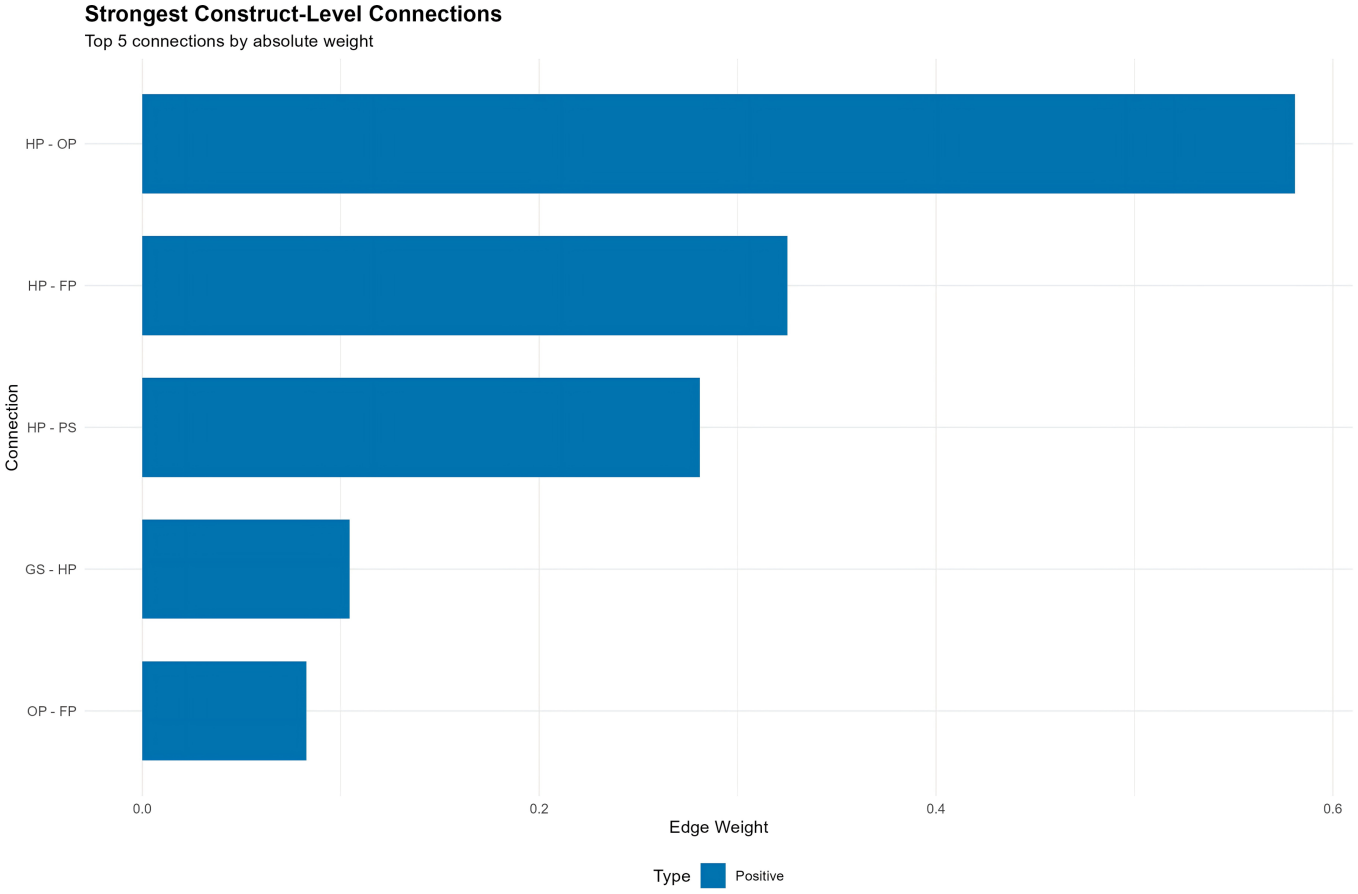
Construct-level network with edge weights. PS = Parental Support, HP = Harmonious Passion, OP = Obsessive Passion, GS = Gender Stereotypes, FP = Football Participation.

As shown in Figure 4, HP was most strongly connected to OP (weight = 0.581), followed by FP (0.325) and PS (0.281). This strong positive association between the two passion types suggests that harmonious and obsessive passion coexist as complementary motivational orientations in children rather than opposing forces. The robust connections of HP to both participation and parental support indicate that autonomous motivation is strongly associated with both social support and behavioral engagement. GS was the most peripheral node, with its only connection to HP (weight = 0.104). This pattern shows that gender stereotypes are not strongly directly connected to football participation but are connected to harmonious passion, suggesting an indirect associative pathway. This pattern suggests that interventions targeting stereotypes alone might need to also address motivational pathways, though causal testing is required.

### Item-level network analysis

The item-level network comprised 22 measurement items, with 100 of 231 possible edges retained (density = 43.3%). The average edge weight was 0.102 (*SD* = 0.125), approximately half the magnitude observed at the construct level. Edge weights ranged from −0.026 to 0.581, with 93 positive edges (93%) and seven negative edges (7%). The global clustering coefficient was 0.475, lower than that of the construct-level network, suggesting that individual items formed less clustered connectivity patterns than their aggregated constructs. Edge weight distribution and additional network diagnostics are provided in the Supplementary Figure S1.

**Figure 5:**
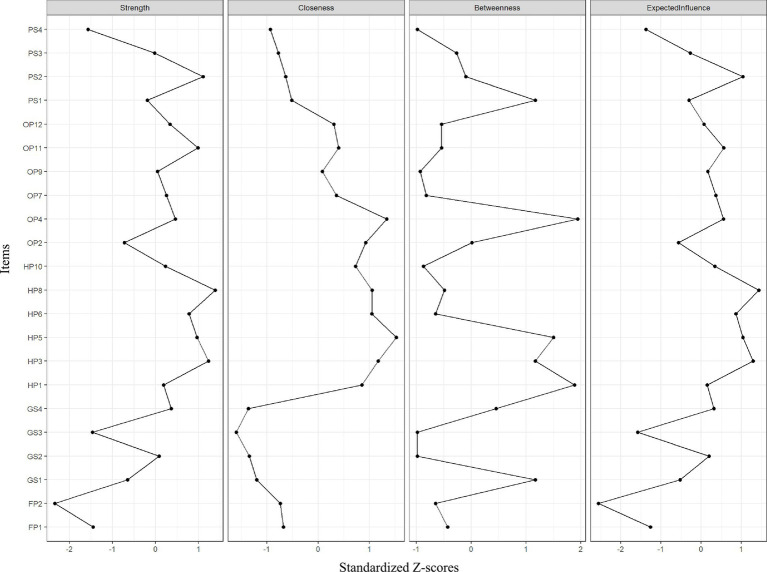
Item-level centrality plot (strength, closeness, and expected influence).

Centrality analysis revealed HP8 as the most influential item (strength = 1.129), followed by HP3 (1.108) and PS2 (1.091). This indicates that the most central items in the network reflect internal motivation (HP8: “My football activities are well integrated in my life.”) and perceived parental encouragement (PS2: “I get the emotional help and support I need from my parents regarding my sport participation.”), underscoring the interplay between autonomous drive and family support. Across constructs, Harmonious Passion items showed the highest average centrality (*M* = 1.050, *SD* = 0.067), whereas Football Participation items showed the lowest (*M* = 0.687, *SD* = 0.084). Parental Support items exhibited the greatest within-construct variability (*SD* = 0.148), suggesting that this construct captures more heterogeneous measurement content compared to others. HP1 (“My football activities are in harmony with the other activities in my life.”) connects harmonious passion to other constructs, while OP4 (“I have almost an obsessive feeling for football.”) links obsessive passion to the broader network. This suggests that both adaptive and less adaptive forms of passion can serve as connective nodes, but through different psychological content. In contrast, several items including GS2, GS3, and PS4 exhibited zero betweenness centrality, occupying more peripheral positions (see Table 2 and Figure 5).

**Table 2:** Results item centrality.

Item	Strength	Closeness	Betweenness	Expected influence
HP8	1.129	0.003	18	1.129
HP3	1.108	0.003	78	1.108
PS2	1.091	0.002	32	1.073
OP11	1.075	0.002	16	1.005
HP5	1.072	0.003	90	1.072
HP6	1.047	0.003	12	1.047
OP4	1.004	0.003	106	1.004
GS4	0.991	0.001	52	0.970
OP12	0.987	0.002	16	0.935
OP7	0.977	0.002	6	0.977
HP10	0.974	0.002	4	0.974
HP1	0.968	0.002	104	0.947
GS2	0.953	0.001	0	0.953
OP9	0.949	0.002	2	0.949
PS3	0.939	0.002	26	0.887
PS1	0.917	0.002	78	0.883
GS1	0.855	0.002	78	0.851
OP2	0.845	0.002	36	0.845
FP1	0.746	0.002	20	0.746
GS3	0.744	0.001	0	0.701
PS4	0.730	0.002	0	0.730
FP2	0.627	0.002	12	0.563

Values represent standardized *z*-scores. Higher values indicate greater centrality. Due to low stability (CS = 0.05), betweenness centrality at the item level is reported for transparency but not interpreted in the main text. PS = Parental Support, HP = Harmonious Passion, OP = Obsessive Passion, GS = Gender Stereotypes, FP = Football Participation.

**Figure 6:**
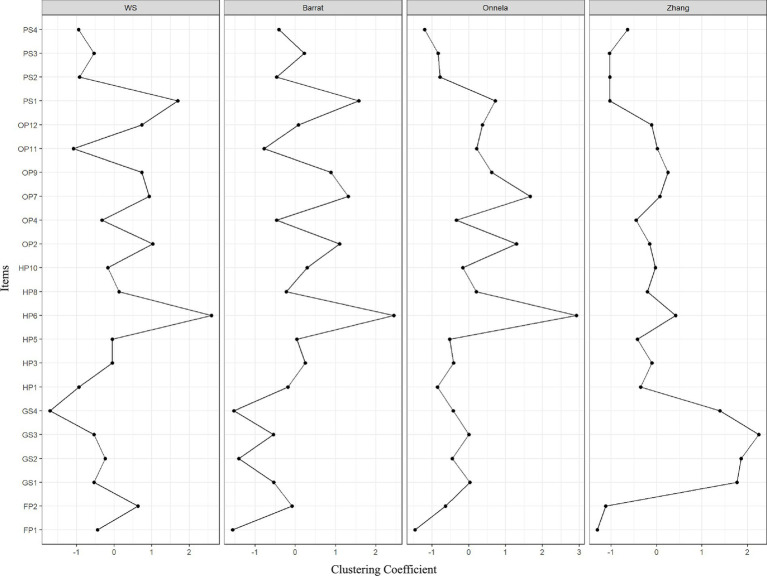
Item-level clustering plot (WS, Barrat, Onnela, Zhang).

Clustering analysis examined local connectivity patterns using four algorithms (Table 3 and Figure 6). Watts-Strogatz unweighted coefficients ranged from 0.286 (GS4) to 0.810 (HP6), indicating varying levels of local cohesion. Weighted clustering coefficients followed a similar pattern. HP6 (“My football activities allow me to live a variety of experiences.”) is embedded in a dense local neighborhood of other harmonious passion items, reflecting the coherent internal structure of the HP construct. In contrast, FP1 [“During the past 7 days, how many days did you participate in football activities (excluding school-arranged football activities)”] is only loosely connected to other items, suggesting that participation frequency is not strongly aligned with other psychological constructs in the network. At the item level, harmonious passion (*M* = 0.287, *SD* = 0.053) and obsessive passion items (*M* = 0.299, *SD* = 0.054) exhibited higher mean Barrat-weighted clustering than gender stereotypes (*M* = 0.216, *SD* = 0.032) and football participation items (*M* = 0.227, *SD* = 0.064). These patterns suggest that passion-related items form more tightly interconnected local neighborhoods within the network.

**Table 3:** Clustering coefficients of items.

Node	WS unweighted	Barrat weighted	Onnela weighted	Zhang weighted
GS1	0.429	0.244	0.169	0.223
GS2	0.464	0.192	0.126	0.193
GS3	0.429	0.244	0.167	0.219
GS4	0.286	0.184	0.207	0.270
HP1	0.379	0.266	0.124	0.192
HP3	0.487	0.292	0.122	0.169
HP5	0.487	0.279	0.116	0.162
HP6	0.810	0.426	0.193	0.245
HP8	0.509	0.263	0.152	0.188
HP10	0.473	0.295	0.142	0.192
OP2	0.619	0.344	0.176	0.228
OP4	0.455	0.249	0.136	0.170
OP7	0.607	0.357	0.197	0.233
OP9	0.583	0.331	0.153	0.198
OP11	0.361	0.230	0.215	0.268
OP12	0.583	0.282	0.141	0.174
PS1	0.700	0.373	0.132	0.295
PS2	0.381	0.249	0.129	0.286
PS3	0.429	0.290	0.111	0.251
PS4	0.378	0.252	0.098	0.197
FP1	0.439	0.182	0.067	0.118
FP2	0.571	0.272	0.093	0.172

PS = Parental Support, HP = Harmonious Passion, OP = Obsessive Passion, GS = Gender Stereotypes, FP = Football Participation.

Community detection using the Louvain algorithm recovered five communities that perfectly matched the five theoretical constructs (adjusted Rand index = 1.00), with a modularity of 0.171 (Figure 3). This perfect correspondence provides empirical evidence for the internal structure validity of the scales.

For the item-level network, case-dropping bootstrap indicated adequate stability for strength (CS = 0.75) and closeness (CS = 0.67). In contrast, betweenness centrality was insufficiently stable (CS = 0.05). This is consistent with prior work showing that betweenness centrality requires larger sample sizes to stabilize, particularly in networks with more than 20 nodes (Borsboom et al., 2021; Epskamp et al., 2018). Accordingly, betweenness centrality at the item level was not interpreted in this study.

### Gender differences in psychology network analysis

**Figure 7:**
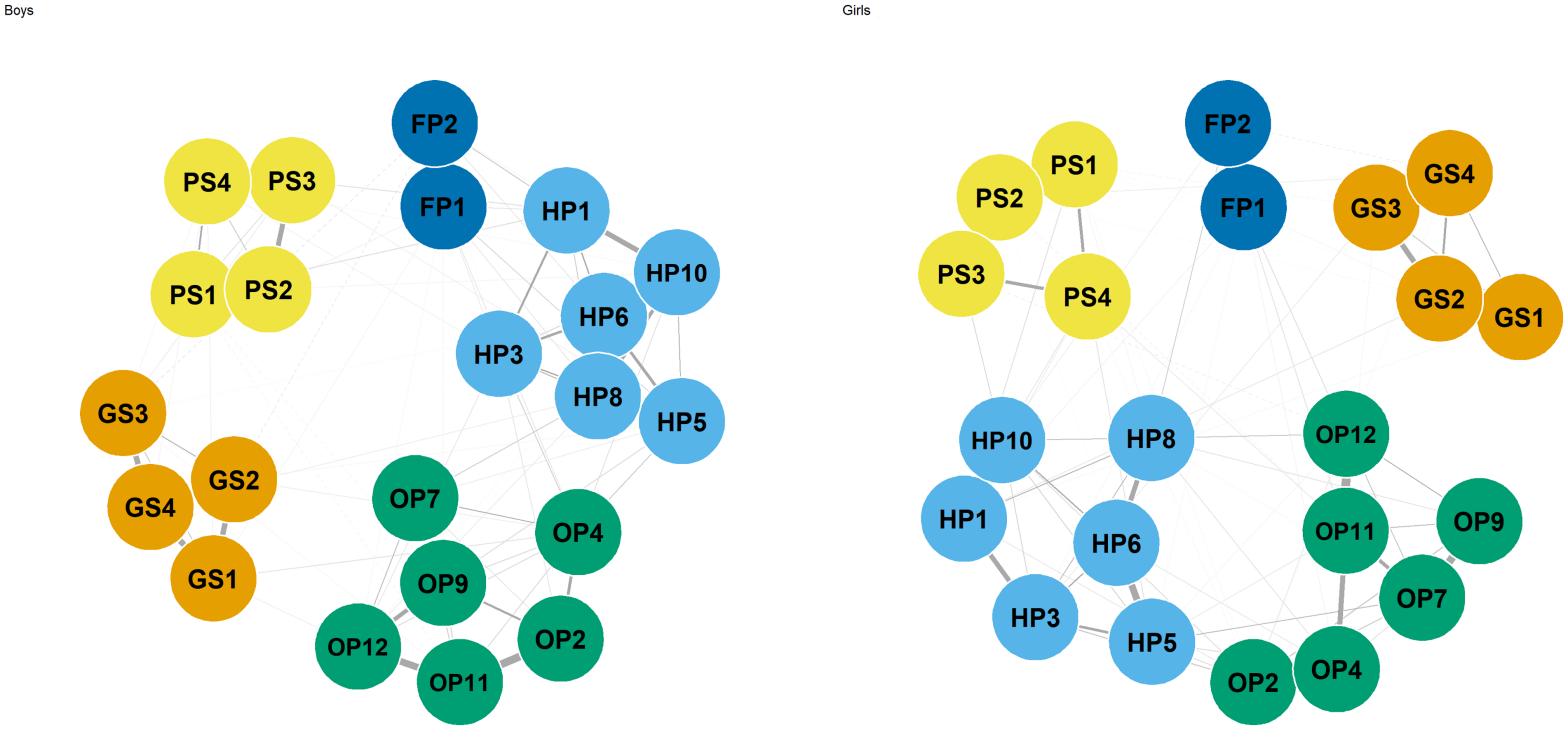
Network structures by gender. PS = Parental Support, HP = Harmonious Passion, OP = Obsessive Passion, GS = Gender Stereotypes, FP = Football Participation. Node size represents strength centrality, edge thickness represents absolute edge weight.

Separate networks were estimated for boys (*n* = 213) and girls (*n* = 203). The boys’ network had 90 edges (density = 38.96%), while the girls’ network had 83 edges (density = 35.93%). The network comparison test revealed no significant difference in global network strength between genders (*p* = 0.947). The test for edge weight invariance was also not significant (*p* = 0.052) (Figure 7).

Centrality analysis revealed descriptive differences in node centrality across genders, though these differences were not statistically tested. Harmonious passion items showed high centrality in both groups. Obsessive passion items OP7 and OP11 had higher centrality in girls’ networks (descriptive differences of 0.236 and 0.210), and gender stereotype item GS4 had higher centrality in boys’ networks (descriptive difference of 0.210). Within construct connectivity for football participation items was descriptively stronger in girls (mean edge weight = 0.482) than in boys (0.403). These exploratory observations should be interpreted with caution and are hypothesis generating only (Table 4).

**Table 4:** Items with largest centrality differences between genders.

Item	Construct	Boys strength	Girls strength	Strength difference	Strength difference direction
OP7	Obsessive Passion	0.857	1.093	0.236	Higher in Girls
GS3	Gender Stereotypes	0.672	0.885	0.213	Higher in Girls
OP11	Obsessive Passion	0.979	1.189	0.210	Higher in Girls
GS4	Gender Stereotypes	1.072	0.862	0.210	Higher in Boys
PS4	Parental Support	0.841	0.662	0.180	Higher in Boys

Strength values represent standardized z-scores. Positive differences indicate higher centrality in boys. Only the top 5 items with largest absolute differences are shown.

**Figure 8:**
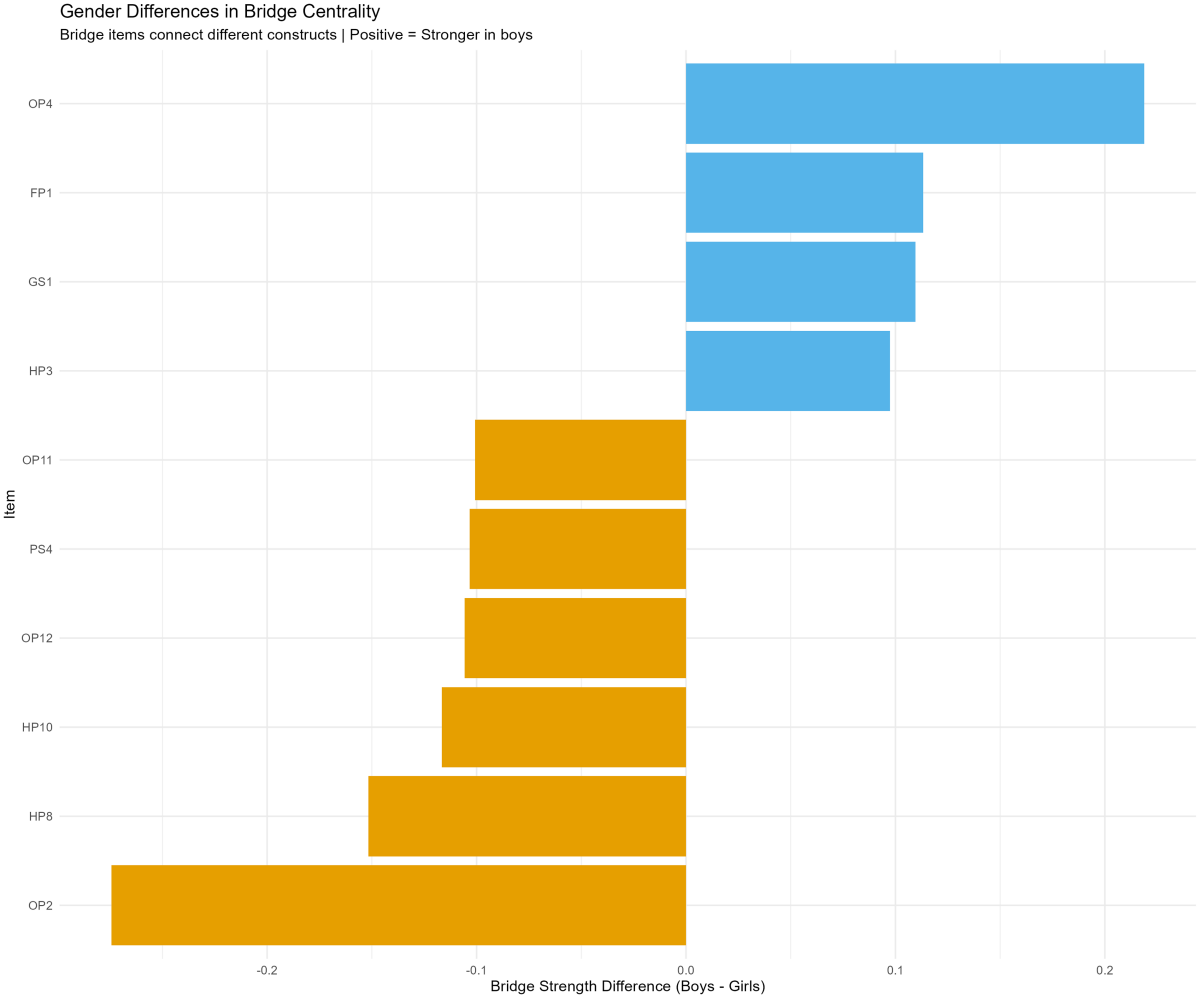
Gender differences in bridge centrality. PS = Parental Support, HP = Harmonious Passion, OP = Obsessive Passion, GS = Gender Stereotypes, FP = Football Participation.

At the item level, bridge centrality analysis (Table 5 and Figure 8) showed that the highest bridge strength values were similar across genders. In boys’ networks, obsessive passion item OP4 had a bridge strength of 0.411. In girls’ networks, harmonious passion item HP5 had a bridge strength of 0.410. These values are nearly identical, indicating no meaningful gender difference in bridge centrality.

**Table 5:** Top bridge items by gender group.

Rank	Boys	Bridge strength	Girls	Bridge strength
1	OP4 (Obsessive Passion)	0.411	HP5 (Harmonious Passion)	0.410
2	FP1 (Football Participation)	0.325	HP8 (Harmonious Passion)	0.349
3	HP5 (Harmonious Passion)	0.321	OP2 (Obsessive Passion)	0.326

Bridge strength values represent the item’s importance in connecting different theoretical constructs within the network.

Taken together, the overall network architecture was largely invariant across genders. Within this shared structure, descriptive variations emerging obsessive passion items were more central in girls, gender stereotypes more central in boys, and harmonious passion served as the primary bridge only in girls. These patterns suggest that gender differences in children’s football participation are not captured by global network properties but rather by the differential roles of specific cognitive and affective components.

## Discussion

The present study examined the interconnections among selected psychosocial factors in children’s football participation using network analysis. The results showed harmonious passion was the most central node in both construct and item level networks, indicating that autonomous motivation occupies a central position rather than a peripheral one. Moreover, while boys and girls exhibited similar global network structures, consistent gender differences emerged at the level of specific nodes and pathways, particularly in obsessive passion, gender stereotypes, and bridging patterns. These findings advance the dualistic model of passion and offer a network-informed perspective on gender disparities in children’s sports engagement.

### Harmonious passion as the central driver in children’s football networks

The present study identified harmonious passion as the most influential node in both the construct-level and item-level networks. The centrality of harmonious passion across both measurement levels extends prior work on the dualistic model of passion (DMP).

The strong positive association between harmonious and obsessive passion indicates that these two motivational orientations coexist in children rather than oppose one another. This finding aligns with the DMP which conceptualizes harmonious and obsessive passion as distinct but correlated constructs (Vallerand et al., 2003, 2024). Prior meta-analytic work has similarly documented their positive correlation and divergent outcomes (Curran et al., 2015). The present study extends this literature by demonstrating that the two passions are not merely correlated but structurally intertwined within a broader motivational network. Harmonious passion does not suppress obsessive passion but instead may channel obsessive intensity toward sustained engagement.

Within the microsystem of the family, harmonious passion was strongly connected to both parental support and football participation, placing it in a pathway between these two constructs. This finding moves beyond variable centered accounts that treat support and passion as independent predictors by showing that harmonious passion is centrally positioned in the network rather than peripheral (Verner-Filion et al., 2017). While prior work showed that parental autonomy support fosters harmonious passion (Mageau et al., 2009), the present study demonstrates that harmonious passion is centrally connected within the motivational system and is strongly associated with participation (Lopes and Vallerand, 2020). The weaker connection involving obsessive passion is consistent with evidence that controlling parental behaviors are less strongly associated with sustained engagement (Kovácsik et al., 2021).

Gender stereotypes originated from the macrosystem (Bronfenbrenner, 1979) and were the most peripherally connected node in the network, with their sole link to harmonious passion. The peripheral position of gender stereotypes indicates that macrosystem-level cultural beliefs influence athletic participation primarily through their indirect effects on proximal motivational processes (Chalabaev et al., 2013). The weak embedding of stereotypes reflects their distal status within the psychosocial ecosystem: as macrosystem beliefs, they must be mediated by proximal motivational factors to influence children’s football participation. These findings illustrate how microsystem support and macrosystem beliefs are both connected to harmonious passion, which in turn shows strong associations with football participation.

Thus, harmonious passion occupied the structural core of children’s football motivation. It coexisted with obsessive passion, translated microsystem support into sustained participation, and served as the sole conduit through which macrosystem stereotypes exerted indirect influence. Our findings position harmonious passion as a centrally connected node rather than a peripheral one, highlighting its strong associations with both social support and behavioral engagement.

### Gender differences manifest in specific nodes and pathways, not global structure

The network comparison test revealed no significant gender differences in global network strength or edge weight invariance, indicating that boys and girls share a similar overall psychosocial architecture. The following descriptive patterns, which were not statistically confirmed, are presented as exploratory observations to generate hypotheses for future research, rather than as evidence of differential socialization.

Descriptively, parental support items appeared more central in boys’ networks, while the two football participation items showed stronger inter connection among girls. One speculative interpretation, pending statistical confirmation, is that boys and girls may integrate parental support differently. However, because the NCT did not detect significant group differences, this interpretation is not empirically supported and requires direct testing in future studies.

Gender stereotype items showed higher centrality in boys’ networks than in girls’ networks. One speculative interpretation, which is not statistically supported by the NCT results, is that stereotypes may play a more prominent associative role in boys’ motivational networks. An alternative hypothesis is that girls’ networks show stronger connections between harmonious passion and participation, potentially moderating the influence of stereotypes. However, because the NCT did not detect significant group differences, these speculations should be treated as hypothesis generating only, not as empirical conclusions.

Obsessive passion items appeared more central in girls’ networks, and they did not show high bridge centrality. In boys’ networks, obsessive passion descriptively showed both high centrality and high bridge centrality. These descriptive patterns were not statistically compared. The observed descriptive variations raise the possibility that certain constructs, such as obsessive passion, may serve different functional roles across genders, a hypothesis that warrants further investigation with larger samples. Descriptively, obsessive passion items appeared more central in girls’ networks. One hypothesis is that this reflects internalized performance pressure, but this interpretation requires direct testing (Gentile et al., 2018). For boys, obsessive passion descriptively showed higher centrality, which might be hypothesized as a motivational amplifier. Descriptively, harmonious passion appeared as a bridge in girls’ networks but not in boys’ networks. One hypothesis is that this pattern reflects more autonomously organized engagement among girls, but this interpretation is not statistically confirmed by the NCT results.

The descriptive patterns observed in this study lead to speculative hypotheses about gender specific trends, such as stereotypes being more centrally connected in boys’ networks and harmonious passion being more centrally connected in girls’ networks. However, because the NCT did not detect significant group differences, these hypotheses are not empirically supported. Future research with larger samples and confirmatory methods is needed to test whether such patterns are replicated and whether they reflect meaningful gender differences. Interventions should be informed by confirmed evidence rather than exploratory patterns.

### Practical implications

The present findings offer three implications for parents and educators concerned with children’s football participation.

Harmonious passion bridged parental support and participation, indicating that autonomous motivation organizes rather than merely accompanying involvement. Parents should therefore help children internalize football as personally meaningful rather than rely on external rewards. Autonomy supportive strategies including providing choice, acknowledging feelings and minimizing controlling language foster harmonious passion and should be integrated into coach education and parent guidance (Burke et al., 2024).

Gender differences emerged not in global structure but in specific pathways. Boys’ networks emphasized gender stereotypes and obsessive passion, whereas girls’ networks emphasized harmonious integration. Reducing stereotype threat through positive role models, growth mindset feedback, and identity-safe environments can transform girls’ obsessive engagement into more harmonious forms (Chalabaev et al., 2013; Walton and Cohen, 2003). For boys, critical reflection on masculine norms and exposure to counter-stereotypical role models should be incorporated into physical education to target the content and function of gender stereotypes (Heidrich et al., 2025).

Coaches and physical education teachers play a crucial role in cultivating children’s harmonious passion. Adapting football activities to children’s developmental levels by simplifying rules and incorporating playful elements can enhance enjoyment and autonomous motivation (Elliott et al., 2020). Such pedagogical approaches help children internalize football as a source of personal meaning rather than external pressure, thereby fostering sustainable participation.

### Limitations and future studies

Several limitations should be acknowledged. Firstly, the cross-sectional design precludes causal inferences about the direction of relationships among parental support, passion, dualistic passions, and football participation. Although network analysis captures the contemporaneous structure of these constructs, it does not test whether harmonious passion precedes participation or vice versa. Future studies should employ longitudinal network models to examine how the psychosocial ecosystem evolves over time and whether the centrality of harmonious passion predicts sustained engagement.

Additionally, the sample was recruited from a single elementary school, which limits the generalizability of the findings. Cultural norms regarding gender, sport participation, and parental involvement may differ across regions and educational contexts. Future research should replicate the current network structure and gender differences in diverse geographical and cultural settings to establish the robustness of the observed patterns.

Moreover, this study relied on self-report questionnaires completed by children, which may introduce common method bias. Although the Harman single-factor test was conducted, its low sensitivity and the fact that the first factor explained 45.4% of variance (above the 40% threshold) mean that some degree of common method bias cannot be ruled out. Future research should employ multiple informants or objective measures of football participation, as well as use statistical controls such as an unmeasured latent method factor or a marker variable, to reduce the potential impact of method bias (Podsakoff et al., 2012).

Finally, betweenness centrality at the item level was insufficiently stable (CS = 0.05) and could not be interpreted. This instability is common in networks with more than 20 nodes and sample sizes below 500 (Epskamp et al., 2018). Although strength centrality proved robust, the limited reliability of betweenness constrains the interpretation of bridging roles at the item level. Future studies with larger samples should re-examine whether the bridging patterns identified in the current gender comparison are replicated under more stable estimation conditions.

## Conclusion

This study examined the interconnections among parental support, gender stereotypes, passion, and football participation in children using network analysis, guided by ecological systems theory. Harmonious passion emerged as the most central construct, showing strong connections to parental support, participation, and obsessive passion. Gender differences were observed not in global network properties but in local node centrality: obsessive passion and gender stereotypes had higher centrality in boys’ networks, whereas harmonious passion had higher centrality in girls’ networks. The results underscore the need for gender sensitive interventions that target distinct psychosocial mechanisms rather than assuming a one size fits all models. Fostering harmonious passion and addressing the differential roles of stereotypes and obsessive motivation should be prioritized in future efforts to promote equitable and sustained football participation in children.

## Data Availability

The original contributions presented in the study are included in the article/Supplementary material, further inquiries can be directed to the corresponding author/s.
