# Supplementary material for: Psychosocial correlates of children’s football participation: a psychological network analysis of parental support, stereotypes, and dualistic passions
Source: Front Psychol. 2026 Jul 1;17:1897362. doi: 10.3389/fpsyg.2026.1897362 (PMC13368343; doi:10.3389/fpsyg.2026.1897362)
Supplement: Supplementary file 2 [file Table_1.DOCX]

# Main Code

# =============================================

# 07-7_main_analysis.R - 主分析脚本（最终发表版）

# 作者：Marco

# =============================================

cat("=================================================\n")

cat("07-7 性别刻板印象与足球参与：综合网络分析\n")

cat("开始时间：", format(Sys.time(), "%Y-%m-%d %H:%M:%S"), "\n")

cat("=================================================\n\n")

# =============================================

# 0. 初始化 - 强制设置工作目录

# =============================================

setwd("D:/Marco/硕士期间研究/07性别刻板印象对小学生足球参与的影响：代际关系的探究/07-7_SNA/07-7_R代码/")

cat("工作目录:", getwd(), "\n\n")

# =============================================

# 1. 定义分析步骤

# =============================================

analysis_steps <- list(

list(

name = "描述性统计、相关性、信度与效度分析",

file = "01_descriptive_correlation_reliability_validity.R",

output_dir = "output/psychometric_analysis/",

required = TRUE,

exists = file.exists("01_descriptive_correlation_reliability_validity.R")

),

list(

name = "多重共线性检验",

file = "02_multicollinearity.R",

output_dir = "output/multicollinearity/",

required = TRUE,

exists = file.exists("02_multicollinearity.R")

),

list(

name = "综合社交网络分析（两层面）",

file = "03_social_network_analysis.R",

output_dir = "output/",

required = TRUE,

exists = file.exists("03_social_network_analysis.R")

),

list(

name = "性别差异网络分析",

file = "04_group_comparison.R",

output_dir = "output/gender_differences/",

required = FALSE,

exists = file.exists("04_group_comparison.R")

)

)

# =============================================

# 2. 检查文件

# =============================================

cat("检查必需文件...\n")

files_missing <- FALSE

for (step in analysis_steps) {

if (step$required && !step$exists) {

cat("❌ 缺失必需文件:", step$file, "\n")

files_missing <- TRUE

} else if (!step$exists) {

cat("⚠️ 缺失可选文件:", step$file, "（跳过）\n")

} else {

cat("✅ 已找到:", step$file, "\n")

}

}

if (files_missing) {

cat("\n⚠️ 错误：必需文件缺失，请检查工作目录！\n")

cat("当前工作目录:", getwd(), "\n")

stop("文件缺失，终止运行")

}

cat("\n")

# =============================================

# 3. 初始化

# =============================================

results <- list()

start_time_global <- Sys.time()

# =============================================

# 4. 执行分析

# =============================================

for (i in seq_along(analysis_steps)) {

step <- analysis_steps[[i]]

# 跳过不存在的可选文件

if (!step$exists && !step$required) {

cat("\n", paste(rep("=", 70), collapse = ""), "\n")

cat(sprintf("步骤 %d/%d: %s (已跳过 - 文件不存在)\n",

i, length(analysis_steps), step$name))

cat(paste(rep("=", 70), collapse = ""), "\n")

results[[i]] <- list(

step = i,

name = step$name,

status = "跳过",

duration = 0,

error = "文件不存在",

output_dir = step$output_dir

)

next

}

cat("\n", paste(rep("=", 70), collapse = ""), "\n")

cat(sprintf("步骤 %d/%d: %s\n", i, length(analysis_steps), step$name))

cat(paste(rep("=", 70), collapse = ""), "\n")

start_time <- Sys.time()

# 创建输出目录

dir.create(step$output_dir, showWarnings = FALSE, recursive = TRUE)

# 执行脚本

tryCatch({

# 清理环境，但保留必要变量

keep_vars <- c("analysis_steps", "results", "i", "step",

"start_time_global", "start_time")

rm(list = setdiff(ls(), keep_vars), envir = environment())

# =============================================

# 关键修复：注入 start_time 变量，防止01脚本报错

# =============================================

start_time <- Sys.time() # 确保 start_time 存在

# 运行脚本

source(step$file, echo = FALSE, local = TRUE)

end_time <- Sys.time()

duration <- round(as.numeric(difftime(end_time, start_time, units = "mins")), 2)

cat(sprintf("\n✓ 完成 (耗时: %.2f 分钟)\n", duration))

results[[i]] <- list(

step = i,

name = step$name,

status = "成功",

duration = duration,

error = NA,

output_dir = step$output_dir

)

}, error = function(e) {

end_time <- Sys.time()

duration <- round(as.numeric(difftime(end_time, start_time, units = "mins")), 2)

cat(sprintf("\n✗ 失败: %s\n", e$message))

if (step$required) {

cat("⚠️ 该步骤为必需步骤，建议修复后重新运行\n")

} else {

cat("ℹ️ 该步骤为可选步骤，可继续后续分析\n")

}

results[[i]] <<- list(

step = i,

name = step$name,

status = "失败",

duration = duration,

error = e$message,

output_dir = step$output_dir

)

})

# 强制垃圾回收

gc()

}

# =============================================

# 5. 生成汇总报告

# =============================================

cat("\n\n", paste(rep("#", 70), collapse = ""), "\n")

cat(" 分析完成总结\n")

cat(paste(rep("#", 70), collapse = ""), "\n\n")

# 统计

total_duration <- 0

success_count <- 0

required_success <- 0

required_total <- sum(sapply(analysis_steps, function(x) x$required))

optional_total <- sum(!sapply(analysis_steps, function(x) x$required))

optional_success <- 0

# 打印表头

cat(sprintf("%-4s %-10s %-12s %-10s %s\n",

"步骤", "状态", "耗时(分钟)", "必需", "分析内容"))

cat(paste(rep("-", 70), collapse = ""), "\n")

for (i in 1:length(analysis_steps)) {

res <- results[[i]]

step <- analysis_steps[[i]]

if (is.null(res)) {

status <- "未运行"

duration <- 0

} else {

status <- switch(res$status,

"成功" = "✓ 成功",

"失败" = "✗ 失败",

"跳过" = "○ 跳过",

res$status)

duration <- ifelse(is.numeric(res$duration), res$duration, 0)

}

required_flag <- ifelse(step$required, "是", "否")

# 更新计数

if (!is.null(res) && res$status == "成功") {

success_count <- success_count + 1

if (step$required) {

required_success <- required_success + 1

} else {

optional_success <- optional_success + 1

}

}

total_duration <- total_duration + duration

duration_display <- sprintf("%.2f", duration)

# 截断过长的分析名称

name_display <- step$name

if (nchar(name_display) > 35) {

name_display <- paste0(substr(name_display, 1, 32), "...")

}

cat(sprintf("%-4d %-10s %-12s %-10s %s\n",

i, status, duration_display, required_flag, name_display))

# 打印错误信息

if (!is.null(res) && !is.na(res$error) && nchar(res$error) > 0) {

cat(sprintf(" └─ 错误: %s\n", substr(res$error, 1, 60)))

}

}

# 打印汇总行

cat(paste(rep("-", 70), collapse = ""), "\n")

cat(sprintf("总计: 成功 %d/%d (必需: %d/%d, 可选: %d/%d) 总耗时: %.2f 分钟\n",

success_count, length(analysis_steps),

required_success, required_total,

optional_success, optional_total,

total_duration))

cat("\n")

# =============================================

# 6. 检查输出目录

# =============================================

cat(paste(rep(">", 70), collapse = ""), "\n")

cat("输出目录检查\n")

cat(paste(rep(">", 70), collapse = ""), "\n")

output_dirs <- c(

"output/",

"output/psychometric_analysis/",

"output/psychometric_analysis/validity/",

"output/psychometric_analysis/validity/cfa_models/",

"output/multicollinearity/",

"output/figures/",

"output/figures/networks/",

"output/figures/centrality/",

"output/figures/clustering/",

"output/figures/stability/",

"output/tables/",

"output/gender_differences/"

)

for (dir_path in output_dirs) {

if (dir.exists(dir_path)) {

files <- list.files(dir_path, pattern = "\\.(csv|png|pdf|txt|RData)$",

recursive = TRUE, full.names = FALSE)

file_count <- length(files)

if (file_count > 0) {

cat(sprintf("✓ %-40s (%d 个文件)\n", dir_path, file_count))

} else {

cat(sprintf("✓ %-40s (空目录)\n", dir_path))

}

} else {

cat(sprintf(" %-40s (目录不存在)\n", dir_path))

}

}

# =============================================

# 7. 最终建议

# =============================================

cat("\n", paste(rep("+", 70), collapse = ""), "\n")

cat("SCI论文下一步\n")

cat(paste(rep("+", 70), collapse = ""), "\n")

if (required_success == required_total) {

cat("✅ 所有必需分析步骤已成功完成！可以开始撰写论文。\n\n")

cat("推荐写作顺序：\n")

cat(" 1. 方法部分\n")

cat(" 2. 结果部分\n")

cat(" 3. 讨论部分\n")

cat(" 4. 引言与摘要\n")

} else if (required_success >= 2) {

cat("⚠️ 部分必需分析步骤失败，请检查后再撰写论文。\n\n")

} else {

cat("❌ 关键分析步骤失败，需要重新检查。\n\n")

}

# =============================================

# 8. 保存日志

# =============================================

log_file <- file.path("output", paste0("analysis_log_",

format(Sys.time(), "%Y%m%d_%H%M%S"),

".txt"))

log_conn <- file(log_file, "w")

cat("07-7 综合网络分析运行日志\n", file = log_conn)

cat("运行时间:", format(Sys.time(), "%Y-%m-%d %H:%M:%S"), "\n", file = log_conn)

cat("工作目录:", getwd(), "\n\n", file = log_conn)

cat("分析结果汇总:\n", file = log_conn)

for (i in 1:length(analysis_steps)) {

res <- results[[i]]

step <- analysis_steps[[i]]

status <- ifelse(is.null(res), "未运行", res$status)

duration <- ifelse(!is.null(res) && is.numeric(res$duration), res$duration, 0)

error <- ifelse(!is.null(res) && !is.na(res$error), res$error, "无")

cat(sprintf("[%d] %s\n", i, step$name), file = log_conn)

cat(sprintf(" 状态: %s\n", status), file = log_conn)

cat(sprintf(" 耗时: %.2f 分钟\n", duration), file = log_conn)

if (status != "成功" && status != "跳过") {

cat(sprintf(" 错误: %s\n", error), file = log_conn)

}

cat("\n", file = log_conn)

}

close(log_conn)

cat(sprintf("\n📄 运行日志已保存: %s\n", log_file))

# =============================================

# 9. 完成

# =============================================

cat("\n", paste(rep("✓", 70), collapse = ""), "\n")

total_success_rate <- round(success_count / length(analysis_steps) * 100, 1)

required_success_rate <- round(required_success / required_total * 100, 1)

if (required_success == required_total) {

cat(" 🎉 恭喜！所有分析步骤成功完成 🎉\n")

cat(sprintf(" 成功率: 必需 %.1f%%, 总体 %.1f%%\n",

required_success_rate, total_success_rate))

} else if (required_success >= 2) {

cat(sprintf(" 👍 必需步骤成功率: %.1f%% (%d/%d)\n",

required_success_rate, required_success, required_total))

}

cat(paste(rep("✓", 70), collapse = ""), "\n")

cat("\n")

cat("📅 分析完成时间:", format(Sys.time(), "%Y-%m-%d %H:%M:%S"), "\n")

cat("📁 所有结果保存在: output/ 目录\n")

total_time <- difftime(Sys.time(), start_time_global, units = "mins")

cat(sprintf("⏱️ 总运行时间: %.2f 分钟\n", total_time))

if (required_success == required_total) {

cat("🚀 可以开始准备SCI论文投稿了！\n")

} else {

cat("🔧 请修复错误后重新运行主脚本\n")

}

cat("\n")

cat("=================================================\n")

cat(" 脚本结束\n")

cat("=================================================\n")

# 01_descriptive_correlation_reliability_validity

# =============================================

# 01_descriptive_correlation_reliability_validity.R

# Comprehensive Psychometric Analysis for SCI Publication

# =============================================

cat("\n", rep("=", 60), "\n", sep = "")

cat(">>> 心理测量学分析开始: 描述统计、相关、信度、效度 <<<\n")

cat(rep("=", 60), "\n\n", sep = "")

# =============================================

# 1. 加载R包

# =============================================

cat("[1] 加载R包...\n")

required_packages <- c(

"tidyverse", "psych", "ggplot2", "ggcorrplot",

"patchwork", "moments", "corrplot", "lavaan",

"semTools", "dplyr", "tidyr", "reshape2",

"RColorBrewer", "performance", "knitr", "kableExtra"

)

# 安装缺失包

new_packages <- required_packages[!(required_packages %in% installed.packages()[,"Package"])]

if(length(new_packages) > 0) {

cat(" 安装缺失包:", paste(new_packages, collapse = ", "), "\n")

install.packages(new_packages, dependencies = TRUE)

}

# 加载包

suppressPackageStartupMessages({

library(tidyverse)

library(psych)

library(ggplot2)

library(ggcorrplot)

library(patchwork)

library(moments)

library(corrplot)

library(lavaan)

library(semTools)

library(RColorBrewer)

library(performance)

library(knitr)

library(kableExtra)

})

cat(" ✓ 包加载完成\n\n")

# =============================================

# 2. 设置路径

# =============================================

cat("[2] 设置输出路径...\n")

RAW_DATA_PATH <- "D:/Marco/analysis/R/07/07_416.csv"

OUTPUT_DIR <- "D:/Marco/硕士期间研究/07性别刻板印象对小学生足球参与的影响：代际关系的探究/07-7_SNA/07-7_R代码/output"

# 创建分析目录

ANALYSIS_DIR <- file.path(OUTPUT_DIR, "psychometric_analysis")

DESCRIPTIVE_DIR <- file.path(ANALYSIS_DIR, "descriptive")

CORRELATION_DIR <- file.path(ANALYSIS_DIR, "correlation")

RELIABILITY_DIR <- file.path(ANALYSIS_DIR, "reliability")

VALIDITY_DIR <- file.path(ANALYSIS_DIR, "validity")

CFA_DIR <- file.path(VALIDITY_DIR, "cfa_models") # 新增：CFA模型比较目录

dir.create(ANALYSIS_DIR, showWarnings = FALSE, recursive = TRUE)

dir.create(DESCRIPTIVE_DIR, showWarnings = FALSE, recursive = TRUE)

dir.create(CORRELATION_DIR, showWarnings = FALSE, recursive = TRUE)

dir.create(RELIABILITY_DIR, showWarnings = FALSE, recursive = TRUE)

dir.create(VALIDITY_DIR, showWarnings = FALSE, recursive = TRUE)

dir.create(CFA_DIR, showWarnings = FALSE, recursive = TRUE)

cat(" ✓ 输出目录创建完成\n")

cat(" 📁 主目录:", ANALYSIS_DIR, "\n\n")

# =============================================

# 3. 定义变量和构念映射

# =============================================

cat("[3] 定义变量和构念映射...\n")

# 变量名

variable_names <- c(

"Age", "Gender", # 人口学

"GS1", "GS2", "GS3", "GS4", # 性别刻板印象

"HP1", "HP3", "HP5", "HP6", "HP8", "HP10", # 和谐热情

"OP2", "OP4", "OP7", "OP9", "OP11", "OP12", # 执着热情

"PS1", "PS2", "PS3", "PS4", # 父母支持

"FP1", "FP2" # 足球参与

)

# 构念映射

construct_mapping <- list(

GS = list(

name = "Gender Stereotypes",

items = c("GS1", "GS2", "GS3", "GS4"),

full_name = "Gender Stereotypes"

),

HP = list(

name = "Harmonious Passion",

items = c("HP1", "HP3", "HP5", "HP6", "HP8", "HP10"),

full_name = "Harmonious Passion"

),

OP = list(

name = "Obsessive Passion",

items = c("OP2", "OP4", "OP7", "OP9", "OP11", "OP12"),

full_name = "Obsessive Passion"

),

PS = list(

name = "Parental Support",

items = c("PS1", "PS2", "PS3", "PS4"),

full_name = "Parental Support"

),

FP = list(

name = "Football Participation",

items = c("FP1", "FP2"),

full_name = "Football Participation"

)

)

cat(" ✓ 构念映射定义完成\n")

cat(" 5个构念, 22个条目\n\n")

# =============================================

# 4. 数据读取与清洗

# =============================================

cat("[4] 读取并清洗数据...\n")

# 4.1 读取数据

raw_data <- read.csv(RAW_DATA_PATH, header = FALSE, stringsAsFactors = FALSE)

cat(" 原始数据:", nrow(raw_data), "行 ×", ncol(raw_data), "列\n")

# 4.2 列名检查与赋值

if(length(variable_names) != ncol(raw_data)) {

cat(" ⚠️ 列数不匹配, 期望", length(variable_names), "列, 实际", ncol(raw_data), "列\n")

variable_names <- variable_names[1:ncol(raw_data)]

}

colnames(raw_data) <- variable_names

cat(" ✓ 变量名赋值完成\n")

# 4.3 处理缺失值 (999 -> NA)

for(col in variable_names) {

if(is.numeric(raw_data[[col]])) {

raw_data[[col]][raw_data[[col]] == 999] <- NA

}

}

# 4.4 性别转换为因子

raw_data$Gender <- factor(raw_data$Gender,

levels = c(1, 2),

labels = c("Male", "Female"))

# 4.5 提取条目数据

item_data <- raw_data[, !names(raw_data) %in% c("Age", "Gender")]

# 4.6 删除缺失过多的个案

keep <- rowMeans(is.na(item_data)) <= 0.3

item_data_clean <- item_data[keep, ]

raw_data_clean <- raw_data[keep, ]

cat(" 有效样本:", sum(keep), "/", nrow(raw_data),

"(", round(mean(keep)*100, 1), "%)\n")

# 4.7 中位数填补

impute_median <- function(x) {

x[is.na(x)] <- median(x, na.rm = TRUE)

return(x)

}

item_data_complete <- as.data.frame(lapply(item_data_clean, impute_median))

raw_data_complete <- raw_data_clean

raw_data_complete[, names(item_data_complete)] <- item_data_complete

n <- nrow(item_data_complete)

cat(" 最终样本量 N =", n, "\n\n")

# =============================================

# 5. 计算构念得分

# =============================================

cat("[5] 计算构念得分...\n")

construct_scores <- data.frame(

Age = raw_data_complete$Age,

Gender = raw_data_complete$Gender

)

for(construct in names(construct_mapping)) {

items <- construct_mapping[[construct]]$items

available_items <- intersect(items, colnames(item_data_complete))

if(length(available_items) > 0) {

construct_scores[[construct]] <- rowMeans(

item_data_complete[, available_items, drop = FALSE],

na.rm = TRUE

)

cat(" ", construct_mapping[[construct]]$full_name,

": ", length(available_items), " items\n", sep = "")

}

}

# 保存构念得分

write.csv(construct_scores,

file.path(ANALYSIS_DIR, "01_construct_scores.csv"),

row.names = FALSE)

cat(" ✓ 构念得分已保存\n\n")

# =============================================

# 6. 描述性统计

# =============================================

cat("[6] 计算描述性统计...\n")

# 6.1 人口学统计

demographic_stats <- data.frame(

Variable = c("Age", "Gender (Male/Female)"),

N = c(

sum(!is.na(construct_scores$Age)),

sum(!is.na(construct_scores$Gender))

),

Mean_SD = c(

paste0(round(mean(construct_scores$Age, na.rm = TRUE), 2),

" (", round(sd(construct_scores$Age, na.rm = TRUE), 2), ")"),

paste0(table(construct_scores$Gender)[1], "/", table(construct_scores$Gender)[2])

),

Percent = c(

"",

paste0(round(prop.table(table(construct_scores$Gender))[1] * 100, 1), "%/",

round(prop.table(table(construct_scores$Gender))[2] * 100, 1), "%") # ← 修复这里！

),

Range = c(

paste0(round(min(construct_scores$Age, na.rm = TRUE), 0), "-",

round(max(construct_scores$Age, na.rm = TRUE), 0)),

""

),

stringsAsFactors = FALSE

)

# 6.2 构念描述统计

construct_desc <- data.frame()

for(construct in names(construct_mapping)) {

scores <- construct_scores[[construct]]

if(!all(is.na(scores))) {

scores_clean <- na.omit(scores)

# 正态性检验

shapiro_test <- tryCatch({

shapiro.test(scores_clean)

}, error = function(e) {

list(statistic = NA, p.value = NA)

})

construct_desc <- rbind(construct_desc, data.frame(

Construct = construct,

Full_Name = construct_mapping[[construct]]$full_name,

N = length(scores_clean),

Mean = round(mean(scores_clean), 2),

SD = round(sd(scores_clean), 2),

Min = round(min(scores_clean), 2),

Max = round(max(scores_clean), 2),

Skewness = round(skewness(scores_clean), 2),

Kurtosis = round(kurtosis(scores_clean) - 3, 2), # 超值峰度

Shapiro_W = round(shapiro_test$statistic, 3),

Shapiro_p = shapiro_test$p.value,

stringsAsFactors = FALSE

))

}

}

# 保存描述统计

write.csv(demographic_stats,

file.path(DESCRIPTIVE_DIR, "01_demographic_statistics.csv"),

row.names = FALSE)

write.csv(construct_desc,

file.path(DESCRIPTIVE_DIR, "02_construct_descriptives.csv"),

row.names = FALSE)

cat(" ✓ 描述性统计已保存\n")

print(kable(construct_desc[, 1:9],

caption = "Descriptive Statistics",

format = "simple"))

cat("\n")

# =============================================

# 7. 相关分析

# =============================================

cat("[7] 相关分析...\n")

# 7.1 准备相关数据

cor_data <- construct_scores %>%

mutate(Gender_num = as.numeric(Gender) - 1) # Male=0, Female=1

cor_vars <- c("Age", "Gender_num", names(construct_mapping))

cor_matrix_data <- cor_data[, cor_vars]

# 7.2 计算相关系数和p值

cor_matrix <- cor(cor_matrix_data, use = "pairwise.complete.obs")

p_matrix <- corr.test(cor_matrix_data, adjust = "none")$p

# 7.3 保存相关矩阵

write.csv(round(cor_matrix, 3),

file.path(CORRELATION_DIR, "01_correlation_matrix.csv"),

row.names = TRUE)

write.csv(round(p_matrix, 4),

file.path(CORRELATION_DIR, "02_correlation_pvalues.csv"),

row.names = TRUE)

cat(" ✓ 相关矩阵已保存\n")

# 7.4 创建相关热图

var_labels <- c("Age", "Gender", "GS", "HP", "OP", "PS", "FP")

full_labels <- c("Age", "Gender", "Gender Stereotypes",

"Harmonious Passion", "Obsessive Passion",

"Parental Support", "Football Participation")

plot_data <- reshape2::melt(cor_matrix)

plot_data$Var1 <- factor(plot_data$Var1, levels = rev(var_labels))

plot_data$Var2 <- factor(plot_data$Var2, levels = var_labels)

# 只显示上三角

plot_data <- plot_data %>%

filter(as.numeric(Var1) < as.numeric(Var2))

# 添加显著性标记

p_melted <- reshape2::melt(p_matrix)

plot_data <- merge(plot_data, p_melted, by = c("Var1", "Var2"))

names(plot_data) <- c("Var1", "Var2", "Correlation", "P_Value")

plot_data$Stars <- ""

plot_data$Stars[plot_data$P_Value < 0.05] <- "*"

plot_data$Stars[plot_data$P_Value < 0.01] <- "**"

plot_data$Stars[plot_data$P_Value < 0.001] <- "***"

plot_data$Label <- sprintf("%.2f%s", round(plot_data$Correlation, 2), plot_data$Stars)

# 绘制热图

p_correlation <- ggplot(plot_data, aes(x = Var2, y = Var1, fill = Correlation)) +

geom_tile(color = "white", linewidth = 0.5) +

geom_text(aes(label = Label), size = 4, fontface = "bold") +

scale_fill_gradient2(low = "#2E86AB", mid = "white", high = "#F24236",

midpoint = 0, limits = c(-1, 1), name = "r") +

scale_x_discrete(labels = full_labels, position = "top") +

scale_y_discrete(labels = rev(full_labels)) +

labs(title = "Correlation Matrix",

subtitle = paste("N =", nrow(cor_matrix_data)),

x = "", y = "",

caption = "* p < .05, ** p < .01, *** p < .001") +

theme_minimal(base_size = 11) +

theme(

plot.title = element_text(face = "bold", hjust = 0.5, size = 14),

plot.subtitle = element_text(hjust = 0.5, size = 11),

axis.text.x = element_text(angle = 0, hjust = 0.5, size = 10,

margin = margin(t = 5)),

axis.text.y = element_text(size = 10),

panel.grid = element_blank(),

legend.position = "right"

) +

coord_fixed(ratio = 0.8)

ggsave(file.path(CORRELATION_DIR, "03_correlation_heatmap.png"),

p_correlation, width = 10, height = 8, dpi = 300)

cat(" ✓ 相关热图已保存\n\n")

# =============================================

# 8. 信度分析

# =============================================

cat("[8] 信度分析...\n")

reliability_results <- data.frame()

for(construct in names(construct_mapping)) {

items <- construct_mapping[[construct]]$items

item_data_temp <- item_data_complete[, intersect(items, colnames(item_data_complete))]

if(ncol(item_data_temp) >= 2) {

# Cronbach's alpha

alpha_result <- psych::alpha(item_data_temp, check.keys = TRUE)

# McDonald's omega

omega_result <- tryCatch({

psych::omega(item_data_temp, nfactors = 1, plot = FALSE)

}, error = function(e) {

list(omega.tot = NA, omega.lim = NA)

})

reliability_results <- rbind(reliability_results, data.frame(

Construct = construct,

Full_Name = construct_mapping[[construct]]$full_name,

N_Items = ncol(item_data_temp),

N_Sample = nrow(item_data_temp),

Cronbach_Alpha = round(alpha_result$total$raw_alpha, 3),

Omega_Total = round(omega_result$omega.tot, 3),

stringsAsFactors = FALSE

))

cat(" ", construct_mapping[[construct]]$full_name,

": α = ", round(alpha_result$total$raw_alpha, 3),

", ω = ", round(omega_result$omega.tot, 3), "\n", sep = "")

}

}

# 保存信度结果

write.csv(reliability_results,

file.path(RELIABILITY_DIR, "01_reliability_results.csv"),

row.names = FALSE)

cat(" ✓ 信度结果已保存\n\n")

# =============================================

# 9. 验证性因子分析 - 完整版（单维度+整体模型比较）

# =============================================

cat("\n[9] 验证性因子分析...\n")

# 9.1 准备CFA数据

cfa_data <- item_data_complete[, unlist(lapply(construct_mapping, function(x) x$items))]

n_cfa <- nrow(cfa_data)

cat(" CFA样本量: N =", n_cfa, "\n\n")

# =============================================

# 9.2 单维度CFA：每个构念单独验证 - 修复版

# =============================================

cat(" [9.2] 单维度CFA分析 (MLR估计)...\n")

single_cfa_results <- list()

for(construct in names(construct_mapping)) {

cat(" 正在拟合:", construct_mapping[[construct]]$full_name, "... ")

items <- construct_mapping[[construct]]$items

available_items <- intersect(items, colnames(cfa_data))

n_items <- length(available_items)

if(n_items >= 3) {

# 3个及以上条目：标准模型，固定因子方差为1

model_single <- paste0(construct, " =~ ",

paste(available_items, collapse = " + "))

tryCatch({

fit_single <- cfa(model_single,

data = cfa_data,

estimator = "MLR",

missing = "fiml",

std.lv = TRUE) # 固定因子方差为1

# 提取拟合指数

fit_indices <- fitMeasures(fit_single,

c("chisq", "df", "pvalue",

"cfi", "tli", "rmsea", "srmr"))

# 提取因子载荷

loadings <- parameterEstimates(fit_single, standardized = TRUE) %>%

filter(op == "=~") %>%

select(Item = rhs, Std_Loading = std.all, pvalue)

single_cfa_results[[construct]] <- list(

fit = fit_single,

indices = fit_indices,

loadings = loadings

)

cat("✓ CFI =", round(fit_indices["cfi"], 3),

", RMSEA =", round(fit_indices["rmsea"], 3),

", SRMR =", round(fit_indices["srmr"], 3), "\n")

}, error = function(e) {

cat("✗ 失败:", e$message, "\n")

single_cfa_results[[construct]] <- NULL

})

} else if(n_items == 2) {

# 2个条目：饱和模型，固定载荷相等

cat("[2条目饱和模型] ")

tryCatch({

# 方法：固定两个载荷相等，自由估计因子方差

model_two <- paste0(

construct, " =~ a*", available_items[1], " + a*", available_items[2], "\n",

construct, " ~~ ", construct

)

fit_single <- cfa(model_two,

data = cfa_data,

estimator = "MLR",

missing = "fiml")

# 饱和模型，只提取载荷

loadings <- parameterEstimates(fit_single, standardized = TRUE) %>%

filter(op == "=~") %>%

select(Item = rhs, Std_Loading = std.all, pvalue)

single_cfa_results[[construct]] <- list(

fit = fit_single,

indices = NULL, # 饱和模型无拟合指数

loadings = loadings

)

cat("✓ 载荷 =", round(mean(loadings$Std_Loading), 3), "\n")

}, error = function(e) {

cat("✗ 失败:", e$message, "\n")

single_cfa_results[[construct]] <- NULL

})

}

}

# 9.2.1 汇总单维度CFA结果

single_cfa_summary <- data.frame()

for(construct in names(single_cfa_results)) {

if(!is.null(single_cfa_results[[construct]])) {

if(construct == "FP") {

# FP是2条目饱和模型，无拟合指数

loadings <- single_cfa_results[[construct]]$loadings

single_cfa_summary <- rbind(single_cfa_summary, data.frame(

Construct = construct,

Full_Name = construct_mapping[[construct]]$full_name,

N_Items = length(construct_mapping[[construct]]$items),

Chi2 = NA,

df = 0,

p_value = NA,

CFI = 1.00, # 饱和模型CFI=1

TLI = 1.00, # 饱和模型TLI=1

RMSEA = 0.00, # 饱和模型RMSEA=0

SRMR = 0.00, # 饱和模型SRMR=0

Min_Loading = round(min(loadings$Std_Loading), 3),

Max_Loading = round(max(loadings$Std_Loading), 3),

Mean_Loading = round(mean(loadings$Std_Loading), 3),

stringsAsFactors = FALSE

))

} else {

# 3+条目模型

indices <- single_cfa_results[[construct]]$indices

loadings <- single_cfa_results[[construct]]$loadings

single_cfa_summary <- rbind(single_cfa_summary, data.frame(

Construct = construct,

Full_Name = construct_mapping[[construct]]$full_name,

N_Items = length(construct_mapping[[construct]]$items),

Chi2 = round(indices["chisq"], 2),

df = indices["df"],

p_value = round(indices["pvalue"], 3),

CFI = round(indices["cfi"], 3),

TLI = round(indices["tli"], 3),

RMSEA = round(indices["rmsea"], 3),

SRMR = round(indices["srmr"], 3),

Min_Loading = round(min(loadings$Std_Loading), 3),

Max_Loading = round(max(loadings$Std_Loading), 3),

Mean_Loading = round(mean(loadings$Std_Loading), 3),

stringsAsFactors = FALSE

))

}

}

}

# 保存单维度CFA结果

write.csv(single_cfa_summary,

file.path(CFA_DIR, "02_single_factor_cfa_summary.csv"),

row.names = FALSE)

cat("\n ✓ 单维度CFA结果已保存\n")

print(kable(single_cfa_summary,

caption = "Single-Factor CFA Results by Construct",

format = "simple", digits = 3))

cat("\n")

# =============================================

# 9.3 单维度因子载荷图

# =============================================

cat(" [9.3] 生成单维度因子载荷图...\n")

# 合并所有单维度的载荷

all_loadings_single <- data.frame()

for(construct in names(single_cfa_results)) {

if(!is.null(single_cfa_results[[construct]])) {

temp_loadings <- single_cfa_results[[construct]]$loadings

temp_loadings$Construct <- construct

temp_loadings$Full_Name <- construct_mapping[[construct]]$full_name

all_loadings_single <- rbind(all_loadings_single, temp_loadings)

}

}

if(nrow(all_loadings_single) > 0) {

p_single_loadings <- ggplot(all_loadings_single,

aes(x = reorder(Item, Std_Loading),

y = Std_Loading,

fill = Full_Name)) +

geom_bar(stat = "identity", width = 0.7) +

geom_hline(yintercept = 0.40, linetype = "dashed", color = "red", alpha = 0.7) +

geom_hline(yintercept = 0.70, linetype = "dashed", color = "blue", alpha = 0.7) +

geom_text(aes(label = round(Std_Loading, 2)),

vjust = -0.5, size = 3, fontface = "bold") +

facet_wrap(~ Full_Name, scales = "free_y", ncol = 2) +

coord_flip() +

scale_fill_brewer(palette = "Set1") +

labs(title = "Single-Factor CFA: Standardized Loadings",

subtitle = paste("N =", n_cfa, "| MLR Estimator"),

x = "Item", y = "Standardized Factor Loading",

caption = "Red: .40 (min) | Blue: .70 (excellent)") +

theme_minimal(base_size = 11) +

theme(

plot.title = element_text(face = "bold", hjust = 0.5, size = 14),

plot.subtitle = element_text(hjust = 0.5, size = 11),

legend.position = "none",

strip.text = element_text(face = "bold", size = 10),

panel.spacing = unit(1, "lines")

) +

ylim(0, 1)

ggsave(file.path(CFA_DIR, "03_single_factor_loadings.png"),

p_single_loadings, width = 14, height = 10, dpi = 300)

ggsave(file.path(CFA_DIR, "03_single_factor_loadings.pdf"),

p_single_loadings, width = 14, height = 10, device = "pdf")

cat(" ✓ 单维度因子载荷图已保存\n")

}

# =============================================

# 9.4 整体模型比较（竞争模型）- MLR估计

# =============================================

cat("\n [9.4] 整体模型比较分析 (MLR估计)...\n")

# 定义所有竞争模型

cfa_models <- list()

# 模型1: 单因子模型

cfa_models[["M1_OneFactor"]] <- '

General =~ GS1 + GS2 + GS3 + GS4 +

HP1 + HP3 + HP5 + HP6 + HP8 + HP10 +

OP2 + OP4 + OP7 + OP9 + OP11 + OP12 +

PS1 + PS2 + PS3 + PS4 +

FP1 + FP2

'

# 模型2: 二因子模型 (性别刻板印象 vs 其他)

cfa_models[["M2_TwoFactor"]] <- '

GS =~ GS1 + GS2 + GS3 + GS4

Others =~ HP1 + HP3 + HP5 + HP6 + HP8 + HP10 +

OP2 + OP4 + OP7 + OP9 + OP11 + OP12 +

PS1 + PS2 + PS3 + PS4 +

FP1 + FP2

GS ~~ Others

'

# 模型3: 三因子模型 (GS, Passion, Outcome)

cfa_models[["M3_ThreeFactor"]] <- '

GS =~ GS1 + GS2 + GS3 + GS4

Passion =~ HP1 + HP3 + HP5 + HP6 + HP8 + HP10 +

OP2 + OP4 + OP7 + OP9 + OP11 + OP12

Outcome =~ PS1 + PS2 + PS3 + PS4 + FP1 + FP2

GS ~~ Passion + Outcome

Passion ~~ Outcome

'

# 模型4: 四因子模型 (GS, HP, OP, PS+FP)

cfa_models[["M4_FourFactor"]] <- '

GS =~ GS1 + GS2 + GS3 + GS4

HP =~ HP1 + HP3 + HP5 + HP6 + HP8 + HP10

OP =~ OP2 + OP4 + OP7 + OP9 + OP11 + OP12

Support =~ PS1 + PS2 + PS3 + PS4 + FP1 + FP2

GS ~~ HP + OP + Support

HP ~~ OP + Support

OP ~~ Support

'

# 模型5: 五因子模型 (理论模型)

cfa_models[["M5_FiveFactor"]] <- '

GS =~ GS1 + GS2 + GS3 + GS4

HP =~ HP1 + HP3 + HP5 + HP6 + HP8 + HP10

OP =~ OP2 + OP4 + OP7 + OP9 + OP11 + OP12

PS =~ PS1 + PS2 + PS3 + PS4

FP =~ FP1 + FP2

'

# 模型6: 六因子模型 (含方法因子，检验CMV)

cfa_models[["M6_SixFactor_CMV"]] <- '

GS =~ GS1 + GS2 + GS3 + GS4

HP =~ HP1 + HP3 + HP5 + HP6 + HP8 + HP10

OP =~ OP2 + OP4 + OP7 + OP9 + OP11 + OP12

PS =~ PS1 + PS2 + PS3 + PS4

FP =~ FP1 + FP2

CMV =~ GS1 + GS2 + GS3 + GS4 +

HP1 + HP3 + HP5 + HP6 + HP8 + HP10 +

OP2 + OP4 + OP7 + OP9 + OP11 + OP12 +

PS1 + PS2 + PS3 + PS4 +

FP1 + FP2

GS ~~ 0*CMV

HP ~~ 0*CMV

OP ~~ 0*CMV

PS ~~ 0*CMV

FP ~~ 0*CMV

'

# 拟合所有模型

model_fits <- list()

model_summary <- data.frame()

for(model_name in names(cfa_models)) {

cat(" 拟合:", model_name, "... ")

tryCatch({

fit <- cfa(cfa_models[[model_name]],

data = cfa_data,

estimator = "MLR", # 稳健标准误，处理非正态数据

missing = "fiml", # 全信息最大似然处理缺失值

std.lv = TRUE) # 标准化潜变量

# 提取拟合指数（MLR会自动使用Satorra-Bentler校正卡方）

fit_indices <- fitMeasures(fit,

c("chisq", "df", "pvalue",

"cfi", "tli", "rmsea", "rmsea.ci.lower",

"rmsea.ci.upper", "srmr", "aic", "bic"))

model_fits[[model_name]] <- fit

# 添加到汇总表

model_summary <- rbind(model_summary, data.frame(

Model = model_name,

Description = switch(model_name,

M1_OneFactor = "单因子模型",

M2_TwoFactor = "二因子模型",

M3_ThreeFactor = "三因子模型",

M4_FourFactor = "四因子模型",

M5_FiveFactor = "五因子模型 (理论模型)",

M6_SixFactor_CMV = "六因子模型 (含方法因子)"),

Chi2 = round(fit_indices["chisq"], 2),

df = fit_indices["df"],

p_value = round(fit_indices["pvalue"], 3),

CFI = round(fit_indices["cfi"], 3),

TLI = round(fit_indices["tli"], 3),

RMSEA = round(fit_indices["rmsea"], 3),

RMSEA_CI = paste0("[", round(fit_indices["rmsea.ci.lower"], 3),

", ", round(fit_indices["rmsea.ci.upper"], 3), "]"),

SRMR = round(fit_indices["srmr"], 3),

AIC = round(fit_indices["aic"], 1),

BIC = round(fit_indices["bic"], 1),

stringsAsFactors = FALSE

))

cat("✓ CFI =", round(fit_indices["cfi"], 3),

", RMSEA =", round(fit_indices["rmsea"], 3),

", SRMR =", round(fit_indices["srmr"], 3),

", 估计法: MLR\n")

}, error = function(e) {

cat("✗ 失败:", e$message, "\n")

})

}

# 9.4.1 添加模型比较列（以五因子模型为基准）

if(nrow(model_summary) > 1) {

if("M5_FiveFactor" %in% model_summary$Model) {

base_fit <- model_summary[model_summary$Model == "M5_FiveFactor", ]

model_summary$Delta_Chi2 <- NA

model_summary$Delta_df <- NA

model_summary$Delta_p <- NA

model_summary$Delta_CFI <- NA

for(i in 1:nrow(model_summary)) {

if(model_summary$Model[i] != "M5_FiveFactor") {

model_summary$Delta_Chi2[i] <- round(model_summary$Chi2[i] - base_fit$Chi2, 2)

model_summary$Delta_df[i] <- model_summary$df[i] - base_fit$df

model_summary$Delta_CFI[i] <- round(model_summary$CFI[i] - base_fit$CFI, 3)

if(!is.na(model_summary$Delta_Chi2[i]) &&

!is.na(model_summary$Delta_df[i]) &&

model_summary$Delta_df[i] > 0) {

model_summary$Delta_p[i] <- round(pchisq(model_summary$Delta_Chi2[i],

model_summary$Delta_df[i],

lower.tail = FALSE), 3)

}

}

}

}

}

# 保存模型比较结果

write.csv(model_summary,

file.path(CFA_DIR, "04_cfa_model_comparison.csv"),

row.names = FALSE)

cat("\n ✓ 模型比较结果已保存至:", file.path(CFA_DIR, "04_cfa_model_comparison.csv"), "\n")

# 显示模型比较表格

cat("\n")

cat(paste(rep("=", 100), collapse = ""), "\n")

cat("整体模型比较结果 (MLR估计)\n")

cat(paste(rep("=", 100), collapse = ""), "\n\n")

model_display <- model_summary %>%

select(Model, Description, Chi2, df, p_value, CFI, TLI, RMSEA, SRMR, AIC, BIC,

Delta_Chi2, Delta_df, Delta_p, Delta_CFI)

print(kable(model_display,

caption = "CFA Model Comparison (MLR Estimator)",

format = "simple",

digits = 3,

format.args = list(nsmall = 3)))

cat("\n")

cat(" 模型选择标准:\n")

cat(" • CFI ≥ .90, TLI ≥ .90, RMSEA ≤ .08, SRMR ≤ .08 表示拟合良好\n")

cat(" • ΔCFI ≤ .01 表示模型无显著差异 (Chen, 2007)\n")

cat(" • AIC/BIC越小越好\n")

cat(" • 五因子模型为理论模型\n\n")

# 识别最佳模型

if(nrow(model_summary) > 0) {

# 根据CFI, RMSEA, SRMR, AIC综合判断

model_summary$CFI_score <- -model_summary$CFI # 负值，因为越大越好

model_summary$RMSEA_score <- model_summary$RMSEA

model_summary$SRMR_score <- model_summary$SRMR

model_summary$AIC_score <- scale(model_summary$AIC)[,1]

# 综合得分（越低越好）

score_matrix <- cbind(

scale(model_summary$CFI_score)[,1],

scale(model_summary$RMSEA_score)[,1],

scale(model_summary$SRMR_score)[,1],

scale(model_summary$AIC)[,1]

)

model_summary$Composite_Score <- rowMeans(score_matrix, na.rm = TRUE)

model_summary <- model_summary %>%

arrange(Composite_Score) %>%

mutate(Rank = 1:nrow(.))

best_model <- model_summary$Model[1]

cat(" 🏆 最佳模型:", best_model, "-", model_summary$Description[1], "\n")

cat(" 综合评分排名第1，推荐作为最终模型\n\n")

}

# 输出模型拟合度解读

cat(" 拟合度解读:\n")

for(i in 1:nrow(model_summary)) {

cat(" • ", model_summary$Description[i], ":\n", sep = "")

cat(" CFI = ", model_summary$CFI[i],

ifelse(model_summary$CFI[i] >= 0.90, " ✓", " ⚠️"),

", RMSEA = ", model_summary$RMSEA[i],

ifelse(model_summary$RMSEA[i] <= 0.08, " ✓", " ⚠️"),

", SRMR = ", model_summary$SRMR[i],

ifelse(model_summary$SRMR[i] <= 0.08, " ✓", " ⚠️"), "\n", sep = "")

}

cat("\n")

# =============================================

# 9.5 最佳模型详细结果（五因子模型）

# =============================================

cat("\n [9.5] 五因子模型详细结果...\n")

if("M5_FiveFactor" %in% names(model_fits)) {

final_fit <- model_fits[["M5_FiveFactor"]]

# 9.5.1 因子载荷

loadings <- parameterEstimates(final_fit, standardized = TRUE) %>%

filter(op == "=~") %>%

select(Factor = lhs, Item = rhs, Loading = est,

Std_Loading = std.all, SE = se, Z = z, pvalue)

write.csv(loadings,

file.path(VALIDITY_DIR, "01_factor_loadings.csv"),

row.names = FALSE)

# 9.5.2 因子相关

correlations <- parameterEstimates(final_fit, standardized = TRUE) %>%

filter(op == "~~", lhs != rhs,

lhs %in% names(construct_mapping),

rhs %in% names(construct_mapping)) %>%

select(Factor1 = lhs, Factor2 = rhs, Correlation = est,

Std_Correlation = std.all, SE = se, Z = z, pvalue)

write.csv(correlations,

file.path(VALIDITY_DIR, "02_factor_correlations.csv"),

row.names = FALSE)

# 9.5.3 计算CR和AVE

reliability_validity <- data.frame()

for(construct in names(construct_mapping)) {

construct_loadings <- loadings %>%

filter(Factor == construct) %>%

pull(Std_Loading)

if(length(construct_loadings) > 0) {

sum_loadings <- sum(construct_loadings)

sum_sq_loadings <- sum(construct_loadings^2)

n_items <- length(construct_loadings)

# Composite Reliability

cr <- (sum_loadings^2) / (sum_loadings^2 + (n_items - sum_sq_loadings))

# Average Variance Extracted

ave <- sum_sq_loadings / n_items

# Maximum Shared Variance

construct_cors <- correlations %>%

filter(Factor1 == construct | Factor2 == construct) %>%

pull(Std_Correlation)

msv <- ifelse(length(construct_cors) > 0, max(construct_cors^2, na.rm = TRUE), NA)

asv <- ifelse(length(construct_cors) > 0, mean(construct_cors^2, na.rm = TRUE), NA)

# Fornell-Larcker criterion

fornell_larcker <- ifelse(!is.na(ave) && !is.na(max(abs(construct_cors))),

sqrt(ave) > max(abs(construct_cors), na.rm = TRUE),

NA)

reliability_validity <- rbind(reliability_validity, data.frame(

Construct = construct,

Full_Name = construct_mapping[[construct]]$full_name,

N_Items = n_items,

CR = round(cr, 3),

AVE = round(ave, 3),

MSV = round(msv, 3),

ASV = round(asv, 3),

sqrt_AVE = round(sqrt(ave), 3),

Max_Correlation = round(max(abs(construct_cors), na.rm = TRUE), 3),

Fornell_Larcker = fornell_larcker,

stringsAsFactors = FALSE

))

}

}

write.csv(reliability_validity,

file.path(VALIDITY_DIR, "03_reliability_validity.csv"),

row.names = FALSE)

cat("\n ✓ 五因子模型详细结果已保存\n")

# 显示CR/AVE结果

cat("\n")

cat(paste(rep("-", 80), collapse = ""), "\n")

cat("复合信度(CR)与收敛/区分效度\n")

cat(paste(rep("-", 80), collapse = ""), "\n")

rv_display <- reliability_validity %>%

select(Construct, Full_Name, CR, AVE, MSV, Fornell_Larcker)

print(kable(rv_display,

caption = "Composite Reliability and Validity",

format = "simple",

digits = 3))

cat("\n")

# 9.5.4 五因子模型因子载荷图

p_loadings <- loadings %>%

mutate(Factor_Label = case_when(

Factor == "GS" ~ "Gender Stereotypes",

Factor == "HP" ~ "Harmonious Passion",

Factor == "OP" ~ "Obsessive Passion",

Factor == "PS" ~ "Parental Support",

Factor == "FP" ~ "Football Participation",

TRUE ~ Factor

)) %>%

ggplot(aes(x = reorder(Item, Std_Loading), y = Std_Loading, fill = Factor_Label)) +

geom_bar(stat = "identity", width = 0.7) +

geom_hline(yintercept = 0.40, linetype = "dashed", color = "red", alpha = 0.7) +

geom_hline(yintercept = 0.70, linetype = "dashed", color = "blue", alpha = 0.7) +

geom_text(aes(label = round(Std_Loading, 2)),

vjust = -0.5, size = 3, fontface = "bold") +

facet_wrap(~ Factor_Label, scales = "free_y", ncol = 2) +

coord_flip() +

scale_fill_brewer(palette = "Set1") +

labs(title = "Five-Factor CFA: Standardized Loadings",

subtitle = paste("N =", n_cfa, "| MLR Estimator"),

x = "Item", y = "Standardized Factor Loading",

caption = "Red: .40 (min) | Blue: .70 (excellent)") +

theme_minimal(base_size = 11) +

theme(

plot.title = element_text(face = "bold", hjust = 0.5, size = 14),

plot.subtitle = element_text(hjust = 0.5, size = 11),

legend.position = "none",

strip.text = element_text(face = "bold", size = 10),

panel.spacing = unit(1, "lines")

) +

ylim(0, 1)

ggsave(file.path(VALIDITY_DIR, "04_factor_loadings_plot.png"),

p_loadings, width = 14, height = 10, dpi = 300)

ggsave(file.path(VALIDITY_DIR, "04_factor_loadings_plot.pdf"),

p_loadings, width = 14, height = 10, device = "pdf")

cat(" ✓ 五因子模型因子载荷图已保存\n")

# 9.5.5 因子相关热图

cat(" 生成因子相关热图...\n")

# 创建因子相关矩阵

cor_matrix_factors <- correlations %>%

select(Factor1, Factor2, Std_Correlation) %>%

pivot_wider(names_from = Factor2, values_from = Std_Correlation)

cor_mat <- as.matrix(cor_matrix_factors[, -1])

rownames(cor_mat) <- cor_matrix_factors$Factor1

# 定义因子标签

factor_labels <- c(

"GS" = "Gender Stereotypes",

"HP" = "Harmonious Passion",

"OP" = "Obsessive Passion",

"PS" = "Parental Support",

"FP" = "Football Participation"

)

# 转换为长格式

cor_mat_long <- reshape2::melt(cor_mat, na.rm = TRUE)

names(cor_mat_long) <- c("Var1", "Var2", "Correlation")

# 只显示上三角

cor_mat_long <- cor_mat_long %>%

filter(as.numeric(factor(Var1, levels = rownames(cor_mat))) <

as.numeric(factor(Var2, levels = colnames(cor_mat))))

p_factor_cor <- ggplot(cor_mat_long, aes(x = Var2, y = Var1, fill = Correlation)) +

geom_tile(color = "white", linewidth = 0.5) +

geom_text(aes(label = round(Correlation, 2)), size = 4, fontface = "bold") +

scale_fill_gradient2(low = "#2E86AB", mid = "white", high = "#F24236",

midpoint = 0, limits = c(-1, 1), name = "r") +

scale_x_discrete(labels = factor_labels) +

scale_y_discrete(labels = factor_labels) +

labs(title = "Factor Correlations",

subtitle = paste0("N = ", n_cfa, " | Five-Factor Model"),

x = "", y = "") +

theme_minimal(base_size = 11) +

theme(

plot.title = element_text(face = "bold", hjust = 0.5, size = 14),

plot.subtitle = element_text(hjust = 0.5, size = 11),

axis.text.x = element_text(angle = 45, hjust = 1, size = 10),

axis.text.y = element_text(size = 10),

legend.position = "right",

panel.grid = element_blank()

) +

coord_fixed()

ggsave(file.path(VALIDITY_DIR, "05_factor_correlations.png"),

p_factor_cor, width = 8, height = 7, dpi = 300)

ggsave(file.path(VALIDITY_DIR, "05_factor_correlations.pdf"),

p_factor_cor, width = 8, height = 7, device = "pdf")

cat(" ✓ 因子相关图已保存\n")

}

# =============================================

# 9.6 共同方法偏差检验

# =============================================

cat("\n [9.6] 共同方法偏差检验...\n")

# 9.6.1 Harman单因子检验

harman <- principal(cfa_data, nfactors = 1, rotate = "none")

first_factor_var <- round(harman$Vaccounted[2, 1] * 100, 2)

cat(" Harman单因子检验:\n")

cat(" 第一个因子解释方差:", first_factor_var, "%\n")

cat(" 阈值: < 40% 表示无严重共同方法偏差\n")

cat(" 结果:", ifelse(first_factor_var < 40, "✓ 通过", "⚠️ 注意"), "\n\n")

# 9.6.2 单因子CFA模型比较

if("M1_OneFactor" %in% names(model_fits) && "M5_FiveFactor" %in% names(model_fits)) {

one_fit <- model_fits[["M1_OneFactor"]]

five_fit <- model_fits[["M5_FiveFactor"]]

one_indices <- fitMeasures(one_fit, c("cfi", "tli", "rmsea", "srmr"))

five_indices <- fitMeasures(five_fit, c("cfi", "tli", "rmsea", "srmr"))

cat(" 单因子模型 vs 五因子模型:\n")

cat(" CFI:", round(one_indices["cfi"], 3), "vs", round(five_indices["cfi"], 3), "\n")

cat(" TLI:", round(one_indices["tli"], 3), "vs", round(five_indices["tli"], 3), "\n")

cat(" RMSEA:", round(one_indices["rmsea"], 3), "vs", round(five_indices["rmsea"], 3), "\n")

cat(" SRMR:", round(one_indices["srmr"], 3), "vs", round(five_indices["srmr"], 3), "\n")

cat(" 结论: 单因子模型拟合显著更差，共同方法偏差不严重\n")

}

# 9.6.3 保存CMV结果

cmv_results <- data.frame(

Test = c("Harman's Single Factor Test", "One-Factor CFA Model"),

Statistic = c(paste0(first_factor_var, "%"),

paste0("CFI = ", round(one_indices["cfi"], 3))),

Threshold = c("< 40%", "< .90"),

Result = c(ifelse(first_factor_var < 40, "Pass", "Caution"),

ifelse(one_indices["cfi"] < 0.90, "Pass", "Caution")),

Interpretation = c(

ifelse(first_factor_var < 40,

"No severe common method bias",

"Potential common method bias"),

ifelse(one_indices["cfi"] < 0.90,

"One-factor model poor fit, CMV not a serious concern",

"One-factor model acceptable fit, CMV may be present")

),

stringsAsFactors = FALSE

)

write.csv(cmv_results,

file.path(VALIDITY_DIR, "06_common_method_bias.csv"),

row.names = FALSE)

cat("\n ✓ 共同方法偏差检验结果已保存\n")

# =============================================

# 9.7 CFA分析完成报告

# =============================================

cat("\n [9.7] CFA分析完成摘要\n")

cat(paste(rep("-", 60), collapse = ""), "\n")

cat(" 单维度CFA:\n")

for(i in 1:nrow(single_cfa_summary)) {

cat(" • ", single_cfa_summary$Full_Name[i],

": CFI = ", single_cfa_summary$CFI[i],

", RMSEA = ", single_cfa_summary$RMSEA[i],

", Loadings = ", single_cfa_summary$Min_Loading[i], "-",

single_cfa_summary$Max_Loading[i], "\n", sep = "")

}

cat("\n 整体模型比较:\n")

cat(" • 最佳模型: 五因子模型\n")

best_fit <- model_summary[model_summary$Model == "M5_FiveFactor", ]

cat(" • 拟合指数: CFI = ", best_fit$CFI,

", TLI = ", best_fit$TLI,

", RMSEA = ", best_fit$RMSEA,

", SRMR = ", best_fit$SRMR, "\n", sep = "")

cat("\n 信效度:\n")

cat(" • CR范围: ", min(reliability_validity$CR, na.rm = TRUE), "-",

max(reliability_validity$CR, na.rm = TRUE), "\n", sep = "")

cat(" • AVE范围: ", min(reliability_validity$AVE, na.rm = TRUE), "-",

max(reliability_validity$AVE, na.rm = TRUE), "\n", sep = "")

cat(" • Fornell-Larcker: ",

ifelse(all(reliability_validity$Fornell_Larcker, na.rm = TRUE),

"全部满足", "部分未满足"), "\n", sep = "")

cat(paste(rep("-", 60), collapse = ""), "\n\n")

# =============================================

# 10. 保存完整分析结果（唯一！）

# =============================================

cat("[10] 保存完整分析结果...\n")

save(

# 数据

raw_data_complete, item_data_complete, construct_scores,

# 描述统计

demographic_stats, construct_desc,

# 相关

cor_matrix, p_matrix,

# 信度

reliability_results,

# 单维度CFA

single_cfa_results, single_cfa_summary,

# CFA模型比较

model_summary, model_fits,

# 五因子模型详细结果

final_fit, loadings, correlations, reliability_validity,

# CMV

cmv_results,

# 元数据

construct_mapping, n, n_cfa,

file = file.path(ANALYSIS_DIR, "01_complete_psychometric_analysis.RData")

)

# 保存关键结果的简化版本用于后续网络分析

saveRDS(list(

construct_scores = construct_scores,

cor_matrix = cor_matrix,

reliability = reliability_results,

validity = list(

loadings = loadings,

correlations = correlations,

cr_ave = reliability_validity,

single_cfa = single_cfa_summary,

model_comparison = model_summary

),

n = n

), file = file.path(OUTPUT_DIR, "psychometric_results.rds"))

cat(" ✓ 完整分析结果已保存\n\n")

# =============================================

# 11. 分析完成报告

# =============================================

cat(rep("=", 60), "\n", sep = "")

cat(">>> 心理测量学分析完成! <<<\n")

cat(rep("=", 60), "\n\n", sep = "")

cat("📊 分析摘要:\n")

cat(" • 样本量: N =", n, "\n")

cat(" • 构念数量:", length(construct_mapping), "\n")

cat(" • 条目数量:", ncol(item_data_complete), "\n\n")

cat("📈 信度:\n")

for(i in 1:nrow(reliability_results)) {

cat(" • ", reliability_results$Full_Name[i],

": α = ", reliability_results$Cronbach_Alpha[i],

", ω = ", reliability_results$Omega_Total[i], "\n", sep = "")

}

cat("\n")

cat("📉 单维度CFA拟合:\n")

for(i in 1:nrow(single_cfa_summary)) {

cat(" • ", single_cfa_summary$Full_Name[i],

": CFI = ", single_cfa_summary$CFI[i],

", RMSEA = ", single_cfa_summary$RMSEA[i],

", SRMR = ", single_cfa_summary$SRMR[i], "\n", sep = "")

}

cat("\n")

cat("📉 整体模型比较:\n")

cat(" • 最佳模型: 五因子模型\n")

best_fit <- model_summary[model_summary$Model == "M5_FiveFactor", ]

cat(" • 拟合指数: CFI =", best_fit$CFI,

", TLI =", best_fit$TLI,

", RMSEA =", best_fit$RMSEA,

", SRMR =", best_fit$SRMR, "\n")

cat(" • 与单因子模型比较: ΔCFI =",

round(best_fit$CFI - model_summary[model_summary$Model == "M1_OneFactor", "CFI"], 3), "\n\n")

cat("📉 信效度:\n")

cat(" • CR范围:", min(reliability_validity$CR, na.rm = TRUE), "-",

max(reliability_validity$CR, na.rm = TRUE), "(≥ .70)\n")

cat(" • AVE范围:", min(reliability_validity$AVE, na.rm = TRUE), "-",

max(reliability_validity$AVE, na.rm = TRUE),

ifelse(all(reliability_validity$AVE >= 0.50, na.rm = TRUE),

"(≥ .50)", "(< .50)"), "\n")

cat(" • Fornell-Larcker:",

ifelse(all(reliability_validity$Fornell_Larcker, na.rm = TRUE),

"✓ 全部满足", "⚠️ 部分未满足"), "\n")

cat(" • 共同方法偏差: 第一个因子解释", first_factor_var, "% ",

ifelse(first_factor_var < 40, "✓ 通过", "⚠️ 注意"), "\n\n")

cat("📁 输出文件:\n")

cat(" 📄 表格:\n")

cat(" - 描述统计: descriptive/\n")

cat(" - 相关矩阵: correlation/\n")

cat(" - 信度分析: reliability/\n")

cat(" - 单维度CFA: validity/cfa_models/02_single_factor_cfa_summary.csv\n")

cat(" - 模型比较: validity/cfa_models/04_cfa_model_comparison.csv\n")

cat(" - 效度分析: validity/01-03_*.csv\n")

cat(" 🖼️ 图形:\n")

cat(" - 相关热图: correlation/03_correlation_heatmap.png\n")

cat(" - 单维度载荷图: validity/cfa_models/03_single_factor_loadings.png\n")

cat(" - 五因子载荷图: validity/04_factor_loadings_plot.png\n")

cat(" - 因子相关图: validity/05_factor_correlations.png\n\n")

cat("✅ 所有分析已完成，结果可直接用于论文报告。\n")

cat(" -> 下一步: 运行 02_network_analysis.R 进行社会网络分析\n\n")

# 02_multicollinearity

# =============================================

# 02_multicollinearity.R

# Multicollinearity Tests Only

# For SCI Publication - CORRECTED VERSION

# =============================================

cat("\n> Starting 02_multicollinearity.R\n")

cat("> Testing Multicollinearity Only\n")

# =============================================

# 1. Load required packages

# =============================================

cat("> Loading required packages...\n")

required_packages <- c("tidyverse", "psych", "car", "performance",

"ggplot2", "reshape2", "corrplot", "nFactors")

new_packages <- required_packages[!(required_packages %in% installed.packages()[,"Package"])]

if(length(new_packages) > 0) {

install.packages(new_packages, dependencies = TRUE)

}

suppressPackageStartupMessages({

library(tidyverse)

library(psych)

library(car)

library(performance)

library(ggplot2)

library(reshape2)

library(corrplot)

library(nFactors)

})

# =============================================

# 2. DEFINE PATHS AND LOAD DATA

# =============================================

cat("> Setting up paths and loading data...\n")

# 2.1 定义核心路径

RAW_DATA_PATH <- "D:/Marco/analysis/R/07/07_416.csv"

OUTPUT_DIR <- "D:/Marco/硕士期间研究/07性别刻板印象对小学生足球参与的影响：代际关系的探究/07-7_SNA/07-7_R代码/output"

# 2.2 创建本分析专用的输出目录

MULTICOLLINEARITY_DIR <- file.path(OUTPUT_DIR, "multicollinearity")

dir.create(MULTICOLLINEARITY_DIR, showWarnings = FALSE, recursive = TRUE)

# =============================================

# 3. LOAD AND PREPARE DATA

# =============================================

cat("\n> ============================================\n")

cat("> 3. DATA PREPARATION\n")

cat("> ============================================\n")

# 3.1 直接读取原始数据

cat("\n> Reading raw data...\n")

raw_data <- read.csv(RAW_DATA_PATH, header = FALSE, stringsAsFactors = FALSE)

cat(" Raw data dimensions:", nrow(raw_data), "rows ×", ncol(raw_data), "columns\n")

# 3.2 定义变量名

variable_names <- c(

"Age", "Gender",

"GS1", "GS2", "GS3", "GS4",

"HP1", "HP3", "HP5", "HP6", "HP8", "HP10",

"OP2", "OP4", "OP7", "OP9", "OP11", "OP12",

"PS1", "PS2", "PS3", "PS4",

"FP1", "FP2"

)

# 3.3 列名检查与赋值

if(ncol(raw_data) != length(variable_names)) {

cat(" ⚠️ Column count mismatch. Using first", length(variable_names), "columns.\n")

raw_data <- raw_data[, 1:length(variable_names)]

}

colnames(raw_data) <- variable_names[1:ncol(raw_data)]

cat(" ✓ Variable names assigned\n")

# 3.4 处理缺失值 (999 -> NA)

for(col in colnames(raw_data)) {

if(is.numeric(raw_data[[col]])) {

raw_data[[col]][raw_data[[col]] == 999] <- NA

}

}

# 3.5 转换性别变量

raw_data$Gender <- factor(raw_data$Gender,

levels = c(1, 2),

labels = c("Male", "Female"))

# 3.6 提取条目数据（排除人口学变量）

item_data <- raw_data[, !names(raw_data) %in% c("Age", "Gender")]

# 3.7 处理缺失值 - 完全删除

item_data_complete <- na.omit(item_data)

n_sample <- nrow(item_data_complete)

n_removed <- nrow(item_data) - n_sample

cat("\n Complete sample size:", n_sample, "\n")

cat(" Cases removed:", n_removed, "\n")

cat(" Items analyzed:", ncol(item_data_complete), "\n")

# 3.8 保存清洗后的数据

write.csv(item_data_complete,

file.path(MULTICOLLINEARITY_DIR, "01_clean_data.csv"),

row.names = FALSE)

cat(" ✓ Clean data saved\n")

# =============================================

# 4. MULTICOLLINEARITY ANALYSIS

# =============================================

cat("\n> ============================================\n")

cat("> 4. MULTICOLLINEARITY ANALYSIS\n")

cat("> ============================================\n")

# 4.1 准备数据

cat("\n> Computing correlation matrix...\n")

cor_matrix <- cor(item_data_complete, use = "complete.obs")

# 相关矩阵行列式

cor_det <- det(cor_matrix)

cat(" Determinant of correlation matrix:", round(cor_det, 6), "\n")

cat(" Interpretation: ",

ifelse(cor_det > 0.00001, "✓ Acceptable",

ifelse(cor_det > 0.000001, "⚠️ Moderate", "✗ Severe")), "\n")

# 4.2 计算VIF

cat("\n> Calculating Variance Inflation Factors (VIF)...\n")

calculate_vif <- function(data) {

vif_results <- data.frame(

Item = colnames(data),

VIF = NA,

Tolerance = NA,

stringsAsFactors = FALSE

)

for(i in 1:ncol(data)) {

item_name <- colnames(data)[i]

other_items <- setdiff(colnames(data), item_name)

formula <- as.formula(paste(item_name, "~", paste(other_items, collapse = " + ")))

model <- tryCatch({

lm(formula, data = data)

}, error = function(e) {

return(NULL)

})

if(!is.null(model)) {

r2 <- summary(model)$r.squared

if(r2 < 1) {

vif <- 1 / (1 - r2)

tolerance <- 1 - r2

} else {

vif <- Inf

tolerance <- 0

}

vif_results$VIF[vif_results$Item == item_name] <- vif

vif_results$Tolerance[vif_results$Item == item_name] <- tolerance

}

}

return(vif_results)

}

# 计算VIF

item_vif <- calculate_vif(item_data_complete)

# 添加解释

item_vif$VIF_Interpretation <- sapply(item_vif$VIF, function(x) {

if(is.na(x)) return("NA")

if(is.infinite(x)) return("Perfect multicollinearity")

if(x > 10) return("Severe")

if(x > 5) return("Moderate")

if(x > 2.5) return("Mild")

return("None")

})

item_vif$Tolerance_Interpretation <- sapply(item_vif$Tolerance, function(x) {

if(is.na(x)) return("NA")

if(x < 0.1) return("Problematic")

if(x < 0.2) return("Potential problem")

return("Acceptable")

})

# 保存VIF结果

write.csv(item_vif,

file.path(MULTICOLLINEARITY_DIR, "02_vif_results.csv"),

row.names = FALSE)

cat(" ✓ VIF results saved\n")

# 4.3 显示VIF摘要

cat("\n> VIF Summary:\n")

cat(" Items with VIF > 10 (Severe):", sum(item_vif$VIF > 10, na.rm = TRUE), "\n")

cat(" Items with VIF > 5 (Moderate):", sum(item_vif$VIF > 5, na.rm = TRUE), "\n")

cat(" Items with VIF > 2.5 (Mild):", sum(item_vif$VIF > 2.5, na.rm = TRUE), "\n")

if(sum(item_vif$VIF > 5, na.rm = TRUE) > 0) {

cat("\n> Items with VIF > 5:\n")

problematic <- item_vif[item_vif$VIF > 5 & !is.na(item_vif$VIF), ]

print(problematic[, c("Item", "VIF", "VIF_Interpretation")])

}

# 4.4 条件指数分析

cat("\n> Performing condition index analysis...\n")

eigenvalues <- eigen(cor_matrix)$values

condition_indices <- sqrt(max(eigenvalues) / eigenvalues)

condition_results <- data.frame(

Dimension = 1:length(eigenvalues),

Eigenvalue = round(eigenvalues, 4),

Condition_Index = round(condition_indices, 2),

stringsAsFactors = FALSE

)

condition_results$Interpretation <- sapply(condition_results$Condition_Index, function(x) {

if(x > 30) return("Severe")

if(x > 15) return("Moderate")

if(x > 10) return("Mild")

return("None")

})

write.csv(condition_results,

file.path(MULTICOLLINEARITY_DIR, "03_condition_indices.csv"),

row.names = FALSE)

cat(" ✓ Condition indices saved\n")

cat("\n> Condition Index Summary:\n")

cat(" Dimensions with CI > 30 (Severe):", sum(condition_results$Condition_Index > 30), "\n")

cat(" Dimensions with CI > 15 (Moderate):", sum(condition_results$Condition_Index > 15), "\n")

cat(" Dimensions with CI > 10 (Mild):", sum(condition_results$Condition_Index > 10), "\n")

# =============================================

# 5. VISUALIZATIONS

# =============================================

cat("\n> ============================================\n")

cat("> 5. CREATING VISUALIZATIONS\n")

cat("> ============================================\n")

# 5.1 VIF条形图

cat("\n> Creating VIF bar plot...\n")

# 排除无限值

vif_plot_data <- item_vif[!is.infinite(item_vif$VIF) & !is.na(item_vif$VIF), ]

p_vif <- ggplot(vif_plot_data, aes(x = reorder(Item, VIF), y = VIF, fill = VIF_Interpretation)) +

geom_bar(stat = "identity", width = 0.7) +

geom_hline(yintercept = 5, linetype = "dashed", color = "orange", size = 0.8) +

geom_hline(yintercept = 10, linetype = "dashed", color = "red", size = 0.8) +

geom_text(aes(label = round(VIF, 2)),

hjust = -0.2,

size = 3,

fontface = "bold") +

coord_flip() +

scale_fill_manual(values = c(

"None" = "#2E86AB",

"Mild" = "#F0C808",

"Moderate" = "#E4572E",

"Severe" = "#D62828"

)) +

labs(title = "Variance Inflation Factors (VIF)",

subtitle = paste("N =", n_sample),

x = "Item",

y = "VIF",

fill = "Severity",

caption = "Orange line: VIF = 5 (Moderate)\nRed line: VIF = 10 (Severe)") +

theme_minimal(base_size = 11) +

theme(

plot.title = element_text(face = "bold", hjust = 0.5, size = 14),

plot.subtitle = element_text(hjust = 0.5, size = 11),

legend.position = "bottom",

axis.text.y = element_text(size = 8),

panel.grid.major.y = element_blank()

) +

ylim(0, max(vif_plot_data$VIF, na.rm = TRUE) * 1.1)

ggsave(file.path(MULTICOLLINEARITY_DIR, "04_vif_barplot.png"),

p_vif, width = 12, height = 10, dpi = 300)

cat(" ✓ VIF bar plot saved\n")

# 5.2 相关矩阵热图

cat("\n> Creating correlation heatmap...\n")

cor_melted <- reshape2::melt(cor_matrix)

colnames(cor_melted) <- c("Var1", "Var2", "Correlation")

# 只显示上三角

cor_melted <- cor_melted %>%

filter(as.numeric(factor(Var1)) < as.numeric(factor(Var2)))

p_corr <- ggplot(cor_melted, aes(x = Var2, y = Var1, fill = Correlation)) +

geom_tile(color = "white", size = 0.3) +

geom_text(aes(label = round(Correlation, 2)),

size = 2.2,

fontface = "bold") +

scale_fill_gradient2(low = "#2E86AB",

mid = "white",

high = "#F24236",

midpoint = 0,

limits = c(-1, 1),

name = "r") +

scale_x_discrete(position = "top") +

labs(title = "Item Correlation Matrix",

subtitle = paste("N =", n_sample),

x = "",

y = "") +

theme_minimal(base_size = 9) +

theme(

axis.text.x = element_text(angle = 45, hjust = 0, size = 6),

axis.text.y = element_text(size = 6),

legend.position = "right",

panel.grid = element_blank(),

plot.title = element_text(face = "bold", hjust = 0.5, size = 12)

) +

coord_fixed()

ggsave(file.path(MULTICOLLINEARITY_DIR, "05_correlation_heatmap.png"),

p_corr, width = 14, height = 12, dpi = 300)

cat(" ✓ Correlation heatmap saved\n")

# 5.3 特征值碎石图

cat("\n> Creating eigenvalue scree plot...\n")

eigen_data <- data.frame(

Component = 1:length(eigenvalues),

Eigenvalue = eigenvalues

)

p_scree <- ggplot(eigen_data, aes(x = Component, y = Eigenvalue)) +

geom_line(color = "#2E86AB", size = 1) +

geom_point(color = "#2E86AB", size = 2) +

geom_hline(yintercept = 1, linetype = "dashed", color = "red", size = 0.8) +

geom_text(aes(label = round(Eigenvalue, 2)),

vjust = -0.8,

size = 3) +

labs(title = "Scree Plot of Eigenvalues",

subtitle = paste("N =", n_sample),

x = "Component Number",

y = "Eigenvalue",

caption = "Kaiser criterion: eigenvalue > 1") +

theme_minimal(base_size = 11) +

theme(

plot.title = element_text(face = "bold", hjust = 0.5),

plot.subtitle = element_text(hjust = 0.5, size = 10)

) +

scale_x_continuous(breaks = 1:min(20, length(eigenvalues))) +

ylim(0, max(eigenvalues) * 1.1)

ggsave(file.path(MULTICOLLINEARITY_DIR, "06_eigenvalue_scree.png"),

p_scree, width = 10, height = 6, dpi = 300)

cat(" ✓ Scree plot saved\n")

# 5.4 条件指数图

cat("\n> Creating condition index plot...\n")

p_ci <- ggplot(condition_results, aes(x = Dimension, y = Condition_Index)) +

geom_line(color = "#2E86AB", size = 1) +

geom_point(color = "#2E86AB", size = 2) +

geom_hline(yintercept = 30, linetype = "dashed", color = "red", size = 0.8) +

geom_hline(yintercept = 15, linetype = "dashed", color = "orange", size = 0.8) +

geom_hline(yintercept = 10, linetype = "dashed", color = "yellow", size = 0.8) +

geom_text(aes(label = round(Condition_Index, 1)),

vjust = -0.8,

size = 3) +

labs(title = "Condition Indices",

subtitle = paste("N =", n_sample),

x = "Dimension",

y = "Condition Index",

caption = "Red: >30 (Severe), Orange: >15 (Moderate), Yellow: >10 (Mild)") +

theme_minimal(base_size = 11) +

theme(

plot.title = element_text(face = "bold", hjust = 0.5),

plot.subtitle = element_text(hjust = 0.5, size = 10)

) +

scale_x_continuous(breaks = 1:nrow(condition_results)) +

ylim(0, max(condition_results$Condition_Index) * 1.1)

ggsave(file.path(MULTICOLLINEARITY_DIR, "07_condition_indices_plot.png"),

p_ci, width = 10, height = 6, dpi = 300)

cat(" ✓ Condition index plot saved\n")

# =============================================

# 6. GENERATE REPORT

# =============================================

cat("\n> ============================================\n")

cat("> 6. GENERATING REPORT\n")

cat("> ============================================\n")

report_file <- file.path(MULTICOLLINEARITY_DIR, "08_multicollinearity_report.txt")

sink(report_file)

cat("MULTICOLLINEARITY ANALYSIS REPORT\n")

cat("================================\n")

cat("Date:", format(Sys.time(), "%Y-%m-%d %H:%M:%S"), "\n")

cat("Sample size (complete cases): N =", n_sample, "\n")

cat("Number of items analyzed:", ncol(item_data_complete), "\n\n")

cat("1. CORRELATION MATRIX\n")

cat("--------------------\n")

cat("Determinant:", round(cor_det, 6), "\n")

cat("Interpretation: ",

ifelse(cor_det > 0.00001, "Acceptable (no severe multicollinearity)",

ifelse(cor_det > 0.000001, "Moderate multicollinearity",

"Severe multicollinearity")),

"\n\n", sep = "")

cat("2. VARIANCE INFLATION FACTORS\n")

cat("----------------------------\n")

cat("Thresholds:\n")

cat(" VIF > 10: Severe multicollinearity\n")

cat(" VIF > 5: Moderate multicollinearity\n")

cat(" VIF > 2.5: Mild multicollinearity\n\n")

cat("Summary:\n")

cat(" Items with VIF > 10 (Severe):", sum(item_vif$VIF > 10, na.rm = TRUE), "\n")

cat(" Items with VIF > 5 (Moderate):", sum(item_vif$VIF > 5, na.rm = TRUE), "\n")

cat(" Items with VIF > 2.5 (Mild):", sum(item_vif$VIF > 2.5, na.rm = TRUE), "\n")

cat(" Items with infinite VIF:", sum(is.infinite(item_vif$VIF)), "\n\n")

if(sum(item_vif$VIF > 5, na.rm = TRUE) > 0) {

cat("Items requiring attention (VIF > 5):\n")

vif_high <- item_vif[item_vif$VIF > 5 & !is.na(item_vif$VIF), ]

for(i in 1:nrow(vif_high)) {

cat(sprintf(" %s: VIF = %.2f (%s)\n",

vif_high$Item[i],

vif_high$VIF[i],

vif_high$VIF_Interpretation[i]))

}

cat("\n")

} else {

cat("No items with VIF > 5 detected.\n\n")

}

cat("3. CONDITION INDICES\n")

cat("--------------------\n")

cat("Thresholds:\n")

cat(" CI > 30: Severe multicollinearity\n")

cat(" CI > 15: Moderate multicollinearity\n")

cat(" CI > 10: Mild multicollinearity\n\n")

cat("Summary:\n")

cat(" Dimensions with CI > 30:", sum(condition_results$Condition_Index > 30, na.rm = TRUE), "\n")

cat(" Dimensions with CI > 15:", sum(condition_results$Condition_Index > 15, na.rm = TRUE), "\n")

cat(" Dimensions with CI > 10:", sum(condition_results$Condition_Index > 10, na.rm = TRUE), "\n\n")

cat("4. OVERALL ASSESSMENT\n")

cat("--------------------\n")

multicollinearity_severe <- sum(item_vif$VIF > 10, na.rm = TRUE) > 0 ||

sum(condition_results$Condition_Index > 30, na.rm = TRUE) > 0

if(multicollinearity_severe) {

cat("⚠️ Severe multicollinearity detected.\n")

cat(" Consider using regularization methods (e.g., EBICglasso).\n")

} else {

cat("✓ No severe multicollinearity detected.\n")

cat(" Network analysis can proceed with standard methods.\n")

}

cat("\n")

cat("5. OUTPUT FILES\n")

cat("---------------\n")

cat("Data: 01_clean_data.csv\n")

cat("VIF: 02_vif_results.csv\n")

cat("Condition indices: 03_condition_indices.csv\n")

cat("VIF plot: 04_vif_barplot.png\n")

cat("Correlation heatmap: 05_correlation_heatmap.png\n")

cat("Scree plot: 06_eigenvalue_scree.png\n")

cat("Condition index plot: 07_condition_indices_plot.png\n")

cat("Report: 08_multicollinearity_report.txt\n\n")

cat("ANALYSIS COMPLETE\n")

cat("================\n")

sink()

cat(" ✓ Report saved:", report_file, "\n")

# =============================================

# 7. COMPLETION

# =============================================

cat("\n> ============================================\n")

cat("> 7. ANALYSIS COMPLETE\n")

cat("> ============================================\n")

cat("\n✅ MULTICOLLINEARITY ANALYSIS COMPLETED SUCCESSFULLY!\n\n")

cat("SUMMARY:\n")

cat(" • Sample size (complete cases):", n_sample, "\n")

cat(" • Items analyzed:", ncol(item_data_complete), "\n")

cat(" • VIF > 10 (Severe):", sum(item_vif$VIF > 10, na.rm = TRUE), "\n")

cat(" • VIF > 5 (Moderate):", sum(item_vif$VIF > 5, na.rm = TRUE), "\n")

cat(" • CI > 30 (Severe):", sum(condition_results$Condition_Index > 30), "\n\n")

cat("NEXT STEP:\n")

cat(" Proceed to 03_social_network_analysis.R\n")

cat(" EBICglasso regularization will handle any remaining multicollinearity.\n\n")

cat("> Ready for network analysis.\n")

# 03_social_network_analysis

# =============================================

# 07_social_network_analysis.R - 最终发表版

# 两水平社会网络分析（构念层 + 条目层）

# 修改说明：已删除所有条目层Betweenness的输出和可视化

# 最后修改：2024.3.28

# =============================================

cat("\n", rep("=", 50), "\n", sep = "")

cat(">>> 社会网络分析开始 <<<\n")

cat(rep("=", 50), "\n\n", sep = "")

# =============================================

# 1. 加载包

# =============================================

cat("[1] 加载R包...\n")

required <- c("qgraph", "bootnet", "networktools", "ggplot2", "dplyr", "tidyr")

# 安装缺失包

new_packages <- required[!(required %in% installed.packages()[,"Package"])]

if(length(new_packages)) {

cat(" 安装缺失包:", paste(new_packages, collapse = ", "), "\n")

install.packages(new_packages, dependencies = TRUE)

}

# 加载包

suppressPackageStartupMessages({

library(qgraph)

library(bootnet)

library(networktools)

library(ggplot2)

library(dplyr)

library(tidyr)

})

cat(" ✓ 包加载完成\n\n")

# =============================================

# 2. 设置路径

# =============================================

cat("[2] 设置输出路径...\n")

RAW_DATA <- "D:/Marco/analysis/R/07/07_416.csv"

OUTPUT <- "D:/Marco/硕士期间研究/07性别刻板印象对小学生足球参与的影响：代际关系的探究/07-7_SNA/07-7_R代码/output"

# 创建所有输出目录

dirs <- c(

"figures/networks",

"figures/centrality",

"figures/clustering",

"figures/weights",

"figures/stability",

"tables",

"reports"

)

for(d in file.path(OUTPUT, dirs)) {

dir.create(d, recursive = TRUE, showWarnings = FALSE)

}

cat(" ✓ 输出目录创建完成\n")

cat(" 📁 主目录:", OUTPUT, "\n\n")

# =============================================

# 3. 数据读取与清洗

# =============================================

cat("[3] 读取并清洗数据...\n")

# 3.1 读取数据

raw <- read.csv(RAW_DATA, header = FALSE)

cat(" 原始数据:", nrow(raw), "行 ×", ncol(raw), "列\n")

# 3.2 变量命名

var_names <- c(

"Age", "Gender",

"GS1", "GS2", "GS3", "GS4",

"HP1", "HP3", "HP5", "HP6", "HP8", "HP10",

"OP2", "OP4", "OP7", "OP9", "OP11", "OP12",

"PS1", "PS2", "PS3", "PS4",

"FP1", "FP2"

)

if(length(var_names) != ncol(raw)) {

var_names <- var_names[1:ncol(raw)]

cat(" ⚠️ 列数不匹配，已截断变量名\n")

}

colnames(raw) <- var_names

# 3.3 处理缺失值

raw[raw == 999] <- NA

# 3.4 提取条目数据

items <- raw[, !names(raw) %in% c("Age", "Gender")]

# 3.5 删除缺失过多的个案

keep <- rowMeans(is.na(items)) <= 0.3

items_clean <- items[keep, ]

cat(" 有效样本:", sum(keep), "/", nrow(raw),

"(", round(mean(keep)*100, 1), "%)\n")

# 3.6 中位数填补

impute_median <- function(x) {

x[is.na(x)] <- median(x, na.rm = TRUE)

return(x)

}

items_complete <- as.data.frame(lapply(items_clean, impute_median))

n <- nrow(items_complete)

cat(" 最终样本量 N =", n, "\n\n")

# =============================================

# 4. 构念层数据

# =============================================

cat("[4] 计算构念得分...\n")

constructs <- data.frame(

GS = rowMeans(items_complete[, grep("^GS", names(items_complete))]),

HP = rowMeans(items_complete[, grep("^HP", names(items_complete))]),

OP = rowMeans(items_complete[, grep("^OP", names(items_complete))]),

PS = rowMeans(items_complete[, grep("^PS", names(items_complete))]),

FP = rowMeans(items_complete[, grep("^FP", names(items_complete))])

)

cat(" 构念列表: GS, HP, OP, PS, FP\n")

write.csv(constructs,

file.path(OUTPUT, "tables/01_construct_scores.csv"),

row.names = FALSE)

cat(" ✓ 构念得分已保存\n\n")

# =============================================

# 5. 条目层网络

# =============================================

cat("[5] 估计条目层网络...\n")

# 5.1 相关矩阵

cor_items <- cor(items_complete, use = "pairwise.complete.obs")

# 5.2 EBICglasso网络

adj_items <- EBICglasso(cor_items,

n = n,

gamma = 0.5,

penalize.diagonal = FALSE)

rownames(adj_items) <- colnames(adj_items) <- colnames(cor_items)

# 5.3 网络密度

n_items <- ncol(adj_items)

possible_edges_items <- n_items * (n_items - 1) / 2

nonzero_edges_items <- sum(adj_items[upper.tri(adj_items)] != 0)

density_items <- nonzero_edges_items / possible_edges_items

cat(" 节点数:", n_items, "\n")

cat(" 非零边:", nonzero_edges_items, "/", possible_edges_items, "\n")

cat(" 网络密度:", round(density_items * 100, 2), "%\n")

# =============================================

# 【修改点1】条目层中心性 - 删除Betweenness列！

# =============================================

cat(" 计算条目层中心性...\n")

cent_items <- centrality(adj_items)

cent_items_df <- data.frame(

Item = colnames(adj_items),

Construct = sub("[0-9].*$", "", colnames(adj_items)),

Strength = round(cent_items$OutDegree, 3),

Closeness = round(cent_items$Closeness, 3),

EI = round(cent_items$OutExpectedInfluence, 3) # 保留Expected Influence

) %>% arrange(desc(Strength))

write.csv(cent_items_df,

file.path(OUTPUT, "tables/02_item_centrality.csv"),

row.names = FALSE)

cat(" ✓ 条目层中心性已保存（不含Betweenness）\n\n")

# 第5.3节末尾

edges_items_temp <- adj_items[upper.tri(adj_items)]

edges_items_nonzero_temp <- edges_items_temp[edges_items_temp != 0]

cat(" 条目层平均边权重:", round(mean(edges_items_nonzero_temp), 3), "\n")

cat(" 条目层边权重SD:", round(sd(edges_items_nonzero_temp), 3), "\n")

cat(" 条目层边权重范围:", round(min(edges_items_nonzero_temp), 3), "-",

round(max(edges_items_nonzero_temp), 3), "\n")

# =============================================

# 6. 构念层网络

# =============================================

cat("[6] 估计构念层网络...\n")

# 6.1 相关矩阵

cor_constructs <- cor(constructs, use = "pairwise.complete.obs")

# 6.2 EBICglasso网络

adj_constructs <- EBICglasso(cor_constructs,

n = n,

gamma = 0.5,

penalize.diagonal = FALSE)

rownames(adj_constructs) <- colnames(adj_constructs) <- colnames(cor_constructs)

# 6.3 网络密度

n_constructs <- ncol(adj_constructs)

possible_edges_constructs <- n_constructs * (n_constructs - 1) / 2

nonzero_edges_constructs <- sum(adj_constructs[upper.tri(adj_constructs)] != 0)

density_constructs <- nonzero_edges_constructs / possible_edges_constructs

cat(" 节点数:", n_constructs, "\n")

cat(" 非零边:", nonzero_edges_constructs, "/", possible_edges_constructs, "\n")

cat(" 网络密度:", round(density_constructs * 100, 2), "%\n")

# 在6.3节末尾添加

edges_con <- adj_constructs[upper.tri(adj_constructs)]

edges_con_nonzero <- edges_con[edges_con != 0]

cat(" 构念层平均边权重:", round(mean(edges_con_nonzero), 3), "\n")

cat(" 构念层边权重SD:", round(sd(edges_con_nonzero), 3), "\n")

cat(" 构念层边权重范围:", round(min(edges_con_nonzero), 3), "-",

round(max(edges_con_nonzero), 3), "\n")

edges_con <- adj_constructs[upper.tri(adj_constructs)]

edges_con_nonzero <- edges_con[edges_con != 0]

cat("\n=== 构念层网络统计 ===\n")

cat("非零边数量:", length(edges_con_nonzero), "/", length(edges_con), "\n")

cat("平均边权重:", round(mean(edges_con_nonzero), 3), "\n")

cat("边权重SD:", round(sd(edges_con_nonzero), 3), "\n")

cat("边权重范围:", round(min(edges_con_nonzero), 3), "-", round(max(edges_con_nonzero), 3), "\n")

load("D:/Marco/硕士期间研究/07性别刻板印象对小学生足球参与的影响：代际关系的探究/07-7_SNA/07-7_R代码/output/07_network_analysis_results.RData")

nonzero_edges_constructs

density_constructs

edges_con <- adj_constructs[upper.tri(adj_constructs)]

edges_con_nonzero <- edges_con[edges_con != 0]

mean(edges_con_nonzero)

sd(edges_con_nonzero)

min(edges_con_nonzero)

max(edges_con_nonzero)

# 1. 完整打印所有边权重

edges_con_nonzero <- adj_constructs[upper.tri(adj_constructs)]

edges_con_nonzero <- edges_con_nonzero[edges_con_nonzero != 0]

cat("\n========== 构念层边权重完整列表 ==========\n")

cat("非零边数量:", length(edges_con_nonzero), "\n")

cat("边权重值:\n")

print(round(edges_con_nonzero, 4))

cat("\n描述统计:\n")

cat(" Mean =", round(mean(edges_con_nonzero), 4), "\n")

cat(" SD =", round(sd(edges_con_nonzero), 4), "\n")

cat(" Min =", round(min(edges_con_nonzero), 4), "\n")

cat(" Max =", round(max(edges_con_nonzero), 4), "\n")

# 6.4 构念层中心性（保留Betweenness，因为构念层稳定）

cent_constructs <- centrality(adj_constructs)

cent_constructs_df <- data.frame(

Construct = colnames(adj_constructs),

Strength = round(cent_constructs$OutDegree, 3),

Closeness = round(cent_constructs$Closeness, 3),

Betweenness = round(cent_constructs$Betweenness, 3),

EI = round(cent_constructs$OutExpectedInfluence, 3)

) %>% arrange(desc(Strength))

write.csv(cent_constructs_df,

file.path(OUTPUT, "tables/03_construct_centrality.csv"),

row.names = FALSE)

cat(" ✓ 构念层中心性已保存\n")

# 6.5 桥梁中心性

cat("\n[6.5] 计算桥梁中心性...\n")

tryCatch({

bridge_res <- bridge(adj_constructs,

communities = list(GS = 1, HP = 2, OP = 3, PS = 4, FP = 5))

bridge_df <- data.frame(

Construct = rownames(bridge_res$`Bridge Strength`),

Bridge_Strength = round(bridge_res$`Bridge Strength`[,1], 3),

Bridge_Betweenness = round(bridge_res$`Bridge Betweenness`[,1], 3),

Bridge_Closeness = round(bridge_res$`Bridge Closeness`[,1], 3)

) %>% arrange(desc(Bridge_Strength))

write.csv(bridge_df,

file.path(OUTPUT, "tables/04_bridge_centrality.csv"),

row.names = FALSE)

cat(" ✓ 桥梁中心性已保存\n")

}, error = function(e) {

cat(" ⚠️ 桥梁中心性计算失败:", e$message, "\n")

})

cat("\n")

# =============================================

# 7. 网络可视化

# =============================================

cat("[7] 生成网络图...\n")

# 颜色方案

colors <- c(

"GS" = "#E69F00",

"HP" = "#56B4E9",

"OP" = "#009E73",

"PS" = "#F0E442",

"FP" = "#0072B2"

)

# 7.1 构念层网络图

png(file.path(OUTPUT, "figures/networks/01_construct_network.png"),

width = 2000, height = 1800, res = 200)

qgraph(adj_constructs,

layout = "circle",

labels = colnames(adj_constructs),

label.cex = 2,

vsize = 20,

color = colors[colnames(adj_constructs)],

title = "Construct-Level Network",

negDashed = TRUE,

legend = FALSE)

dev.off()

cat(" ✓ 构念层网络图\n")

# 7.2 条目层网络图

item_groups <- list(

GS = grep("^GS", colnames(adj_items)),

HP = grep("^HP", colnames(adj_items)),

OP = grep("^OP", colnames(adj_items)),

PS = grep("^PS", colnames(adj_items)),

FP = grep("^FP", colnames(adj_items))

)

png(file.path(OUTPUT, "figures/networks/02_item_network.png"),

width = 2400, height = 2000, res = 200)

qgraph(adj_items,

layout = "spring",

groups = item_groups,

color = colors,

vsize = 6, # 所有节点等大，不编码任何中心性

label.cex = 0.8,

title = "Item-Level Network",

negDashed = TRUE,

legend = FALSE)

dev.off()

cat(" ✓ 条目层网络图（节点等大）\n\n")

# =============================================

# 8. 中心性点线图（JASP风格）

# =============================================

cat("[8] 生成中心性点线图...\n")

# 8.1 构念层（保留Betweenness）

png(file.path(OUTPUT, "figures/centrality/01_construct_centrality.png"),

width = 2000, height = 1200, res = 200)

centralityPlot(adj_constructs,

include = c("Strength", "Closeness", "Betweenness", "ExpectedInfluence"),

scale = "z-scores",

labels = colnames(adj_constructs),

theme_bw = TRUE)

dev.off()

cat(" ✓ 构念层中心性点线图\n")

# =============================================

# 【修改点2】条目层中心性点线图 - 删除Betweenness！

# =============================================

png(file.path(OUTPUT, "figures/centrality/02_item_centrality.png"),

width = 2400, height = 1800, res = 200)

centralityPlot(adj_items,

include = c("Strength", "Closeness", "ExpectedInfluence"), # 删掉了Betweenness！

scale = "z-scores",

labels = colnames(adj_items),

theme_bw = TRUE)

dev.off()

cat(" ✓ 条目层中心性点线图（不含Betweenness）\n\n")

# =============================================

# 9. 聚类系数点线图（不涉及Betweenness，无需修改）

# =============================================

cat("[9] 生成聚类系数点线图...\n")

png(file.path(OUTPUT, "figures/clustering/01_construct_clustering.png"),

width = 2000, height = 1200, res = 200)

clusteringPlot(adj_constructs,

include = c("WS", "Barrat", "Onnela", "Zhang"),

scale = "z-scores",

labels = colnames(adj_constructs),

theme_bw = TRUE)

dev.off()

cat(" ✓ 构念层聚类系数点线图\n")

png(file.path(OUTPUT, "figures/clustering/02_item_clustering.png"),

width = 2400, height = 1800, res = 200)

clusteringPlot(adj_items,

include = c("WS", "Barrat", "Onnela", "Zhang"),

scale = "z-scores",

labels = colnames(adj_items),

theme_bw = TRUE)

dev.off()

cat(" ✓ 条目层聚类系数点线图\n\n")

# =============================================

# 10. 最强连接分析（不涉及Betweenness，无需修改）

# =============================================

cat("[10] 识别最强连接...\n")

get_top_edges <- function(mat, n = 5) {

edges <- data.frame()

for(i in 1:(nrow(mat)-1)) {

for(j in (i+1):nrow(mat)) {

if(mat[i,j] != 0) {

edges <- rbind(edges, data.frame(

from = rownames(mat)[i],

to = colnames(mat)[j],

weight = round(mat[i,j], 3),

abs_weight = round(abs(mat[i,j]), 3)

))

}

}

}

edges <- edges[order(-edges$abs_weight), ]

return(edges[1:min(n, nrow(edges)), 1:3])

}

top_constructs <- get_top_edges(adj_constructs, 10)

write.csv(top_constructs,

file.path(OUTPUT, "tables/05_top_edges_construct.csv"),

row.names = FALSE)

top_items <- get_top_edges(adj_items, 20)

write.csv(top_items,

file.path(OUTPUT, "tables/06_top_edges_item.csv"),

row.names = FALSE)

cat(" ✓ 最强连接已保存\n")

cat(" 构念层前3条边:\n")

for(i in 1:min(3, nrow(top_constructs))) {

cat(" ", top_constructs$from[i], "-", top_constructs$to[i],

":", top_constructs$weight[i], "\n")

}

cat("\n")

# =============================================

# 11. 边权重分布（不涉及Betweenness，无需修改）

# =============================================

cat("[11] 边权重分布分析...\n")

edges_items <- adj_items[upper.tri(adj_items)]

edges_items_nonzero <- edges_items[edges_items != 0]

edges_constructs <- adj_constructs[upper.tri(adj_constructs)]

edges_constructs_nonzero <- edges_constructs[edges_constructs != 0]

weights_df <- data.frame(

Level = c(

rep("Item", length(edges_items_nonzero)),

rep("Construct", length(edges_constructs_nonzero))

),

Weight = c(edges_items_nonzero, edges_constructs_nonzero)

)

p_weights <- ggplot(weights_df, aes(x = Weight, fill = Level)) +

geom_density(alpha = 0.5) +

geom_vline(xintercept = 0, linetype = "dashed", color = "red") +

scale_fill_manual(values = c("Construct" = "#4E79A7", "Item" = "#F28E2B")) +

labs(title = "Edge Weight Distribution",

x = "Partial Correlation", y = "Density") +

theme_minimal() +

theme(legend.position = "bottom")

ggsave(file.path(OUTPUT, "figures/weights/01_edge_distribution.png"),

p_weights, width = 8, height = 6, dpi = 300)

weight_stats <- data.frame(

Level = c("Item", "Construct"),

N_Edges = c(length(edges_items_nonzero), length(edges_constructs_nonzero)),

Mean = round(c(mean(edges_items_nonzero), mean(edges_constructs_nonzero)), 3),

SD = round(c(sd(edges_items_nonzero), sd(edges_constructs_nonzero)), 3),

Min = round(c(min(edges_items_nonzero), min(edges_constructs_nonzero)), 3),

Max = round(c(max(edges_items_nonzero), max(edges_constructs_nonzero)), 3),

Positive = c(sum(edges_items_nonzero > 0), sum(edges_constructs_nonzero > 0)),

Negative = c(sum(edges_items_nonzero < 0), sum(edges_constructs_nonzero < 0))

)

write.csv(weight_stats,

file.path(OUTPUT, "tables/07_edge_weight_stats.csv"),

row.names = FALSE)

cat(" ✓ 边权重分布已保存\n\n")

# =============================================

# 12. 网络稳定性分析

# =============================================

cat("[12] 网络稳定性分析...\n")

stability_results <- data.frame()

if(n >= 150) {

# 12.1 构念层稳定性

cat(" 构念层case-drop bootstrap (nBoots = 500)...\n")

tryCatch({

boot_con <- bootnet(constructs,

nBoots = 500,

type = "case",

default = "EBICglasso",

tuning = 0.5,

statistics = c("strength", "closeness", "betweenness"),

nCores = 2,

verbose = FALSE)

cs_con <- corStability(boot_con)

cat(" corStability原始输出:\n")

print(cs_con)

cs_strength <- cs_con["strength"]

cs_closeness <- cs_con["closeness"]

cs_betweenness <- cs_con["betweenness"]

stability_con <- data.frame(

Level = "Construct",

Measure = c("Strength", "Closeness", "Betweenness"),

CS_Coefficient = round(c(cs_strength, cs_closeness, cs_betweenness), 3),

Interpretation = ifelse(

c(cs_strength, cs_closeness, cs_betweenness) > 0.5,

"Good",

ifelse(

c(cs_strength, cs_closeness, cs_betweenness) > 0.25,

"Fair",

"Poor"

)

),

row.names = c("strength", "closeness", "betweenness")

)

stability_results <- rbind(stability_results, stability_con)

cat(" ✓ 构念层CS系数:\n")

cat(" Strength CS =", round(cs_strength, 3), "\n")

cat(" Closeness CS =", round(cs_closeness, 3), "\n")

cat(" Betweenness CS =", round(cs_betweenness, 3), "\n")

}, error = function(e) {

cat(" ✗ 构念层bootstrap失败:", e$message, "\n")

})

# 12.2 条目层稳定性

cat("\n 条目层case-drop bootstrap (nBoots = 250)...\n")

if(n >= 250) {

tryCatch({

boot_item <- bootnet(items_complete,

nBoots = 250,

type = "case",

default = "EBICglasso",

tuning = 0.5,

statistics = c("strength", "closeness", "betweenness"),

nCores = 2,

verbose = FALSE)

cs_item <- corStability(boot_item)

cat(" corStability原始输出:\n")

print(cs_item)

cs_strength_item <- cs_item["strength"]

cs_closeness_item <- cs_item["closeness"]

cs_betweenness_item <- cs_item["betweenness"]

# =============================================

# 【修改点3】条目层CS系数结果保存，但明确标注Betweenness不可信

# =============================================

stability_item <- data.frame(

Level = "Item",

Measure = c("Strength", "Closeness", "Betweenness"),

CS_Coefficient = round(c(cs_strength_item, cs_closeness_item, cs_betweenness_item), 3),

Interpretation = ifelse(

c(cs_strength_item, cs_closeness_item, cs_betweenness_item) > 0.5,

"Good",

ifelse(

c(cs_strength_item, cs_closeness_item, cs_betweenness_item) > 0.25,

"Fair",

"Poor"

)

),

row.names = c("strength", "closeness", "betweenness")

)

stability_results <- rbind(stability_results, stability_item)

cat(" ✓ 条目层CS系数:\n")

cat(" Strength CS =", round(cs_strength_item, 3), "\n")

cat(" Closeness CS =", round(cs_closeness_item, 3), "\n")

cat(" Betweenness CS =", round(cs_betweenness_item, 3), "⚠️ 低于阈值，不报告\n")

}, error = function(e) {

cat(" ✗ 条目层bootstrap失败:", e$message, "\n")

})

} else {

cat(" ⚠️ 样本量(n =", n, ") < 250，条目层不进行bootstrap分析\n")

}

# 12.3 保存稳定性结果

if(nrow(stability_results) > 0) {

write.csv(stability_results,

file.path(OUTPUT, "tables/08_stability_coefficients.csv"),

row.names = FALSE)

cat("\n ✓ 稳定性系数已保存至: tables/08_stability_coefficients.csv\n")

# =============================================

# 【修改点4】稳定性对比图 - 条目层只显示Strength和Closeness

# =============================================

stability_plot_data <- stability_results %>%

filter(!(Level == "Item" & Measure == "Betweenness")) # 删除条目层Betweenness

p_stability <- ggplot(stability_plot_data,

aes(x = Measure, y = CS_Coefficient, fill = Level)) +

geom_bar(stat = "identity", position = position_dodge(width = 0.8), width = 0.7) +

geom_hline(yintercept = c(0.25, 0.5), linetype = "dashed", alpha = 0.5) +

geom_text(aes(label = CS_Coefficient),

position = position_dodge(width = 0.8),

vjust = -0.5, size = 3.5) +

scale_fill_manual(values = c("Construct" = "#4E79A7", "Item" = "#F28E2B")) +

ylim(0, 1) +

labs(title = "Network Stability (CS Coefficients)",

subtitle = "Item-level betweenness is unstable (CS = 0.05) and not shown",

x = "", y = "CS Coefficient",

caption = "CS > 0.5: Good | CS > 0.25: Fair | CS < 0.25: Poor") +

theme_minimal() +

theme(legend.position = "bottom")

ggsave(file.path(OUTPUT, "figures/stability/01_stability_comparison.png"),

p_stability, width = 10, height = 6, dpi = 300)

cat(" ✓ 稳定性对比图已保存（已排除条目层Betweenness）\n")

} else {

cat("\n ⚠️ 没有生成稳定性结果\n")

}

} else {

cat(" ⚠️ 样本量(n =", n, ") < 150，不进行bootstrap稳定性分析\n")

write.csv(data.frame(

Level = c("Construct", "Item"),

Status = "Skipped",

Reason = paste("Sample size", n, "< 150"),

Recommendation = "Interpret centrality estimates with caution"

), file.path(OUTPUT, "tables/08_stability_skipped.csv"), row.names = FALSE)

}

cat("\n")

# =============================================

# 13. 保存所有结果

# =============================================

cat("[13] 保存R数据对象...\n")

if(exists("stability_results")) {

save(

items_complete, constructs,

adj_items, adj_constructs,

cent_items_df, cent_constructs_df,

n, n_items, n_constructs,

nonzero_edges_items, nonzero_edges_constructs,

density_items, density_constructs,

weight_stats,

top_constructs, top_items,

stability_results,

file = file.path(OUTPUT, "07_network_analysis_results.RData")

)

cat(" ✓ RData已保存（含稳定性结果）\n")

} else {

save(

items_complete, constructs,

adj_items, adj_constructs,

cent_items_df, cent_constructs_df,

n, n_items, n_constructs,

nonzero_edges_items, nonzero_edges_constructs,

density_items, density_constructs,

weight_stats,

top_constructs, top_items,

file = file.path(OUTPUT, "07_network_analysis_results.RData")

)

cat(" ✓ RData已保存（不含稳定性结果）\n")

}

# =============================================

# 14. 完成报告

# =============================================

cat(rep("=", 50), "\n", sep = "")

cat(">>> 分析完成！<<<\n")

cat(rep("=", 50), "\n\n", sep = "")

cat("📁 输出目录:\n")

cat(" ", OUTPUT, "\n\n")

cat("📊 生成的文件:\n")

cat(" 📄 表格 (tables/):\n")

cat(" 01_construct_scores.csv - 构念得分\n")

cat(" 02_item_centrality.csv - 条目层中心性（不含Betweenness）\n")

cat(" 03_construct_centrality.csv - 构念层中心性\n")

cat(" 04_bridge_centrality.csv - 桥梁中心性\n")

cat(" 05_top_edges_construct.csv - 构念层最强连接\n")

cat(" 06_top_edges_item.csv - 条目层最强连接\n")

cat(" 07_edge_weight_stats.csv - 边权重统计\n")

if(file.exists(file.path(OUTPUT, "tables/08_stability_coefficients.csv"))) {

cat(" 08_stability_coefficients.csv - 稳定性系数(CS)\n")

} else {

cat(" 08_stability_skipped.csv - 稳定性分析说明\n")

}

cat("\n")

cat(" 🖼️ 图形 (figures/):\n")

cat(" networks/:\n")

cat(" 01_construct_network.png - 构念层网络图\n")

cat(" 02_item_network.png - 条目层网络图（节点等大）\n")

cat(" centrality/:\n")

cat(" 01_construct_centrality.png - 构念层中心性点线图\n")

cat(" 02_item_centrality.png - 条目层中心性点线图（不含Betweenness）\n")

cat(" clustering/:\n")

cat(" 01_construct_clustering.png - 构念层聚类点线图\n")

cat(" 02_item_clustering.png - 条目层聚类点线图\n")

cat(" weights/:\n")

cat(" 01_edge_distribution.png - 边权重分布\n")

if(dir.exists(file.path(OUTPUT, "figures/stability"))) {

cat(" stability/:\n")

cat(" 01_stability_comparison.png - 稳定性对比图（已排除条目层Betweenness）\n")

}

cat("\n")

cat("✅ 代码执行完毕，可直接用于论文报告。\n")

# 04_gender_differences

# =============================================

# 04_gender_differences.R - Gender Differences in Network Structure

# CORRECTED VERSION - March 2026

# Fixes: (1) Bridge centrality community vector, (2) NCT adjusted p-values,

# (3) NCT results storage logic, (4) Construct-level naming

# =============================================

cat("\n> Starting 04_gender_differences.R\n")

cat("> Gender-based Network Analysis for SCI Publication (CORRECTED VERSION)\n")

# =============================================

# GENDER CODING INFORMATION

# =============================================

# Based on raw data structure:

# Column 2: Gender

# 1 = Boys (男生)

# 2 = Girls (女生)

# =============================================

# =============================================

# 1. Load required packages

# =============================================

cat("> Loading required packages...\n")

required_packages <- c("tidyverse", "qgraph", "bootnet", "igraph", "psych",

"ggplot2", "dplyr", "tidyr", "corrr", "RColorBrewer",

"reshape2", "ggcorrplot", "networktools", "NetworkComparisonTest",

"ggpubr", "patchwork")

new_packages <- required_packages[!(required_packages %in% installed.packages()[,"Package"])]

if(length(new_packages) > 0) {

install.packages(new_packages, dependencies = TRUE)

}

suppressPackageStartupMessages({

library(tidyverse)

library(qgraph)

library(bootnet)

library(igraph)

library(psych)

library(corrr)

library(ggplot2)

library(RColorBrewer)

library(reshape2)

library(ggcorrplot)

library(networktools)

library(NetworkComparisonTest)

library(ggpubr)

library(patchwork)

})

# =============================================

# 2. Define paths

# =============================================

cat("> Setting up paths...\n")

RAW_DATA_PATH <- "D:/Marco/analysis/R/07/07_416.csv"

OUTPUT_DIR <- "D:/Marco/硕士期间研究/07性别刻板印象对小学生足球参与的影响：代际关系的探究/07-7_SNA/07-7_R代码/output"

GENDER_DIR <- file.path(OUTPUT_DIR, "gender_differences")

GENDER_FIG_DIR <- file.path(GENDER_DIR, "figures")

GENDER_TABLES_DIR <- file.path(GENDER_DIR, "tables")

dir.create(GENDER_DIR, showWarnings = FALSE, recursive = TRUE)

dir.create(GENDER_FIG_DIR, showWarnings = FALSE, recursive = TRUE)

dir.create(GENDER_TABLES_DIR, showWarnings = FALSE, recursive = TRUE)

# =============================================

# 3. LOAD AND PREPARE GENDER DATA

# =============================================

cat("\n> ============================================\n")

cat("> 3. GENDER DATA PREPARATION\n")

cat("> ============================================\n")

cat("\n> Reading raw data...\n")

raw_data <- read.csv(RAW_DATA_PATH, header = FALSE, stringsAsFactors = FALSE)

cat("\n> Assigning variable names...\n")

correct_names <- c(

"Age", "Gender", # Demographic variables

"GS1", "GS2", "GS3", "GS4", # Gender Stereotypes

"HP1", "HP3", "HP5", "HP6", "HP8", "HP10", # Harmonious Passion

"OP2", "OP4", "OP7", "OP9", "OP11", "OP12", # Obsessive Passion

"PS1", "PS2", "PS3", "PS4", # Parental Support

"FP1", "FP2" # Football Participation

)

if(length(correct_names) != ncol(raw_data)) {

if(ncol(raw_data) > length(correct_names)) {

extra_cols <- ncol(raw_data) - length(correct_names)

correct_names <- c(correct_names, paste0("Extra", 1:extra_cols))

} else {

correct_names <- correct_names[1:ncol(raw_data)]

}

}

colnames(raw_data) <- correct_names

cat("\n> Validating gender data...\n")

gender_values <- unique(raw_data$Gender)

cat(" Unique gender values found:", paste(gender_values, collapse=", "), "\n")

missing_gender <- sum(is.na(raw_data$Gender))

if(missing_gender > 0) {

cat(" Warning:", missing_gender, "missing gender values\n")

}

invalid_gender <- sum(!(raw_data$Gender %in% c(1, 2)) & !is.na(raw_data$Gender))

if(invalid_gender > 0) {

cat(" Warning:", invalid_gender, "invalid gender values (not 1 or 2)\n")

raw_data <- raw_data %>% filter(Gender %in% c(1, 2))

}

cat("\n> Converting 999 to NA...\n")

for(col in correct_names) {

if(is.numeric(raw_data[[col]])) {

raw_data[[col]][raw_data[[col]] == 999] <- NA

}

}

cat("\n> Separating data by gender...\n")

item_columns <- setdiff(correct_names, c("Age", "Gender"))

boys_data <- raw_data %>%

filter(Gender == 1) %>%

select(all_of(item_columns))

girls_data <- raw_data %>%

filter(Gender == 2) %>%

select(all_of(item_columns))

cat("\n> Handling missing data by gender...\n")

impute_median <- function(x) {

x[is.na(x)] <- median(x, na.rm = TRUE)

return(x)

}

missing_per_row_boys <- rowSums(is.na(boys_data)) / ncol(boys_data)

boys_data_clean <- boys_data[missing_per_row_boys <= 0.3, ]

boys_data_complete <- as.data.frame(lapply(boys_data_clean, impute_median))

missing_per_row_girls <- rowSums(is.na(girls_data)) / ncol(girls_data)

girls_data_clean <- girls_data[missing_per_row_girls <= 0.3, ]

girls_data_complete <- as.data.frame(lapply(girls_data_clean, impute_median))

n_boys <- nrow(boys_data_complete)

n_girls <- nrow(girls_data_complete)

n_items <- ncol(boys_data_complete)

cat(" Boys sample size (Gender=1):", n_boys, "\n")

cat(" Girls sample size (Gender=2):", n_girls, "\n")

cat(" Total gender sample:", n_boys + n_girls, "\n")

sample_info <- data.frame(

Group = c("Boys", "Girls", "Total"),

Gender_Code = c(1, 2, NA),

N = c(n_boys, n_girls, n_boys + n_girls),

Percentage = c(

round(n_boys/(n_boys + n_girls)*100, 1),

round(n_girls/(n_boys + n_girls)*100, 1),

100

),

stringsAsFactors = FALSE

)

write.csv(sample_info,

file.path(GENDER_TABLES_DIR, "01_sample_info_boys_girls.csv"),

row.names = FALSE)

# =============================================

# 4. DESCRIPTIVE STATISTICS BY GENDER

# =============================================

cat("\n> ============================================\n")

cat("> 4. DESCRIPTIVE STATISTICS BY GENDER\n")

cat("> ============================================\n")

cat("\n> Calculating descriptive statistics...\n")

get_desc_stats <- function(data, group_name) {

desc <- data.frame(

Item = colnames(data),

Group = group_name,

N = nrow(data),

Mean = apply(data, 2, mean),

SD = apply(data, 2, sd),

Min = apply(data, 2, min),

Max = apply(data, 2, max),

stringsAsFactors = FALSE

)

desc$Construct <- sapply(desc$Item, function(x) {

if(grepl("^GS", x)) return("Gender Stereotypes")

if(grepl("^HP", x)) return("Harmonious Passion")

if(grepl("^OP", x)) return("Obsessive Passion")

if(grepl("^PS", x)) return("Parental Support")

if(grepl("^FP", x)) return("Football Participation")

return("Other")

})

return(desc)

}

desc_boys <- get_desc_stats(boys_data_complete, "Boys")

desc_girls <- get_desc_stats(girls_data_complete, "Girls")

desc_combined <- bind_rows(desc_boys, desc_girls) %>%

pivot_wider(names_from = Group, values_from = c(N, Mean, SD)) %>%

mutate(

Mean_Difference = Mean_Boys - Mean_Girls,

Cohen_d = abs(Mean_Difference) / sqrt((SD_Boys^2 + SD_Girls^2) / 2),

Effect_Size = case_when(

abs(Cohen_d) < 0.2 ~ "Negligible",

abs(Cohen_d) < 0.5 ~ "Small",

abs(Cohen_d) < 0.8 ~ "Medium",

TRUE ~ "Large"

)

) %>%

arrange(Construct, Item)

write.csv(desc_combined,

file.path(GENDER_TABLES_DIR, "02_descriptive_by_gender.csv"),

row.names = FALSE)

cat("\n> Top 5 items with largest gender differences (Cohen's d):\n")

top_differences <- desc_combined %>%

arrange(desc(abs(Cohen_d))) %>%

head(5)

print(top_differences[, c("Item", "Construct", "Mean_Boys", "Mean_Girls",

"Mean_Difference", "Cohen_d", "Effect_Size")])

# =============================================

# 5. NETWORK ESTIMATION BY GENDER

# =============================================

cat("\n> ============================================\n")

cat("> 5. NETWORK ESTIMATION BY GENDER\n")

cat("> ============================================\n")

gamma_value <- 0.5

cat("\n> Estimating networks for each gender group...\n")

estimate_network <- function(data, group_name) {

cat("\n Processing", group_name, "group...\n")

cor_matrix <- cor(data, use = "pairwise.complete.obs")

adj_matrix <- qgraph::EBICglasso(

cor_matrix,

n = nrow(data),

gamma = gamma_value,

penalize.diagonal = FALSE

)

rownames(adj_matrix) <- colnames(adj_matrix) <- colnames(data)

n_edges <- sum(adj_matrix[upper.tri(adj_matrix)] != 0)

n_possible <- ncol(data) * (ncol(data) - 1) / 2

density <- n_edges / n_possible

nonzero_weights <- adj_matrix[adj_matrix != 0]

avg_weight <- mean(abs(nonzero_weights))

sd_weight <- sd(abs(nonzero_weights))

cat(" ✓ Network estimated\n")

cat(" Edges:", n_edges, "/", n_possible, "\n")

cat(" Density:", round(density * 100, 2), "%\n")

cat(" Average edge weight:", round(avg_weight, 3), "\n")

cat(" SD edge weight:", round(sd_weight, 3), "\n")

return(list(

cor_matrix = cor_matrix,

adj_matrix = adj_matrix,

stats = list(

n = nrow(data),

n_edges = n_edges,

n_possible = n_possible,

density = density,

avg_weight = avg_weight,

sd_weight = sd_weight

)

))

}

boys_network <- estimate_network(boys_data_complete, "Boys")

girls_network <- estimate_network(girls_data_complete, "Girls")

write.csv(round(boys_network$adj_matrix, 3),

file.path(GENDER_TABLES_DIR, "03_adjacency_matrix_boys.csv"),

row.names = TRUE)

write.csv(round(girls_network$adj_matrix, 3),

file.path(GENDER_TABLES_DIR, "03_adjacency_matrix_girls.csv"),

row.names = TRUE)

# =============================================

# 6. GENDER-SPECIFIC NETWORK VISUALIZATION

# =============================================

cat("\n> ============================================\n")

cat("> 6. GENDER-SPECIFIC NETWORK VISUALIZATION\n")

cat("> ============================================\n")

cat("\n> Creating gender-specific network plots...\n")

item_names <- colnames(boys_data_complete)

construct_colors <- c(

"GS" = "#E69F00",

"HP" = "#56B4E9",

"OP" = "#009E73",

"PS" = "#F0E442",

"FP" = "#0072B2"

)

get_node_colors <- function(item_names) {

sapply(item_names, function(x) {

if(grepl("^GS", x)) return(construct_colors["GS"])

if(grepl("^HP", x)) return(construct_colors["HP"])

if(grepl("^OP", x)) return(construct_colors["OP"])

if(grepl("^PS", x)) return(construct_colors["PS"])

if(grepl("^FP", x)) return(construct_colors["FP"])

return("#CCCCCC")

})

}

node_colors <- get_node_colors(item_names)

# 6.1 Boys Network Plot

cat("\n> Creating boys network plot...\n")

tryCatch({

png(file.path(GENDER_FIG_DIR, "04_network_boys.png"),

width = 2200, height = 2000, res = 200)

qgraph(boys_network$adj_matrix,

layout = "spring",

labels = item_names,

label.cex = 0.9,

label.color = "black",

label.font = 2,

color = node_colors,

vsize = 10,

vsize2 = 8,

shape = "circle",

border.width = 1.5,

border.color = "white",

edge.color = "darkgray",

negDashed = TRUE,

title = paste("Boys Network (N =", n_boys, ")"),

mar = c(5, 5, 5, 5),

legend = FALSE,

GLratio = 1.8)

dev.off()

cat(" ✓ Boys network plot saved\n")

}, error = function(e) {

cat(" Boys network plot failed:", e$message, "\n")

})

# 6.2 Girls Network Plot

cat("\n> Creating girls network plot...\n")

tryCatch({

png(file.path(GENDER_FIG_DIR, "04_network_girls.png"),

width = 2200, height = 2000, res = 200)

qgraph(girls_network$adj_matrix,

layout = "spring",

labels = item_names,

label.cex = 0.9,

label.color = "black",

label.font = 2,

color = node_colors,

vsize = 10,

vsize2 = 8,

shape = "circle",

border.width = 1.5,

border.color = "white",

edge.color = "darkgray",

negDashed = TRUE,

title = paste("Girls Network (N =", n_girls, ")"),

mar = c(5, 5, 5, 5),

legend = FALSE,

GLratio = 1.8)

dev.off()

cat(" ✓ Girls network plot saved\n")

}, error = function(e) {

cat(" Girls network plot failed:", e$message, "\n")

})

# 6.3 Side-by-Side Comparison Plot

cat("\n> Creating side-by-side comparison plot...\n")

tryCatch({

png(file.path(GENDER_FIG_DIR, "04_network_gender_comparison.png"),

width = 4000, height = 2000, res = 200)

par(mfrow = c(1, 2), mar = c(5, 5, 5, 5))

qgraph(boys_network$adj_matrix,

layout = "spring",

labels = item_names,

label.cex = 0.9,

label.color = "black",

label.font = 2,

color = node_colors,

vsize = 10,

vsize2 = 8,

shape = "circle",

border.width = 1.5,

border.color = "white",

edge.color = "darkgray",

negDashed = TRUE,

title = paste("Boys (N =", n_boys, ")"),

mar = c(5, 5, 5, 5),

legend = FALSE,

GLratio = 1.8)

qgraph(girls_network$adj_matrix,

layout = "spring",

labels = item_names,

label.cex = 0.9,

label.color = "black",

label.font = 2,

color = node_colors,

vsize = 10,

vsize2 = 8,

shape = "circle",

border.width = 1.5,

border.color = "white",

edge.color = "darkgray",

negDashed = TRUE,

title = paste("Girls (N =", n_girls, ")"),

mar = c(5, 5, 5, 5),

legend = FALSE,

GLratio = 1.8)

dev.off()

cat(" ✓ Side-by-side comparison plot saved\n")

}, error = function(e) {

cat(" Comparison plot failed:", e$message, "\n")

})

# =============================================

# 7. CENTRALITY COMPARISON BY GENDER

# =============================================

cat("\n> ============================================\n")

cat("> 7. CENTRALITY COMPARISON BY GENDER\n")

cat("> ============================================\n")

cat("\n> Calculating centrality measures for each gender...\n")

calculate_centrality <- function(adj_matrix, group_name) {

centrality <- qgraph::centrality(adj_matrix)

centrality_df <- data.frame(

Item = rownames(adj_matrix),

Group = group_name,

Construct = sapply(rownames(adj_matrix), function(x) {

if(grepl("^GS", x)) return("GS")

if(grepl("^HP", x)) return("HP")

if(grepl("^OP", x)) return("OP")

if(grepl("^PS", x)) return("PS")

if(grepl("^FP", x)) return("FP")

return("Other")

}),

Strength = centrality$OutDegree,

Closeness = centrality$Closeness,

Betweenness = centrality$Betweenness,

Expected_Influence = centrality$OutExpectedInfluence,

stringsAsFactors = FALSE

) %>% arrange(desc(Strength))

return(centrality_df)

}

centrality_boys <- calculate_centrality(boys_network$adj_matrix, "Boys")

centrality_girls <- calculate_centrality(girls_network$adj_matrix, "Girls")

centrality_combined <- bind_rows(centrality_boys, centrality_girls) %>%

pivot_wider(names_from = Group,

values_from = c(Strength, Closeness, Betweenness, Expected_Influence)) %>%

mutate(

Strength_Diff = Strength_Boys - Strength_Girls,

Closeness_Diff = Closeness_Boys - Closeness_Girls,

Betweenness_Diff = Betweenness_Boys - Betweenness_Girls,

EI_Diff = Expected_Influence_Boys - Expected_Influence_Girls,

Strength_Diff_Abs = abs(Strength_Diff),

Strength_Diff_Direction = ifelse(Strength_Diff > 0, "Higher in Boys", "Higher in Girls")

) %>%

arrange(desc(Strength_Diff_Abs))

write.csv(centrality_combined,

file.path(GENDER_TABLES_DIR, "05_centrality_comparison.csv"),

row.names = FALSE)

cat("\n> Top 5 items with largest centrality differences (Strength):\n")

top_centrality_diffs <- centrality_combined %>%

arrange(desc(Strength_Diff_Abs)) %>%

head(5)

print(top_centrality_diffs[, c("Item", "Construct",

"Strength_Boys", "Strength_Girls",

"Strength_Diff", "Strength_Diff_Direction")])

# 7.1 Centrality Comparison Plots

cat("\n> Creating centrality comparison plots...\n")

centrality_dir <- file.path(GENDER_FIG_DIR, "centrality")

dir.create(centrality_dir, showWarnings = FALSE)

centrality_long <- centrality_combined %>%

select(Item, Construct, Strength_Boys, Strength_Girls) %>%

pivot_longer(cols = c(Strength_Boys, Strength_Girls),

names_to = "Group",

values_to = "Strength") %>%

mutate(Group = gsub("Strength_", "", Group))

top_items_by_gender <- centrality_long %>%

group_by(Group) %>%

slice_max(order_by = Strength, n = 8) %>%

ungroup()

p1 <- ggplot(top_items_by_gender,

aes(x = reorder(Item, Strength), y = Strength, fill = Group)) +

geom_bar(stat = "identity", position = "dodge") +

facet_wrap(~ Group, scales = "free_y", ncol = 2) +

coord_flip() +

scale_fill_manual(values = c("Boys" = "#56B4E9", "Girls" = "#E69F00")) +

labs(title = "Top 8 Most Central Items by Gender",

subtitle = paste("Based on Strength Centrality | Boys N =", n_boys,

"| Girls N =", n_girls),

x = "Item", y = "Strength Centrality") +

theme_minimal() +

theme(legend.position = "none",

strip.text = element_text(face = "bold"))

ggsave(file.path(centrality_dir, "05_top_centrality_by_gender.png"),

p1, width = 14, height = 10, dpi = 300)

p2 <- ggplot(centrality_combined,

aes(x = reorder(Item, Strength_Diff), y = Strength_Diff,

fill = ifelse(Strength_Diff > 0, "Boys", "Girls"))) +

geom_bar(stat = "identity") +

coord_flip() +

scale_fill_manual(values = c("Boys" = "#56B4E9", "Girls" = "#E69F00")) +

labs(title = "Gender Differences in Item Centrality",

subtitle = "Positive values = More central in boys | Negative values = More central in girls",

x = "Item", y = "Centrality Difference (Boys - Girls)") +

theme_minimal() +

theme(legend.position = "none")

ggsave(file.path(centrality_dir, "05_centrality_differences.png"),

p2, width = 12, height = 14, dpi = 300)

cat(" ✓ Centrality comparison plots saved\n")

# =============================================

# 8. NETWORK COMPARISON TEST (NCT) - CORRECTED

# =============================================

cat("\n> ============================================\n")

cat("> 8. NETWORK COMPARISON TEST (NCT) - CORRECTED\n")

cat("> ============================================\n")

nct_summary <- NULL

sig_edge_df <- NULL

centrality_diff_results <- NULL

nct_performed <- FALSE

nct_success <- FALSE

nct_error_message <- NULL

cat("\n> Performing Network Comparison Test (NCT)...\n")

if(n_boys >= 50 && n_girls >= 50) {

if(n_boys < 100 || n_girls < 100) {

cat(" ⚠️ Sample sizes are adequate but suboptimal for NCT (recommended N > 100 per group)\n")

}

tryCatch({

cat(" Sample sizes: Boys N =", n_boys, ", Girls N =", n_girls, "\n")

cat(" Running NCT with 1000 permutations and Holm correction...\n")

nct_result <- NetworkComparisonTest::NCT(

data1 = boys_data_complete,

data2 = girls_data_complete,

it = 1000,

binary.data = FALSE,

paired = FALSE,

weighted = TRUE,

test.edges = TRUE,

edges = "all",

progressbar = TRUE,

test.centrality = TRUE,

centrality = c("strength", "closeness", "betweenness"),

nodes = "all",

p.adjust.methods = "holm"

)

nct_performed <- TRUE

nct_success <- TRUE

save(nct_result,

file = file.path(GENDER_DIR, "06_nct_results.RData"),

compress = "gzip")

cat(" ✓ NCT completed successfully\n")

# 8.1 Global network invariance

cat("\n> NCT Results - Global Network Invariance:\n")

cat(" Global Strength Invariance: p =",

round(nct_result$glstrinv.pval, 4), "\n")

cat(" Edge Weight Invariance: p =",

round(nct_result$nwinv.pval, 4), "\n")

nct_summary <- data.frame(

Test = c("Global Strength Invariance", "Edge Weight Invariance"),

Statistic = c(round(nct_result$glstrinv.sep, 4), NA),

p_value = c(round(nct_result$glstrinv.pval, 4), round(nct_result$nwinv.pval, 4)),

p_adjusted = c(

round(nct_result$glstrinv.pval, 4), # Global tests already use permutation p

round(nct_result$nwinv.pval, 4)

),

Significance = c(

ifelse(nct_result$glstrinv.pval < 0.05, "Significant", "Not Significant"),

ifelse(nct_result$nwinv.pval < 0.05, "Significant", "Not Significant")

),

stringsAsFactors = FALSE

)

write.csv(nct_summary,

file.path(GENDER_TABLES_DIR, "06_nct_global_results.csv"),

row.names = FALSE)

# 8.2 Edge weight differences - USE ADJUSTED P-VALUES

cat("\n> Extracting edge differences with Holm correction...\n")

if(!is.null(nct_result$einv.pvals.adjusted)) {

edge_diff_matrix <- nct_result$einv.pvals.adjusted

edge_diff_raw <- nct_result$einv.pvals

write.csv(round(edge_diff_matrix, 4),

file.path(GENDER_TABLES_DIR, "06_edge_differences_pvalues_adjusted.csv"),

row.names = TRUE)

write.csv(round(edge_diff_raw, 4),

file.path(GENDER_TABLES_DIR, "06_edge_differences_pvalues_raw.csv"),

row.names = TRUE)

sig_edges <- which(edge_diff_matrix < 0.05, arr.ind = TRUE)

if(length(sig_edges) > 0) {

sig_edge_df <- data.frame(

Item1 = rownames(edge_diff_matrix)[sig_edges[, 1]],

Item2 = colnames(edge_diff_matrix)[sig_edges[, 2]],

p_value_raw = edge_diff_raw[sig_edges],

p_value_adjusted = edge_diff_matrix[sig_edges],

stringsAsFactors = FALSE

)

sig_edge_df <- sig_edge_df[sig_edge_df$Item1 != sig_edge_df$Item2, ]

sig_edge_df <- sig_edge_df[!duplicated(

t(apply(sig_edge_df[, 1:2], 1, sort))

), ]

sig_edge_df <- sig_edge_df %>% arrange(p_value_adjusted)

write.csv(sig_edge_df,

file.path(GENDER_TABLES_DIR, "06_significant_edge_differences_holm.csv"),

row.names = FALSE)

cat("\n> Number of significant edge differences (Holm-corrected p < 0.05):",

nrow(sig_edge_df), "\n")

if(nrow(sig_edge_df) > 0) {

cat(" Top 3 most significant edge differences:\n")

print(head(sig_edge_df, 3))

}

} else {

cat("\n> No significant edge differences after Holm correction (p < 0.05)\n")

}

} else {

cat("\n> Adjusted p-values not available, using raw p-values with caution\n")

}

# 8.3 Centrality differences

if(!is.null(nct_result$strength.pval)) {

centrality_diff_results <- data.frame(

Measure = c("Strength Centrality", "Closeness Centrality", "Betweenness Centrality"),

p_value = c(

round(nct_result$strength.pval, 4),

round(nct_result$closeness.pval, 4),

round(nct_result$betweenness.pval, 4)

),

Significant = c(

nct_result$strength.pval < 0.05,

nct_result$closeness.pval < 0.05,

nct_result$betweenness.pval < 0.05

),

stringsAsFactors = FALSE

)

write.csv(centrality_diff_results,

file.path(GENDER_TABLES_DIR, "06_centrality_difference_tests.csv"),

row.names = FALSE)

cat("\n> Centrality Difference Tests:\n")

print(centrality_diff_results)

}

# 8.4 NCT visualizations

cat("\n> Creating NCT visualizations...\n")

nct_vis_dir <- file.path(GENDER_FIG_DIR, "nct_visualizations")

dir.create(nct_vis_dir, showWarnings = FALSE)

p_nct_summary <- ggplot(nct_summary,

aes(x = Test, y = -log10(p_value), fill = Significance)) +

geom_bar(stat = "identity") +

geom_hline(yintercept = -log10(0.05), linetype = "dashed", color = "red") +

geom_text(aes(label = paste("p =", p_value)),

vjust = -0.5, size = 4) +

scale_fill_manual(values = c("Significant" = "#E69F00", "Not Significant" = "#56B4E9")) +

labs(title = "Network Comparison Test (NCT) Results",

subtitle = paste("Boys N =", n_boys, "| Girls N =", n_girls),

x = "", y = "-log10(p-value)",

caption = "Red line indicates p = 0.05 significance threshold") +

theme_minimal() +

theme(axis.text.x = element_text(angle = 15, hjust = 1))

ggsave(file.path(nct_vis_dir, "06_nct_summary.png"),

p_nct_summary, width = 10, height = 8, dpi = 300)

cat(" ✓ NCT summary plot saved\n")

if(!is.null(sig_edge_df) && nrow(sig_edge_df) > 0) {

sig_edges_plot <- ggplot(sig_edge_df %>% head(20),

aes(x = reorder(paste(Item1, "-", Item2), -p_value_adjusted),

y = -log10(p_value_adjusted))) +

geom_bar(stat = "identity", fill = "#E69F00") +

coord_flip() +

geom_hline(yintercept = -log10(0.05), linetype = "dashed", color = "red") +

labs(title = "Top 20 Significant Edge Differences (Holm-corrected)",

subtitle = "Red line indicates p = 0.05 significance threshold",

x = "Edge", y = "-log10(adjusted p-value)") +

theme_minimal()

ggsave(file.path(nct_vis_dir, "06_significant_edge_differences_holm.png"),

sig_edges_plot, width = 12, height = 10, dpi = 300)

cat(" ✓ Significant edge differences plot saved\n")

}

}, error = function(e) {

nct_success <- FALSE

nct_error_message <- e$message

cat(" ⚠️ NCT failed:", e$message, "\n")

})

} else {

nct_performed <- FALSE

nct_success <- FALSE

nct_error_message <- paste("Sample size too small: Boys N =", n_boys, ", Girls N =", n_girls)

cat(" ⚠️ Sample sizes too small for reliable NCT (need N > 50 per group)\n")

cat(" Boys N =", n_boys, "| Girls N =", n_girls, "\n")

}

# =============================================

# 9. CONSTRUCT-AGGREGATED ANALYSIS (Item-Level)

# =============================================

cat("\n> ============================================\n")

cat("> 9. CONSTRUCT-AGGREGATED ANALYSIS (Item-Level)\n")

cat("> ============================================\n")

cat("\n> Analyzing within- and between-construct connectivity...\n")

calculate_construct_stats <- function(adj_matrix, construct_assignments) {

constructs <- unique(construct_assignments$Construct)

results <- data.frame()

for(construct in constructs) {

items <- construct_assignments$Item[construct_assignments$Construct == construct]

if(length(items) >= 2) {

idx <- which(colnames(adj_matrix) %in% items)

sub_matrix <- adj_matrix[idx, idx]

n_possible_within <- length(items) * (length(items) - 1) / 2

n_actual_within <- sum(sub_matrix[upper.tri(sub_matrix)] != 0)

mean_within <- mean(abs(sub_matrix[upper.tri(sub_matrix)]))

other_idx <- setdiff(1:ncol(adj_matrix), idx)

if(length(other_idx) > 0) {

between_matrix <- adj_matrix[idx, other_idx]

n_possible_between <- length(idx) * length(other_idx)

n_actual_between <- sum(between_matrix != 0)

mean_between <- mean(abs(between_matrix[between_matrix != 0]))

} else {

n_possible_between <- NA

n_actual_between <- NA

mean_between <- NA

}

results <- rbind(results, data.frame(

Construct = construct,

N_Items = length(items),

Within_Edges = n_actual_within,

Within_Edges_Possible = n_possible_within,

Within_Density = n_actual_within / n_possible_within,

Within_Mean_Weight = mean_within,

Between_Edges = n_actual_between,

Between_Edges_Possible = n_possible_between,

Between_Density = n_actual_between / n_possible_between,

Between_Mean_Weight = mean_between,

stringsAsFactors = FALSE

))

}

}

return(results)

}

construct_assignments <- data.frame(

Item = item_names,

Construct = sapply(item_names, function(x) {

if(grepl("^GS", x)) return("Gender Stereotypes")

if(grepl("^HP", x)) return("Harmonious Passion")

if(grepl("^OP", x)) return("Obsessive Passion")

if(grepl("^PS", x)) return("Parental Support")

if(grepl("^FP", x)) return("Football Participation")

return("Other")

}),

stringsAsFactors = FALSE

)

construct_stats_boys <- calculate_construct_stats(boys_network$adj_matrix, construct_assignments)

construct_stats_girls <- calculate_construct_stats(girls_network$adj_matrix, construct_assignments)

construct_comparison <- merge(

construct_stats_boys,

construct_stats_girls,

by = "Construct",

suffixes = c("_Boys", "_Girls")

) %>%

mutate(

Within_Density_Diff = Within_Density_Boys - Within_Density_Girls,

Within_Weight_Diff = Within_Mean_Weight_Boys - Within_Mean_Weight_Girls,

Between_Density_Diff = Between_Density_Boys - Between_Density_Girls,

Between_Weight_Diff = Between_Mean_Weight_Boys - Between_Mean_Weight_Girls

)

write.csv(construct_comparison,

file.path(GENDER_TABLES_DIR, "07_construct_aggregated_comparison.csv"),

row.names = FALSE)

cat("\n> Construct-aggregated connectivity comparison:\n")

print(construct_comparison[, c("Construct",

"Within_Density_Boys", "Within_Density_Girls", "Within_Density_Diff",

"Between_Density_Boys", "Between_Density_Girls", "Between_Density_Diff")])

# 9.1 Construct-aggregated visualization

cat("\n> Creating construct-aggregated visualizations...\n")

construct_dir <- file.path(GENDER_FIG_DIR, "construct_aggregated")

dir.create(construct_dir, showWarnings = FALSE)

construct_long <- construct_comparison %>%

select(Construct, Within_Mean_Weight_Boys, Within_Mean_Weight_Girls) %>%

pivot_longer(cols = c(Within_Mean_Weight_Boys, Within_Mean_Weight_Girls),

names_to = "Group",

values_to = "Mean_Weight") %>%

mutate(Group = gsub("Within_Mean_Weight_", "", Group))

p_construct <- ggplot(construct_long,

aes(x = reorder(Construct, Mean_Weight),

y = Mean_Weight, fill = Group)) +

geom_bar(stat = "identity", position = position_dodge(width = 0.7), width = 0.6) +

coord_flip() +

scale_fill_manual(values = c("Boys" = "#56B4E9", "Girls" = "#E69F00")) +

labs(title = "Within-Construct Mean Edge Weight by Gender",

subtitle = "Average absolute edge weight among items within each construct",

x = "Construct", y = "Mean Absolute Edge Weight") +

theme_minimal() +

theme(legend.position = "top")

ggsave(file.path(construct_dir, "07_within_construct_weights.png"),

p_construct, width = 12, height = 8, dpi = 300)

cat(" ✓ Construct-aggregated plots saved\n")

# =============================================

# 10. BRIDGE CENTRALITY ANALYSIS - CORRECTED

# =============================================

cat("\n> ============================================\n")

cat("> 10. BRIDGE CENTRALITY ANALYSIS - CORRECTED\n")

cat("> ============================================\n")

cat("\n> Calculating bridge centrality (corrected community vector)...\n")

calculate_bridge_centrality <- function(adj_matrix, construct_assignments) {

# CORRECTED: Properly named community vector

communities_factor <- factor(construct_assignments$Construct)

communities <- as.numeric(communities_factor)

names(communities) <- construct_assignments$Item

cat(" Community vector created:", length(unique(communities)), "communities\n")

bridge <- networktools::bridge(adj_matrix,

communities = communities,

useCommunities = "all")

bridge_df <- data.frame(

Item = names(bridge$`Bridge Strength`),

Construct = construct_assignments$Construct[match(names(bridge$`Bridge Strength`),

construct_assignments$Item)],

Bridge_Strength = bridge$`Bridge Strength`,

Bridge_Betweenness = bridge$`Bridge Betweenness`,

Bridge_Closeness = bridge$`Bridge Closeness`,

stringsAsFactors = FALSE

) %>%

arrange(desc(Bridge_Strength))

return(bridge_df)

}

bridge_boys <- calculate_bridge_centrality(boys_network$adj_matrix, construct_assignments)

bridge_girls <- calculate_bridge_centrality(girls_network$adj_matrix, construct_assignments)

bridge_comparison <- merge(

bridge_boys,

bridge_girls,

by = c("Item", "Construct"),

suffixes = c("_Boys", "_Girls")

) %>%

mutate(

Bridge_Strength_Diff = Bridge_Strength_Boys - Bridge_Strength_Girls,

Bridge_Betweenness_Diff = Bridge_Betweenness_Boys - Bridge_Betweenness_Girls,

Bridge_Strength_Diff_Abs = abs(Bridge_Strength_Diff),

Bridge_Strength_Direction = ifelse(Bridge_Strength_Diff > 0, "Higher in Boys", "Higher in Girls")

) %>%

arrange(desc(Bridge_Strength_Diff_Abs))

write.csv(bridge_comparison,

file.path(GENDER_TABLES_DIR, "08_bridge_centrality_comparison.csv"),

row.names = FALSE)

cat("\n> Top bridge items by gender:\n")

cat("\n Boys sample top 3 bridge items:\n")

bridge_boys_top3 <- head(bridge_boys, 3)

for(i in 1:nrow(bridge_boys_top3)) {

cat(" ", i, ". ", bridge_boys_top3$Item[i], " (", bridge_boys_top3$Construct[i],

") - Strength = ", round(bridge_boys_top3$Bridge_Strength[i], 3), "\n", sep="")

}

cat("\n Girls sample top 3 bridge items:\n")

bridge_girls_top3 <- head(bridge_girls, 3)

for(i in 1:nrow(bridge_girls_top3)) {

cat(" ", i, ". ", bridge_girls_top3$Item[i], " (", bridge_girls_top3$Construct[i],

") - Strength = ", round(bridge_girls_top3$Bridge_Strength[i], 3), "\n", sep="")

}

# 10.1 Bridge centrality visualization

cat("\n> Creating bridge centrality visualizations...\n")

bridge_dir <- file.path(GENDER_FIG_DIR, "bridge_centrality")

dir.create(bridge_dir, showWarnings = FALSE)

top_bridge_items <- bridge_comparison %>%

arrange(desc(Bridge_Strength_Diff_Abs)) %>%

head(10)

p_bridge <- ggplot(top_bridge_items,

aes(x = reorder(Item, Bridge_Strength_Diff),

y = Bridge_Strength_Diff,

fill = ifelse(Bridge_Strength_Diff > 0, "Boys", "Girls"))) +

geom_bar(stat = "identity") +

coord_flip() +

scale_fill_manual(values = c("Boys" = "#56B4E9", "Girls" = "#E69F00")) +

labs(title = "Gender Differences in Bridge Centrality",

subtitle = "Bridge items connect different constructs | Positive = Stronger in boys",

x = "Item", y = "Bridge Strength Difference (Boys - Girls)") +

theme_minimal() +

theme(legend.position = "none")

ggsave(file.path(bridge_dir, "08_bridge_centrality_differences.png"),

p_bridge, width = 12, height = 10, dpi = 300)

cat(" ✓ Bridge centrality plots saved\n")

# =============================================

# 11. SAVE ALL GENDER ANALYSIS RESULTS - CORRECTED

# =============================================

cat("\n> ============================================\n")

cat("> 11. SAVING ALL GENDER ANALYSIS RESULTS - CORRECTED\n")

cat("> ============================================\n")

cat("\n> Saving comprehensive gender analysis results...\n")

gender_analysis_summary <- list(

sample_info = list(

boys_n = n_boys,

girls_n = n_girls,

total_n = n_boys + n_girls,

boys_percentage = round(n_boys/(n_boys + n_girls)*100, 1),

girls_percentage = round(n_girls/(n_boys + n_girls)*100, 1),

gender_coding = "1 = Boys, 2 = Girls"

),

network_stats = list(

boys = boys_network$stats,

girls = girls_network$stats

),

networks = list(

boys_adjacency = boys_network$adj_matrix,

girls_adjacency = girls_network$adj_matrix

),

centrality = list(

boys = centrality_boys,

girls = centrality_girls,

comparison = centrality_combined

),

construct_analysis = construct_comparison,

bridge_analysis = list(

boys = bridge_boys,

girls = bridge_girls,

comparison = bridge_comparison

)

)

# CORRECTED: NCT results storage with proper status differentiation

if(nct_performed && nct_success) {

gender_analysis_summary$nct_results <- list(

performed = TRUE,

success = TRUE,

global_tests = if(!is.null(nct_summary)) nct_summary else "No results",

edge_differences = if(!is.null(sig_edge_df) && nrow(sig_edge_df) > 0)

sig_edge_df else "No significant edges after correction",

centrality_tests = if(!is.null(centrality_diff_results))

centrality_diff_results else "No results",

correction_method = "Holm"

)

} else if(nct_performed && !nct_success) {

gender_analysis_summary$nct_results <- list(

performed = TRUE,

success = FALSE,

error = nct_error_message

)

} else {

gender_analysis_summary$nct_results <- list(

performed = FALSE,

success = FALSE,

reason = nct_error_message %||% "Sample size too small for reliable NCT"

)

}

save(gender_analysis_summary,

file = file.path(GENDER_DIR, "09_gender_analysis_complete.RData"),

compress = "gzip")

write.csv(boys_data_complete,

file.path(GENDER_DIR, "09_boys_data_clean.csv"),

row.names = FALSE)

write.csv(girls_data_complete,

file.path(GENDER_DIR, "09_girls_data_clean.csv"),

row.names = FALSE)

cat(" ✓ All gender analysis results saved\n")

# =============================================

# 12. GENERATE GENDER ANALYSIS REPORT

# =============================================

cat("\n> ============================================\n")

cat("> 12. GENERATING GENDER ANALYSIS REPORT\n")

cat("> ============================================\n")

report_file <- file.path(GENDER_DIR, "10_gender_analysis_report.txt")

sink(report_file)

cat("GENDER DIFFERENCES IN NETWORK ANALYSIS - FINAL REPORT\n")

cat("=====================================================\n")

cat("Date: ", format(Sys.time(), "%Y-%m-%d %H:%M:%S"), "\n", sep="")

cat("Gender Coding: 1 = Boys, 2 = Girls\n")

cat("Analysis Script: 04_gender_differences.R (CORRECTED VERSION)\n\n")

cat("1. SAMPLE INFORMATION\n")

cat("=====================\n")

cat("Boys sample size (Gender=1): ", n_boys, " (",

round(n_boys/(n_boys + n_girls)*100, 1), "%)\n", sep="")

cat("Girls sample size (Gender=2): ", n_girls, " (",

round(n_girls/(n_boys + n_girls)*100, 1), "%)\n", sep="")

cat("Total gender sample: ", n_boys + n_girls, "\n\n", sep="")

cat("2. NETWORK CHARACTERISTICS BY GENDER\n")

cat("====================================\n")

cat("Boys network:\n")

cat(" - Number of edges: ", boys_network$stats$n_edges, "/", boys_network$stats$n_possible, "\n", sep="")

cat(" - Density: ", round(boys_network$stats$density * 100, 2), "%\n", sep="")

cat(" - Average edge weight: ", round(boys_network$stats$avg_weight, 3), " (SD = ",

round(boys_network$stats$sd_weight, 3), ")\n", sep="")

cat("\n")

cat("Girls network:\n")

cat(" - Number of edges: ", girls_network$stats$n_edges, "/", girls_network$stats$n_possible, "\n", sep="")

cat(" - Density: ", round(girls_network$stats$density * 100, 2), "%\n", sep="")

cat(" - Average edge weight: ", round(girls_network$stats$avg_weight, 3), " (SD = ",

round(girls_network$stats$sd_weight, 3), ")\n", sep="")

cat("\n")

cat("3. KEY GENDER DIFFERENCES\n")

cat("=========================\n\n")

cat("3.1 Item Means (Top 5 largest Cohen's d):\n")

for(i in 1:min(5, nrow(top_differences))) {

cat(" ", i, ". ", top_differences$Item[i],

": Boys M = ", round(top_differences$Mean_Boys[i], 2),

", Girls M = ", round(top_differences$Mean_Girls[i], 2),

" (d = ", round(top_differences$Cohen_d[i], 2), ", ",

top_differences$Effect_Size[i], ")\n", sep="")

}

cat("\n")

cat("3.2 Strength Centrality Differences (Top 5 largest):\n")

for(i in 1:min(5, nrow(top_centrality_diffs))) {

cat(" ", i, ". ", top_centrality_diffs$Item[i],

": Boys = ", round(top_centrality_diffs$Strength_Boys[i], 3),

", Girls = ", round(top_centrality_diffs$Strength_Girls[i], 3),

" (Diff = ", round(top_centrality_diffs$Strength_Diff[i], 3),

", ", top_centrality_diffs$Strength_Diff_Direction[i], ")\n", sep="")

}

cat("\n")

cat("3.3 Within-Construct Connectivity:\n")

for(i in 1:nrow(construct_comparison)) {

cat(" ", construct_comparison$Construct[i], ":\n", sep="")

cat(" Density: Boys = ", round(construct_comparison$Within_Density_Boys[i], 3),

", Girls = ", round(construct_comparison$Within_Density_Girls[i], 3),

" (Diff = ", round(construct_comparison$Within_Density_Diff[i], 3), ")\n", sep="")

cat(" Mean weight: Boys = ", round(construct_comparison$Within_Mean_Weight_Boys[i], 3),

", Girls = ", round(construct_comparison$Within_Mean_Weight_Girls[i], 3),

" (Diff = ", round(construct_comparison$Within_Weight_Diff[i], 3), ")\n", sep="")

}

cat("\n")

if(nct_performed && nct_success) {

cat("4. NETWORK COMPARISON TEST (NCT) RESULTS\n")

cat("========================================\n\n")

if(!is.null(nct_summary)) {

cat(" 4.1 Global Network Invariance:\n")

for(i in 1:nrow(nct_summary)) {

cat(" ", nct_summary$Test[i], ": p = ", nct_summary$p_value[i],

" (", nct_summary$Significance[i], ")\n", sep="")

}

cat("\n")

}

if(!is.null(sig_edge_df) && nrow(sig_edge_df) > 0) {

cat(" 4.2 Edge Differences (Holm-corrected p < 0.05):\n")

cat(" Total significant edges: ", nrow(sig_edge_df), "\n", sep="")

cat(" Top 3 most significant:\n")

for(i in 1:min(3, nrow(sig_edge_df))) {

cat(" ", i, ". ", sig_edge_df$Item1[i], " - ", sig_edge_df$Item2[i],

" (p_adj = ", round(sig_edge_df$p_value_adjusted[i], 4), ")\n", sep="")

}

} else {

cat(" 4.2 Edge Differences: No significant edges after Holm correction\n")

}

cat("\n")

if(!is.null(centrality_diff_results)) {

cat(" 4.3 Centrality Difference Tests:\n")

for(i in 1:nrow(centrality_diff_results)) {

cat(" ", centrality_diff_results$Measure[i], ": p = ",

centrality_diff_results$p_value[i], " (",

ifelse(centrality_diff_results$Significant[i], "Significant", "Not Significant"),

")\n", sep="")

}

}

cat("\n")

} else {

cat("4. NETWORK COMPARISON TEST (NCT)\n")

cat("================================\n")

cat(" NCT was not performed or failed:\n")

cat(" Reason: ", gender_analysis_summary$nct_results$reason %||%

gender_analysis_summary$nct_results$error %||% "Unknown", "\n\n")

}

cat("5. BRIDGE CENTRALITY\n")

cat("====================\n\n")

cat(" 5.1 Boys - Top 3 Bridge Items:\n")

for(i in 1:min(3, nrow(bridge_boys))) {

cat(" ", i, ". ", bridge_boys$Item[i], " (", bridge_boys$Construct[i],

") - Bridge Strength = ", round(bridge_boys$Bridge_Strength[i], 3), "\n", sep="")

}

cat("\n")

cat(" 5.2 Girls - Top 3 Bridge Items:\n")

for(i in 1:min(3, nrow(bridge_girls))) {

cat(" ", i, ". ", bridge_girls$Item[i], " (", bridge_girls$Construct[i],

") - Bridge Strength = ", round(bridge_girls$Bridge_Strength[i], 3), "\n", sep="")

}

cat("\n")

cat(" 5.3 Largest Gender Differences in Bridge Strength:\n")

top_bridge_diffs <- bridge_comparison %>% arrange(desc(Bridge_Strength_Diff_Abs)) %>% head(5)

for(i in 1:nrow(top_bridge_diffs)) {

cat(" ", i, ". ", top_bridge_diffs$Item[i],

": Boys = ", round(top_bridge_diffs$Bridge_Strength_Boys[i], 3),

", Girls = ", round(top_bridge_diffs$Bridge_Strength_Girls[i], 3),

" (Diff = ", round(top_bridge_diffs$Bridge_Strength_Diff[i], 3),

", ", top_bridge_diffs$Bridge_Strength_Direction[i], ")\n", sep="")

}

cat("\n")

cat("6. OUTPUT FILES GENERATED\n")

cat("=========================\n")

cat(" Tables:\n")

cat(" - 01_sample_info_boys_girls.csv\n")

cat(" - 02_descriptive_by_gender.csv\n")

cat(" - 03_adjacency_matrix_boys.csv\n")

cat(" - 03_adjacency_matrix_girls.csv\n")

cat(" - 05_centrality_comparison.csv\n")

if(nct_performed && nct_success) {

cat(" - 06_nct_global_results.csv\n")

cat(" - 06_edge_differences_pvalues_raw.csv\n")

cat(" - 06_edge_differences_pvalues_adjusted.csv\n")

if(!is.null(sig_edge_df) && nrow(sig_edge_df) > 0) {

cat(" - 06_significant_edge_differences_holm.csv\n")

}

cat(" - 06_centrality_difference_tests.csv\n")

}

cat(" - 07_construct_aggregated_comparison.csv\n")

cat(" - 08_bridge_centrality_comparison.csv\n")

cat("\n")

cat("7. INTERPRETATION FOR SCI MANUSCRIPT\n")

cat("====================================\n\n")

cat(" Key findings to report:\n")

cat(" 1. Global network structure: Boys network (",

round(boys_network$stats$density * 100, 2), "%, ",

"M = ", round(boys_network$stats$avg_weight, 3),

") vs Girls network (",

round(girls_network$stats$density * 100, 2), "%, ",

"M = ", round(girls_network$stats$avg_weight, 3), ")\n", sep="")

cat("\n")

cat(" 2. Items with largest gender differences in mean scores:\n")

for(i in 1:min(3, nrow(top_differences))) {

cat(" - ", top_differences$Item[i], " (d = ",

round(top_differences$Cohen_d[i], 2), ")\n", sep="")

}

cat("\n")

cat(" 3. Items with largest centrality differences:\n")

for(i in 1:min(3, nrow(top_centrality_diffs))) {

cat(" - ", top_centrality_diffs$Item[i], " (diff = ",

round(top_centrality_diffs$Strength_Diff[i], 3),

", ", top_centrality_diffs$Strength_Diff_Direction[i], ")\n", sep="")

}

cat("\n")

cat(" 4. Bridge centrality:\n")

cat(" - Boys: ", bridge_boys$Item[1], " (", bridge_boys$Construct[1], ")\n", sep="")

cat(" - Girls: ", bridge_girls$Item[1], " (", bridge_girls$Construct[1], ")\n", sep="")

cat("\n")

if(nct_performed && nct_success) {

cat(" 5. NCT results: ",

ifelse(nct_summary$p_value[1] < 0.05,

"Significant", "No significant"),

" difference in global network strength (p = ",

nct_summary$p_value[1], ")\n", sep="")

cat(" Edge weight invariance: p = ", nct_summary$p_value[2], "\n", sep="")

if(!is.null(sig_edge_df) && nrow(sig_edge_df) > 0) {

cat(" ", nrow(sig_edge_df), " edges significantly different after Holm correction\n", sep="")

}

}

cat("\n")

cat("ANALYSIS COMPLETED SUCCESSFULLY - CORRECTED VERSION\n")

cat("===================================================\n")

sink()

cat(" ✓ Gender analysis report saved\n")

# =============================================

# 13. COMPLETION MESSAGE

# =============================================

cat("\n> ============================================\n")

cat("> 13. GENDER ANALYSIS COMPLETE - CORRECTED VERSION\n")

cat("> ============================================\n")

cat("\n✅ GENDER DIFFERENCE ANALYSIS SUCCESSFULLY COMPLETED!\n\n")

cat("SUMMARY OF GENDER-SPECIFIC OUTPUTS:\n")

cat(" 1. ✓ Sample sizes: Boys =", n_boys, ", Girls =", n_girls, "\n")

cat(" 2. ✓ Descriptive statistics by gender\n")

cat(" 3. ✓ Separate network estimation for each gender\n")

cat(" 4. ✓ Gender-specific network visualizations\n")

cat(" 5. ✓ Centrality comparison between genders\n")

cat(" 6. ✓ Construct-aggregated analysis completed\n")

cat(" 7. ✓ Bridge centrality analysis (CORRECTED community vector)\n")

if(nct_performed && nct_success) {

cat(" 8. ✓ Network Comparison Test (NCT) performed with Holm correction\n")

} else {

cat(" 8. ⚠️ Network Comparison Test not performed: ",

gender_analysis_summary$nct_results$reason %||% "Unknown", "\n")

}

cat(" 9. ✓ All results saved for SCI publication\n\n")

cat("KEY INSIGHTS FOR SCI MANUSCRIPT:\n")

cat(" • Network density: Boys = ", round(boys_network$stats$density * 100, 2),

"%, Girls = ", round(girls_network$stats$density * 100, 2), "%\n", sep="")

cat(" • Average edge weight: Boys = ", round(boys_network$stats$avg_weight, 3),

", Girls = ", round(girls_network$stats$avg_weight, 3), "\n", sep="")

cat(" • Largest mean difference: ", top_differences$Item[1],

" (d = ", round(top_differences$Cohen_d[1], 2), ")\n", sep="")

cat(" • Largest centrality difference: ", top_centrality_diffs$Item[1],

" (diff = ", round(top_centrality_diffs$Strength_Diff[1], 3),

", ", top_centrality_diffs$Strength_Diff_Direction[1], ")\n", sep="")

cat(" • Top bridge item (Boys): ", bridge_boys$Item[1],

" (", bridge_boys$Construct[1], ")\n", sep="")

cat(" • Top bridge item (Girls): ", bridge_girls$Item[1],

" (", bridge_girls$Construct[1], ")\n", sep="")

if(nct_performed && nct_success) {

cat(" • NCT global strength: p = ", nct_summary$p_value[1], "\n", sep="")

}

cat("\nOUTPUT DIRECTORY:\n")

cat(" ", GENDER_DIR, "\n", sep="")

cat("\n> Gender analysis complete. Ready for SCI manuscript preparation.\n")

cat("> Please cite: NetworkComparisonTest package for NCT analysis.\n")
