# Supplementary material for: Psychosocial correlates of children’s football participation: a psychological network analysis of parental support, stereotypes, and dualistic passions
Source: Front Psychol. 2026 Jul 1;17:1897362. doi: 10.3389/fpsyg.2026.1897362 (PMC13368343; doi:10.3389/fpsyg.2026.1897362)
Supplement: Supplementary file 3 [file Table_2.DOCX]

| **A. Parental Sport Support Scale**  Adapted from the Perceived Social Support Scale – Family Subscale (Zimet et al., 1988)  **Instructions:** Please select the option that best describes your parents’ support regarding your sport participation (check “√”). | Strongly Disagree | Disagree | Neutral | Agree | Strongly Agree |
| --- | --- | --- | --- | --- | --- |
| PS1. 我的父母会尽可能的帮助我。  PS1. My parents try to help me as much as they can in my sport activities. |  |  |  |  |  |
| PS2. 我从父母那里得到了我需要的情感帮助和支持。  PS2. I get the emotional help and support I need from my parents regarding my sport participation. |  |  |  |  |  |
| PS3. 我可以和我的父母讨论我遇到的问题。  PS3. I can talk about my problems related to sport with my parents. |  |  |  |  |  |
| PS4. 我的父母愿意帮助我做出决定。  PS4. My parents are willing to help me make decisions about my sport involvement. |  |  |  |  |  |

| **B. Gender Stereotypes in Football Scale**  Adapted from Tomasetto et al. (2015) and Koivula (1999)  **Instructions:** Please select the option that best describes your opinion about football and gender (check “√”). | Strongly Disagree | Disagree | Neutral | Agree | Strongly Agree |
| --- | --- | --- | --- | --- | --- |
| GS1. 一般来说男生在足球运动中比女生更有天赋。  GS1. Generally speaking, boys are more talented in football than girls. |  |  |  |  |  |
| GS2. 一般来说女生在舞蹈运动中比男生更有天赋。  GS2. Generally speaking, girls are more talented in dance than boys. |  |  |  |  |  |
| GS3. 一般来说女生在舞蹈运动中会比在足球运动中表现得更好。  GS3. Generally speaking, girls perform better in dance than in football. |  |  |  |  |  |
| GS4. 一般来说男生在足球运动中会比在舞蹈运动中表现得更好。  GS4. Generally speaking, boys perform better in football than in dance. |  |  |  |  |  |

| **C. Passion for Football Scale**  Adapted from the Chinese Version of the Passion Scale (Zhao et al., 2015; Vallerand et al., 2003)  **Instructions:** Please select the option that best describes your feelings about football (check “√”). | Strongly Disagree | Disagree | Somewhat Disagree | Neutral | Somewhat | Agree | Strongly Agree |
| --- | --- | --- | --- | --- | --- | --- | --- |
| **Harmonious Passion** | | | | | | | |
| HP1. 我的足球活动和我生活中的其他活动是和谐共存的。  HP1. My football activities are in harmony with the other activities in my life. |  |  |  |  |  |  |  |
| HP3. 我在足球活动中发现的新事物让我更喜欢自己的足球活动。  HP3. The new things that I discover with my football activities allow me to appreciate them even more. |  |  |  |  |  |  |  |
| HP5. 我的足球活动反映了我喜欢的自身品质。  HP5. My football activities reflect the qualities I like about myself. |  |  |  |  |  |  |  |
| HP6. 我的足球活动让我能经历多样的体验。  HP6. My football activities allow me to live a variety of experiences. |  |  |  |  |  |  |  |
| HP8. 我的足球活动很好地融入了我的生活。  HP8. My football activities are well integrated in my life. |  |  |  |  |  |  |  |
| HP10. 我的足球活动和我生活的其他部分是和谐共存的。  HP10. My football activities are in harmony with other things that are part of me. |  |  |  |  |  |  |  |
| **Obsessive Passion** | | | | | | | |
| OP2. 我难以控制自己进行足球活动的冲动。  OP2. I have difficulties controlling my urge to engage in football activities. |  |  |  |  |  |  |  |
| OP4. 我对足球有种近乎痴迷的感觉。  OP4. I have almost an obsessive feeling for football. |  |  |  |  |  |  |  |
| OP7. 足球是唯一一件真正令我兴奋的事情。  OP7. Football is the only thing that really turns me on. |  |  |  |  |  |  |  |
| OP9. 如果可以，我会只进行我的足球活动。  OP9. If I could, I would only engage in football activities. |  |  |  |  |  |  |  |
| OP11. 足球是如此激动人心，以至于我有时候对它失去控制。  OP11. Football is so exciting that I sometimes lose control over it. |  |  |  |  |  |  |  |
| OP12. 我有一种足球在控制着我的感觉。  OP12. I have the impression that football controls me. |  |  |  |  |  |  |  |
| **Passion Criteria** | | | | | | | |
| PC13. 我用大量时间进行我的足球活动。  PC13. I spend a lot of time engaging in football activities. |  |  |  |  |  |  |  |
| PC14. 我喜欢我的足球活动。  PC14. I like my football activities. |  |  |  |  |  |  |  |
| PC15. 我的足球活动对我来说是重要的。  PC15. My football activities are important for me. |  |  |  |  |  |  |  |
| PC16. 我的足球活动是我的激情所在。  PC16. My football activities are a passion for me. |  |  |  |  |  |  |  |
| PC17. 我的足球活动构成了“我是谁”的一部分。  PC17. My football activities are part of who I am. |  |  |  |  |  |  |  |
| 1. **Football Participation** | | | | | | | |
| FP1. 在过去7天中（除学校安排的足球活动外），您有多少天参加了足球活动？  FP1. During the past seven days, how many days did you participate in football activities (excluding school-arranged football activities)? | | | | | | | |
| FP2. 在过去7天中（除学校安排的足球活动外），您平均每次参加足球活动多长时间？  FP2. During the past seven days, how long did you typically spend on each football activity session (excluding school-arranged football activities)? | | | | | | | |
